# Duplex Paternò–Büchi Isobaric Reagents for One-Step C=C-Resolved Lipid Isomer Quantification

**DOI:** 10.1021/jacs.6c13813

**Published:** 2026-09-28

**Authors:** Xi Chen, Gopal Reddy Ramidi, Nitya Shree, Dallas Freitas, Syuan-Ting Kuo, Huiying Guo, Luchen Wuyang, Aidan Slagter, Peiyu Cai, Ashok Polu, Mahua Choudhury, Xin Yan

**Affiliations:** † Department of Chemistry, 14736 Texas A&M University, College Station, Texas 77843, United States; ‡ Department of Pharmaceutical Sciences, Texas A&M University, College Station, Texas 77843, United States

## Abstract

Lipid isomeromics, the systematic structural and quantitative profiling of lipid isomers, is needed to determine how subtle variations in lipid structure contribute to metabolic regulation and disease. Carbon–carbon double-bond (C=C) positional isomers can have distinct biological functions despite sharing identical masses and sum compositions; however, their quantitative analysis remains challenging. Here, we introduce duplex Paternò–Büchi (PB)-derived isobaric mass tags, termed AMPE-tags, that integrate lipid derivatization, C=C positional-isomer identification, and relative quantification in a single photochemical step. The tags combine a PB-reactive ketone, an isotope-encoded methylpiperazine reporter, and a mass-balancing group within one molecular scaffold. Photoirradiation installs the tag directly at lipid C=C bonds to form oxetane-containing PB products. Higher-energy collisional dissociation of intact PB products generates reporter ions for total-level quantification, whereas collision-induced dissociation produces C=C position-specific diagnostic ions that retain the reporter-bearing tag. Subsequent MS^3^ fragmentation of these diagnostic ions releases reporter ions directly associated with individual positional isomers, enabling isomer-level quantification without chromatographic resolution of C=C positional isomers. The duplex tags provided accurate relative quantification of unsaturated fatty acids over a 200-fold concentration-ratio range and in structurally diverse mixtures containing PB regioisomers and minor photochemical byproducts. Application to visceral adipose tissue from mice fed chow or high-fat diets identified 18 unsaturated fatty acids and 37 C=C positional isomers, revealing diet-associated isomer remodeling that was masked at the total-fatty-acid level, including opposing changes among FA 18:1 positional isomers. These PB-derived isobaric reagents provide a chemical platform for duplex lipid isomeromics and enable biologically informative quantification beyond conventional sum-composition lipid analysis.

## Graphical abstract

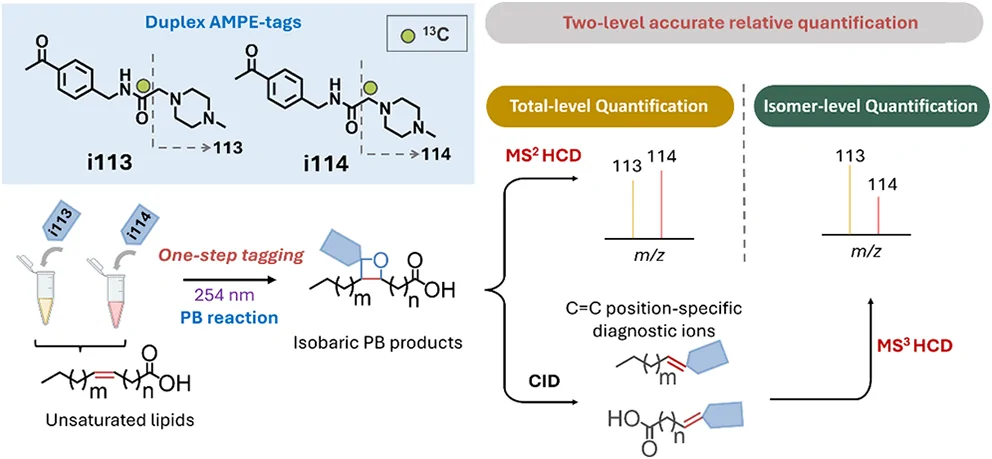

## Introduction

Lipids are essential biomolecules involved in energy storage, membrane organization, and cellular signaling, and perturbations in lipid composition are associated with numerous physiological and pathological processes.^1–3^ Beyond changes in bulk lipid abundance, subtle structural variations among lipid isomers can alter membrane properties, metabolic flux, and signaling functions. Here, we use the term *lipid isomeromics* to describe the systematic structural characterization and quantitative profiling of lipid isomers in biological systems. Among the diverse forms of lipid structural isomerism, carbon–carbon double-bond (C=C) positional isomers are particularly important because they share identical elemental compositions and masses while often exhibiting distinct biological activities. Altered distributions of lipid C=C positional isomers have been associated with cancer, type 2 diabetes, obesity, and other metabolic disorders.^4–9^ Therefore, analytical strategies that can identify and quantify lipid C=C positional isomers are needed to advance lipid isomeromics and clarify the biological roles of structurally resolved lipid species.

Mass spectrometry (MS) has become a central platform for lipid analysis because of its high sensitivity, selectivity, and throughput.^10–14^ However, conventional MS-based lipidomics remains poorly equipped to distinguish and quantify C=C positional isomers. These isomers have nearly identical physicochemical properties, co-ionize readily, and often produce similar fragmentation patterns. In particular, collision-induced dissociation (CID), the most widely used tandem MS method, generally does not cleave C=C bonds efficiently, thereby providing limited positional information.^15,16^ To address this limitation, specialized ion activation methods, including ozone-induced dissociation (OzID),^17,18^ electron-impact excitation of ions from organics (EIEIO),^19^ and ultraviolet photodissociation (UVPD),^20–22^ as well as derivatization-based approaches such as ozonolysis,^23–26^ epoxidation,^9,27–32^ aziridination,^33–37^ methoxylation,^38^ methylthiolation,^39^ cross-metathesis,^40^ peroxidation,^41^ and Paternò–Büchi (PB)-based reactions^42,43^ have been developed for C=C bond localization.

Among these approaches, PB chemistry offers a particularly attractive foundation for lipid isomeromics. The PB reaction is a [2 + 2] photocycloaddition between a photoexcited carbonyl group and a C=C bond that converts the double bond into an oxetane ring.^44,45^ Subsequent fragmentation of the oxetane ring generates C=C position-specific diagnostic ions. PB reactions have been applied to a broad range of lipid classes, including fatty acids (FAs), glycerophospholipids (GPLs), cholesteryl esters (CEs), and triacylglycerols (TAGs), and are compatible with direct-infusion MS, LC–MS/MS, mass spectrometry imaging, and liquid extraction surface analysis.^5,6,42,46–52^ Despite these advantages for structural characterization, PB-based workflows have not yet provided a general strategy for direct reporter-ion quantification of individual lipid C=C positional isomers.

Quantification presents a distinct and more difficult challenge than structural assignment. In this work, total-level quantification refers to measurement of the combined abundance of all C=C positional isomers sharing the same lipid composition, whereas isomer-level quantification refers to measurement of individual C=C positional isomers within that population. Conventional MS signal intensities do not directly reflect analyte abundance because they are affected by matrix effects, ionization efficiency, and instrumental variability.^53^ Quantification based on diagnostic fragment ion intensities typically requires authentic standards and isomer-specific calibration curves, which are unavailable for many endogenous lipid isomers and can be affected by changes in sample matrix or instrument conditions.^27,43^ Internal-standard-based approaches can improve quantitative performance but often rely on chromatographic separation, which remains difficult for closely related positional isomers.^54^ Consequently, direct and robust quantification of lipid C=C positional isomers remains an important unmet need.

Isobaric mass tagging provides an attractive framework for relative quantification because samples labeled with chemically equivalent tags can be combined before MS analysis, thereby minimizing variability arising from sample handling and ionization. Originally developed for proteomics, isobaric tags such as tandem mass tags (TMT) and isobaric tags for relative and absolute quantification (iTRAQ) produce distinct reporter ions upon tandem MS while retaining identical precursor masses.^55–59^ Recent studies have adapted isobaric labeling to lipid analysis, including workflows that combine SUGAR tags with meta-chloroperoxybenzoic acid epoxidation, aziridination followed by TMT labeling, and diazobutanone-assisted isobaric labeling for relative quantification of multiple lipid classes.^36,60–68^ These studies have established the potential of isobaric strategies for lipid quantification. However, current workflows generally require separate C=C derivatization and isobaric-labeling steps. In many existing workflows, reporter ions are generated from intact tagged lipids and therefore primarily report the combined abundance of isomeric lipids at the total level. Quantification of individual isomers consequently often still relies on chromatographic resolution or indirect estimation from isomer-diagnostic fragment intensities.

We reasoned that PB chemistry could overcome these limitations through rational reagent design. In addition to providing a photochemical handle for C=C derivatization, the carbonyl-containing PB reagent can be structurally elaborated to incorporate an isotope-encoded reporter and a mass-balancing group. Critically, PB-derived diagnostic ions can retain the reporter-bearing tag scaffold after oxetane-ring cleavage. This feature creates an opportunity to generate reporter ions not only from intact PB products for total-level quantification but also from isomer-specific diagnostic ions for direct isomer-level quantification.

Here, we report a new class of duplex PB-derived isobaric mass tags, termed AMPE-tags, for one-step lipid derivatization, C=C positional-isomer identification, and relative quantification. The AMPE-tags integrate a PB-reactive ketone, an isotope-encoded methylpiperazine reporter, and a mass-balancing group within a single reagent. Upon photoirradiation, the tags react directly with lipid C=C bonds to form PB products. Higher-energy collisional dissociation (HCD) of intact PB products releases reporter ions that quantify lipids at the total level, whereas CID generates C=C position-specific diagnostic ions that retain the tag structure. Subsequent HCD of these diagnostic ions in MS^3^ releases reporter ions directly associated with individual C=C positional isomers. Using the duplex AMPE-tag pair, we demonstrate accurate relative quantification of FAs at both the total and isomeric levels without requiring chromatographic resolution of C=C positional isomers. We further apply this strategy to profile diet-associated changes in FA composition and C=C positional-isomer distributions in visceral adipose tissue (VAT) from mice fed with chow or high-fat diets. This work establishes PB-derived isobaric reagents as a chemical platform for duplex lipid isomeromics.

## Results and Discussion

### Rational Design of Duplex PB-Derived Isobaric Reagents

We designed AMPE-tags as a new class of duplex PB-derived isobaric reagents that integrate lipid derivatization, C=C positional-isomer identification, and relative quantification within a single molecular scaffold (Figure 1). The reagent design was guided by three requirements: efficient photochemical reactivity toward lipid C=C bonds, generation of C=C position-specific diagnostic ions, and retention of an isotope-encoded reporter on those diagnostic ions to enable direct isomer-level quantification. Conventional isobaric tags comprise a reactive group, a reporter group, and a mass-balancing group. In the AMPE-tag design, the PB-reactive ketone serves not only as the lipid-labeling group but also as the structural probe that converts a C=C bond into a fragmentable oxetane ring.

**Figure 1:**
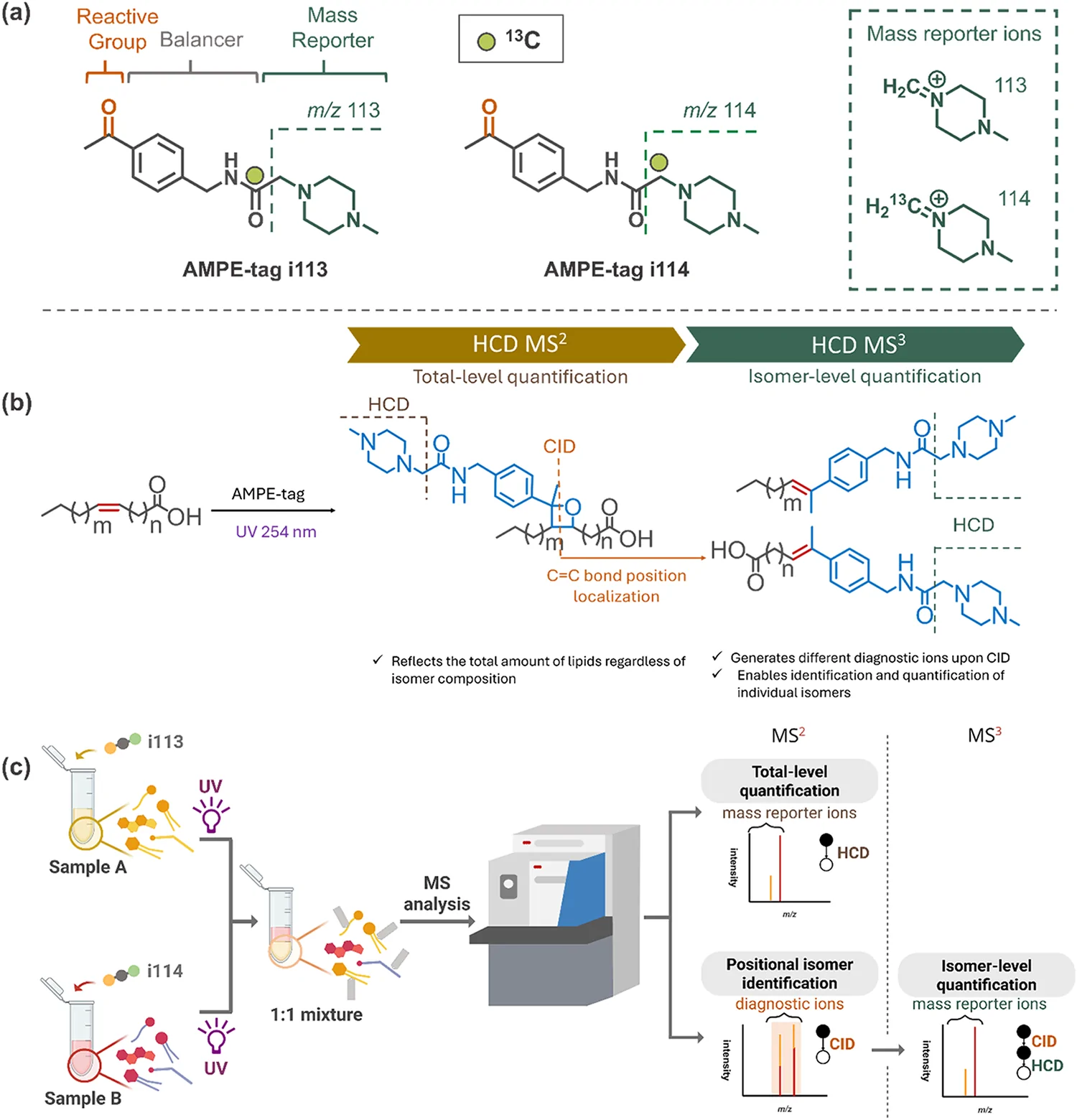
(a) Design of the duplex isobaric AMPE-tag. The whole tag is divided into three parts: reactive group, mass balancer and mass reporter. Places of ^13^C labeling are shown here for AMPE-i113 and AMPE-i114, respectively. (b) Principle of using AMPE-tags for lipid C=C bond positional isomer identification and quantification. C=C bond positions are revealed upon CID, while relative quantification is achieved by comparing the intensities of mass reporter ions. HCD at MS^2^ shows the relative abundance of lipids at the total level, while HCD at MS^3^ shows the relative abundance of lipids at the isomeric level. (c) Application of the duplex AMPE-tag i113 and i114 to the analysis of two samples. After labeling separately, the reaction solutions are equally mixed before MS analysis to reduce the matrix effect.

PB chemistry was selected because the carbonyl-containing reagent can be structurally elaborated without compromising its ability to undergo [2 + 2] photocycloaddition with lipid C=C bonds. Upon photoirradiation, the reaction of the ketone with a lipid C=C bond forms an oxetane ring. Subsequent CID cleavage of this ring produces diagnostic ions that encode the original C=C bond position. Importantly, the PB-derived diagnostic ions retain the reporter-bearing portion of the AMPE-tag. This feature distinguishes the present design from workflows in which structural derivatization and isobaric labeling are introduced independently and enables a two-tiered fragmentation strategy: HCD of intact PB products releases reporter ions for total-level quantification, whereas HCD of isomer-specific diagnostic ions generated by CID releases reporter ions directly associated with individual C=C positional isomers.

1-(4-(Aminomethyl)­phenyl)­ethan-1-one (AMPE), an acetophenone-derived PB reagent, was selected as the reactive core. Conjugation of the ketone to the aromatic ring favors formation of the reactive triplet excited state and has been reported to promote PB reactivity while suppressing competing photochemical pathways.^45^ The aminomethyl substituent provides a synthetically accessible site for coupling to the isobaric-tagging module. Placement of the amine on a methylene spacer, rather than directly on the aromatic ring, was intended to minimize electron-donating effects that could attenuate the photoreactivity of the acetophenone moiety.^69^

The AMPE-tag incorporates a methylpiperazine-derived reporter group connected to the PB-reactive moiety through an amide-containing balancing region. The reporter group design is intended to provide quantitative functionality rather than simply enhance the absolute detection sensitivity. Reporter ion-based quantification requires a specifically designed structure that undergoes efficient and reproducible cleavage to generate abundant, stable charged reporter ions, as exemplified by established isobaric tags such as TMT and iTRAQ. Methylpiperazine was selected because it readily generates a low-mass reporter ion upon HCD and resembles reporter architectures used in established peptide isobaric tags.

Duplex encoding was achieved by placing a single ^13^C atom at complementary positions in the reporter and balancing regions. In AMPE-tag i113, the ^13^C label is positioned at the amide carbonyl within the balancing region, whereas in AMPE-tag i114, the ^13^C label is located within the methylpiperazine-containing reporter region. The two reagents, therefore, have identical overall masses but generate reporter ions at *m*/*z* 113 and 114, respectively. This isotope-placement strategy establishes the duplex format while preserving a modular architecture that can be expanded in future designs through additional stable-isotope encoding and balancing-group modification.

The AMPE-tags were synthesized from 1-methylpiperazine and ethyl 2-bromoacetate, followed by hydrolysis to afford 2-(4-methylpiperazin-1-yl)­acetic acid. Coupling of this intermediate with AMPE yielded the final tag scaffold, and the duplex isotopic variants were prepared from isotopically differentiated bromoacetate precursors. Detailed synthetic procedures and characterization data are provided in the Supporting Information S1 and S2. During route development, activation of the carboxylic acid with *N,N′*-disuccinimidyl carbonate provided a cleaner route to the final AMPE-tag than HATU-mediated coupling by avoiding persistent coupling-reagent impurities and the need for DMF-based purification.

We next examined the dissociation behavior of the duplex reagents to establish their suitability for isobaric quantification. HCD generated the expected reporter ions at *m*/*z* 113 and 114, and the two isotopic tags exhibited comparable reporter-ion yields under optimized fragmentation conditions (Figure S7). Moreover, reporter-ion intensity ratios closely tracked the molar ratios of AMPE-tag i113 and i114 across the tested range (Figure S8), demonstrating that isotope encoding did not introduce a measurable bias in reporter generation. These results establish the AMPE-tags as chemically equivalent duplex reagents designed to connect PB-enabled C=C localization with hierarchical quantification at the total and isomeric levels.

### One-Step PB Tagging Enables Lipid C=C Positional-Isomer Identification

We first evaluated whether the AMPE scaffold could support efficient one-step photochemical labeling of unsaturated lipids while preserving the characteristic Paternò–Büchi (PB) fragmentation behavior required for C=C positional-isomer identification. A nonisotope-encoded AMPE-tag was initially used to simplify characterization of the PB products. PC 18:1(9)/18:1(9) was selected as a representative substrate because it contains two Δ9 double bonds and produces readily interpretable PB-derived diagnostic ions. Lipid and tag were combined in acetonitrile and subjected to UV irradiation in a microflow photoreactor under optimized conditions. Microflow irradiation was used to promote efficient photochemical conversion while minimizing reaction time and competing photochemical pathways.^6^

Before irradiation, the spectrum contained signals corresponding to the unreacted AMPE-tag, native PC, and a noncovalent lipid–tag adduct. CID of the latter generated only underivatized PC fragments, confirming that this species did not represent a covalent PB product. In contrast, UV irradiation produced a dominant ion at *m*/*z* 1075.8, consistent with the formation of a mono-PB adduct of PC 18:1(9)/18:1(9) (Figure 2a–d). Under the optimized conditions (see Supporting Information S4), the apparent conversion of PC to the PB product reached 78% (Figure S14). Although the substrate contains two C=C bonds, mono-PB addition was the predominant reaction pathway, providing a simplified product distribution suitable for subsequent structural and quantitative analysis.

**Figure 2:**
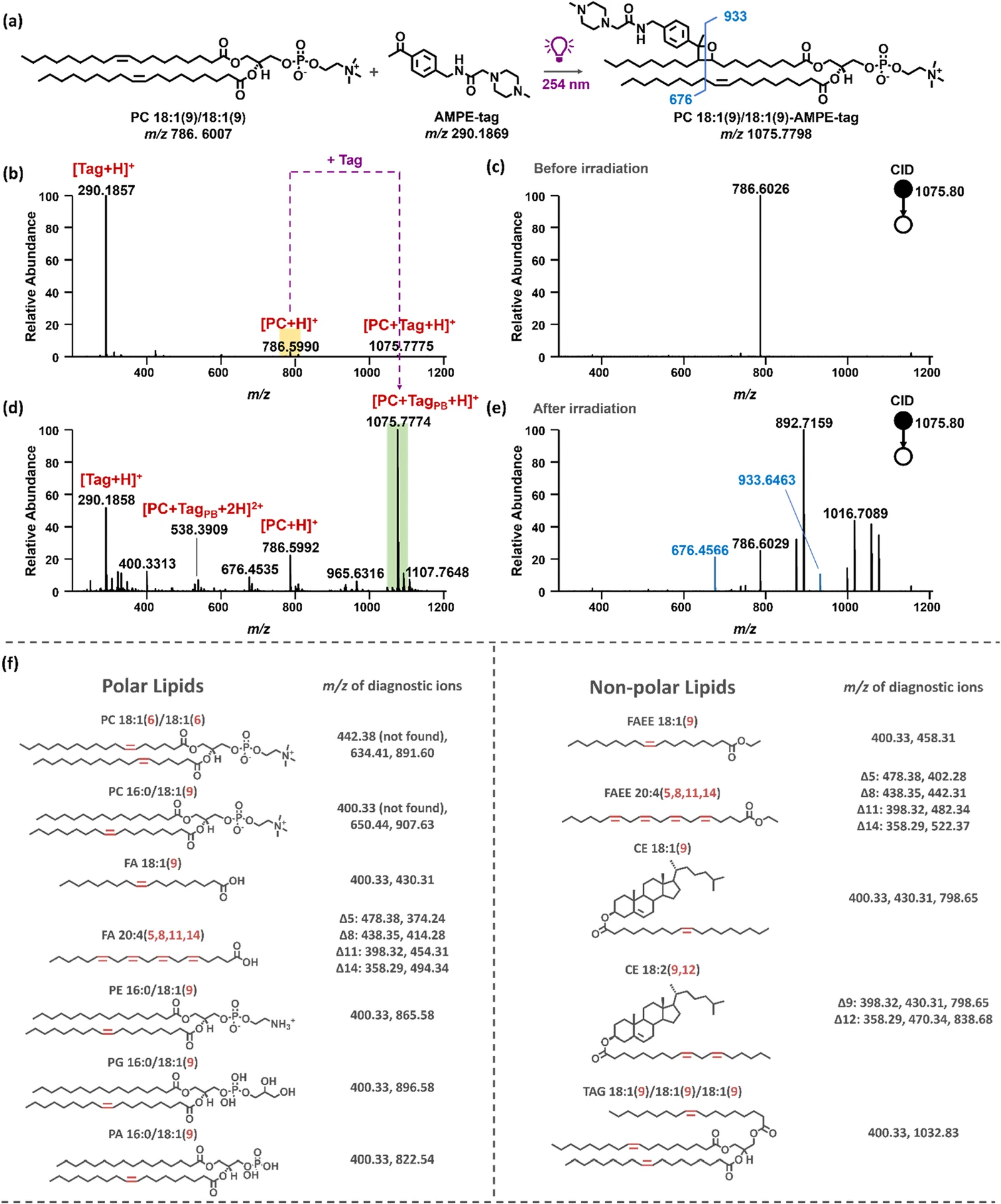
One-step PB tagging by the nonisotope-encoded AMPE reagent enables lipid C=C positional-isomer identification. (a) Photoinduced PB reaction of AMPE-tag with PC 18:1(9)/18:1(9), forming the oxetane-containing PB product. (b–e) Mass spectra of the PC/AMPE-tag reaction mixture and CID spectra of the ion at *m*/*z* 1075.8 acquired before and after UV irradiation. UV irradiation produces the covalent mono-PB product, which generates phosphocholine headgroup-loss ions and C=C position-specific diagnostic ions upon CID. (f) Representative application of AMPE-mediated PB tagging to unsaturated lipids from multiple classes, demonstrating generation of diagnostic ions for C=C bond localization.

CID of the PB product generated the expected phosphocholine headgroup-loss ions together with oxetane-cleavage products diagnostic of the Δ9 C=C position (Figure 2e). As expected for PB chemistry, formation of two oxetane regioisomers produced complementary diagnostic ions. Although headgroup fragmentation competed with diagnostic-ion formation for PC, the observed diagnostic ions enabled unambiguous localization of the Δ9 double bonds. Detailed assignments of low-abundance diagnostic ions, multiply charged products, and minor photochemical byproducts are provided in Supporting Information S5.1.

We next examined the applicability of AMPE-mediated PB tagging to representative unsaturated lipid classes. The AMPE scaffold also functions as a charge tag, facilitating positive-ion detection of lipids that are otherwise weakly ionized under conventional electrospray conditions. PB products from fatty acids, fatty acid ethyl esters, cholesteryl esters, glycerophospholipids, and triacylglycerols generated C=C position-specific diagnostic ions upon CID (Figures S17–S18). For lipid classes lacking dominant headgroup-loss pathways, diagnostic ions constituted among the most abundant product ions. In addition, lipids containing multiple C=C bonds generated interpretable diagnostic-ion patterns, allowing unambiguous assignment of multiple double-bond positions. These results establish that AMPE-tags provide a broadly applicable, single-step route to PB-enabled C=C positional-isomer identification and provide the structural foundation for the hierarchical quantification strategy described below.

### Hierarchical Quantification at the Total and Isomeric Levels

We next evaluated whether AMPE-tags could support hierarchical relative quantification at both the total and isomeric levels. These two readouts provide complementary biological information. Total-level quantification measures the combined abundance of all C=C positional isomers sharing the same lipid composition and therefore reveals changes in the overall lipid pool. In contrast, isomer-level quantification resolves the abundance of individual positional isomers and can reveal pathway- or enzyme-associated remodeling that may be masked when the isomers are considered collectively.

HCD of an intact PB product releases isotope-encoded reporter ions that report the combined abundance of the corresponding lipid composition. In contrast, CID first generates C=C position-specific diagnostic ions that retain the reporter-bearing AMPE-tag scaffold; subsequent HCD of an isolated diagnostic ion in MS^3^ releases reporter ions directly associated with the corresponding positional isomer. This tandem fragmentation sequence therefore enables total- and isomer-level relative quantification from the same tagged lipid population.

FA 18:1(9) and FA 18:1(6) were selected as model analytes owing to their ability to generate abundant diagnostic ions for positional isomer identification. The dissociation behavior of AMPE-tag-labeled FAs was investigated and optimized as well (see Supporting Information S6). Sample and reference mixtures containing the two isomers were independently labeled with AMPE-tag i113 and AMPE-tag i114, respectively, followed by 1:1 mixing before MS analysis (Figure 3a). The resulting PB products were detected at *m*/*z* 573.4. CID generated the expected diagnostic ions for the Δ9 and Δ6 isomers, including ions at *m*/*z* 401.3 and 431.3 for FA 18:1(9) and ions at *m*/*z* 389.3 and 443.3 for FA 18:1(6) (Figure 3b,c). HCD of the intact PB product at *m*/*z* 573.4 generated reporter ions at *m*/*z* 113 and 114 for total-level quantification, whereas HCD of each isomer-specific diagnostic ion generated the corresponding reporter-ion pair for isomer-level quantification (Figure 3c).

**Figure 3:**
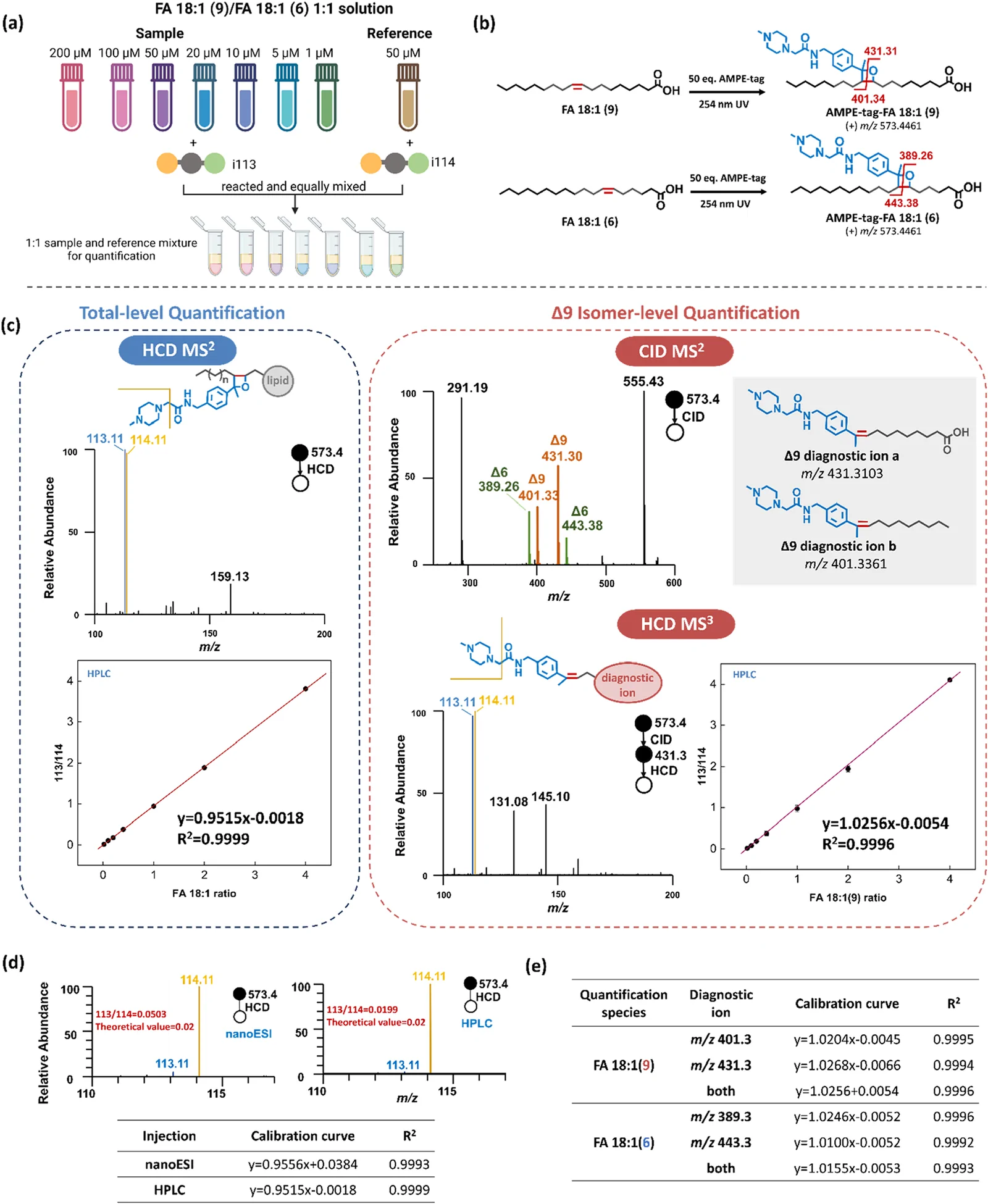
Quantification of FA 18:1(9)/18:1(6) 1:1 mixture with the duplex AMPE-tags. (a) Illustration of building calibration curves with FA 18:1(9)/FA 18:1(6) 1:1 solutions from 1 to 200 μM as samples and 50 μM as references. (b) Reaction of isobaric AMPE-tags with FA 18:1(9) and FA 18:1(6); diagnostic ions in MS analysis were labeled in red. (c) Quantification at the total-level and isomer-level at MS^2^ and MS^3^, respectively. Δ9 isomer results were demonstrated here, while Δ6 isomer results could be found in Supporting Information S8.1. (d) MS^2^ HCD spectra of the PB product at *m*/*z* 573 with sample at 1 μM and reference at 50 μM with nanoESI and HPLC infusion. With the separation of HPLC, the interference was excluded, and the quantification result was more accurate. Interference at low concentrations (less than 10 μM) was observed for direct infusion, causing distortion of the calibration curve intercept. With the separation of contaminants and side products with HPLC, the interference effect was alleviated. (e) A summary of calibration curves for quantifying FA 18:1 at the isomer level. With all the calibration curves showing good linearity with slopes close to 1 and intercepts close to 0, the feasibility of quantification of the isobaric tags was validated, and the concentration ratio of the fatty acids could be directly read from the mass reporter ion intensity ratio.

The total-level response was evaluated using FA 18:1(9)/FA 18:1(6) mixtures at a fixed 1:1 isomer ratio over sample-to-reference concentration ratios of 0.02–4. Direct-infusion nanoESI-MS provided the expected reporter-ion response at moderate and high analyte concentrations; however, quantification became less accurate at the lowest concentrations because chemical background and minor PB-derived byproducts contributed to the intact-product signal. Although a linear calibration curve could still be obtained, the intercept deviated from 0 as expected (Figure 3d). Incorporation of LC separation resolved the PB products from these interferences and substantially improved total-level quantification. The resulting LC–MS calibration curve exhibited a slope of 0.9515, an intercept of −0.0018, and an *R* ^2^ value of 0.9999 across the tested concentration-ratio range (Figure 3c). These data demonstrate that the reporter-ion ratio derived from the intact PB product accurately reflects the combined abundance of FA 18:1 positional isomers after removal of coeluting chemical interference.

We then examined isomer-level quantification by isolating the Δ9- or Δ6-specific diagnostic ions generated by CID and subjecting them to HCD in MS^3^. In contrast to the total-level measurement, direct-infusion nanoESI-MS was sufficient for isomer-level quantification because isolation of the diagnostic ion provided an additional level of chemical selectivity before reporter-ion generation (see Supporting Information S8.1). For both FA 18:1(9) and FA 18:1(6), reporter-ion ratios obtained from their corresponding diagnostic ions tracked the expected sample-to-reference concentration ratios with excellent linearity (*R* ^2^ > 0.995), slopes close to unity, and intercepts near zero (Figure 3e). Where two complementary diagnostic ions were available for a given isomer, the final isomer-level ratio was calculated from the averaged reporter-ion ratios obtained from both ions. These results establish that PB-derived diagnostic ions can serve not only as structural markers for C=C localization but also as reporter-bearing intermediates for direct relative quantification of individual lipid positional isomers.

The hierarchical nature of this workflow is therefore important for lipid isomeromics. Total-level measurements establish whether the overall abundance of a lipid pool changes, whereas isomer-level measurements determine whether that change reflects proportional variation across all isomers or selective remodeling of specific isomeric species. The AMPE-tag platform links lipid structural assignment and relative quantification within a single labeling reaction and provides two complementary levels of quantitative information from the same tagged analyte population. It should also be noted that purification of the tags is critical for accurate quantification. Residual impurities from the synthesis process, such as triethylamine, can alter tag reactivity and compromise quantification accuracy (see Supporting Information S7). Additional validation using FA 18:1(9)/FA 18:1(6) mixtures at various ratios, polyunsaturated FAs, FA derivatives, and structurally diverse FA mixtures is described below, as well as in Supporting Information S8. The limit of detection (LOD) was determined at 50 nM (see Supporting Information S9).

It should be noted that although AMPE-mediated PB tagging generated C=C position-specific diagnostic ions from multiple lipid classes, the most extensive quantitative validation in this study was performed using fatty acids, which produce abundant PB diagnostic ions with limited competing fragmentation. Quantitative analysis was also demonstrated for representative fatty acid ethyl ester and cholesteryl ester species (Figures S29 and S30), indicating that the reporter-ion-based strategy is applicable beyond free fatty acids. In contrast, more structurally complex intact lipids, including glycerophospholipids and triacylglycerols, were evaluated here primarily for C=C localization. Full quantitative validation for these lipid classes will require future systematic evaluation because competing headgroup loss, neutral loss, and acyl-chain fragmentation may influence PB diagnostic-ion formation and reporter-ion responses.

### Quantitative Accuracy and Scope in Structurally Diverse FA Mixtures

We next assessed the robustness of the AMPE-tag strategy in a structurally diverse FA mixture containing species with different chain lengths, degrees of unsaturation, and C=C positional-isomer compositions. The mixture comprised FA 16:1(9), FA 18:1(9), FA 18:1(6), FA 18:2­(9,12), FA 18:3­(9,12,15), and FA 20:4­(5,8,11,14). Each FA was present at 20 μM in the sample mixture, whereas an otherwise identical reference mixture contained each analyte at 10 μM. The sample and reference were independently labeled with AMPE-tag i113 and AMPE-tag i114, respectively, combined at equal volumes after photoirradiation, and analyzed by LC–MS/MS in triplicate.

LC separation was used to resolve PB products from excess AMPE-tags and minor photochemical byproducts before tandem MS analysis (see Supporting Information S10). This separation also revealed the intrinsic product complexity of PB chemistry. For example, the extracted-ion chromatogram of AMPE-tag-labeled FA 16:1(9) contained three partially resolved features (Figure 4a–d). CID analysis indicated that the latter two peaks corresponded to the expected complementary PB regioisomers, each yielding a distinct Δ9 diagnostic ion, whereas the earliest-eluting feature produced few diagnostic ions and was assigned as a minor photochemical side product. Thus, a single lipid can generate multiple PB-derived species with distinct chromatographic and fragmentation behaviors.

**Figure 4:**
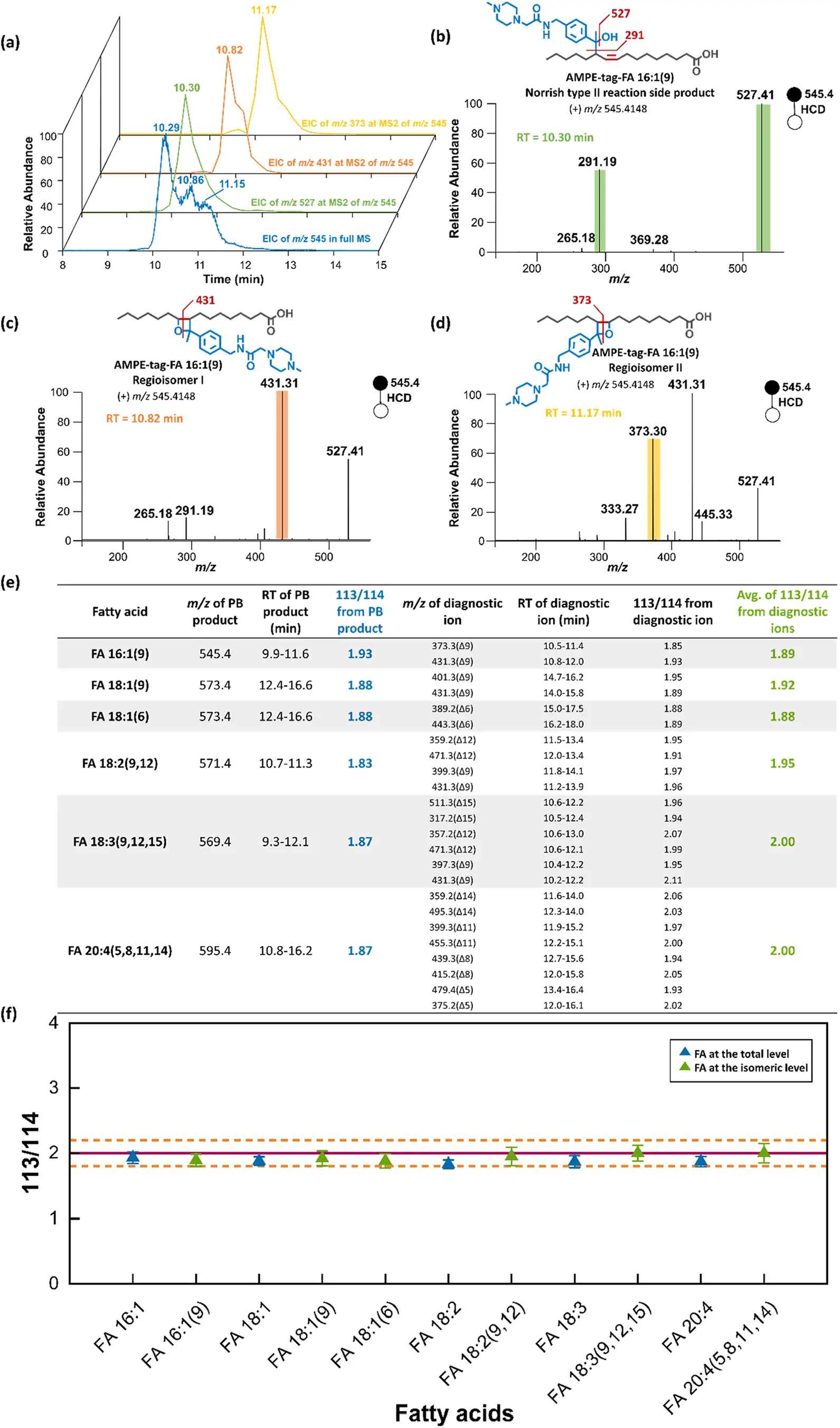
Accurate total- and isomer-level quantification of a structurally diverse artificial fatty acid mixture. (a) Extracted-ion chromatogram of the AMPE-tag-labeled FA 16:1(9) PB product at *m*/*z* 545.4. Three partially resolved chromatographic features were detected between 9.9 and 11.6 min. (b–d) CID spectra acquired at retention times of 10.30, 10.82, and 11.17 min, respectively. The latter two peaks correspond to complementary PB regioisomers that generate distinct Δ9 diagnostic ions, whereas the earliest-eluting species produces few diagnostic ions and is assigned as a minor photochemical side product. Structures of the corresponding products are shown above the spectra. (e) Summary of retention times, PB-product and diagnostic-ion *m*/*z* values, and measured reporter-ion ratios for the six FA standards and their positional isomers. (f) Measured i113/i114 reporter-ion ratios for total-level FA quantification and isomer-level quantification. Blue symbols denote total FA measurements and green symbols denote positional-isomer measurements. The red line indicates the expected ratio of 2.0; orange lines indicate ± 10% deviation. All measurements fell within this range, demonstrating accurate relative quantification despite PB regioisomer formation and minor side products.

Despite this product complexity, reporter-ion ratios remained consistent with the expected 2-fold concentration difference between the sample and reference mixtures. At the total level, measured reporter-ion ratios for the six FAs ranged from 1.83 to 1.93. Isomer-level measurements, obtained by HCD of the corresponding diagnostic ions in MS^3^, yielded ratios from 1.88 to 2.00 (Figure 4e,f). For FAs that generated multiple complementary diagnostic ions, the final isomer-level ratio was calculated by averaging the reporter-ion ratios obtained from the corresponding diagnostic-ion pair.

These results demonstrate that the duplex AMPE-tags tolerate the formation of PB regioisomers and minor side products while maintaining accurate relative quantification across structurally diverse unsaturated FAs. More broadly, the data show that the reporter-ion readout is preserved across lipid species containing multiple C=C bonds and remains compatible with both total- and isomer-level analysis in a mixed-lipid setting.

### Application of Duplex Lipid Isomeromics to Diet-Associated FA Isomer Remodeling in Murine Visceral Adipose Tissue

We next applied the duplex AMPE-tag platform to characterize diet-associated changes in fatty acid (FA) composition and C=C positional-isomer distributions in murine visceral adipose tissue (VAT). VAT is a major site of triacylglycerol storage and therefore provides a relevant system for examining remodeling of esterified FA pools in response to dietary fat intake.^70,71^ We focused the VAT application on fatty acids released by saponification. This design was chosen because VAT is enriched in triacylglycerols, and hydrolysis provides a direct readout of the fatty acyl-chain composition of this major lipid storage pool. In addition, fatty acids generate abundant PB diagnostic ions without strong competition from headgroup fragmentation, allowing rigorous evaluation of both total-level and isomer-level quantification in a biological matrix.

Lipids were extracted from VAT collected from mice fed either a chow diet (CD) or a high-fat diet (HFD) for 14 weeks (three biological replicates per group), which is a common model to study obesity and relevant diseases, including hyperlipidemia, diabetes, liver steatosis, and metabolic syndrome.^72–76^ Triacylglycerols constitute the predominant lipid class in VAT^77^ (Table S2), and the extracts were subjected to saponification^25,78,79^ to release constituent fatty acyl chains before AMPE-tag derivatization and LC–MS/MS analysis (Table S3).

To enable relative quantification across biological samples, VAT extracts from three chow diet-fed mice (CD1–CD3) and three high-fat diet-fed mice (HFD1–HFD3) were analyzed. Each biological extract was labeled with AMPE-tag i114, whereas an aliquot of the CD1 extract was independently labeled with AMPE-tag i113 and used as a common reference for all measurements (Figure 5a). CD1 was selected as the common reference to enable direct comparison of reporter-ion ratios across the complete sample set. The i113/i114 ratio obtained from independently labeled CD1 aliquots was close to unity, supporting comparable labeling efficiency and reporter-ion generation by the duplex tags in the biological matrix (Figure S33). C=C positional-isomer assignments were obtained from CID-generated diagnostic ions, whereas reporter ions released from intact PB products and isomer-specific diagnostic ions provided total- and isomer-level quantitative readouts, respectively. Three technical replicates were acquired for each biological sample.

**Figure 5:**
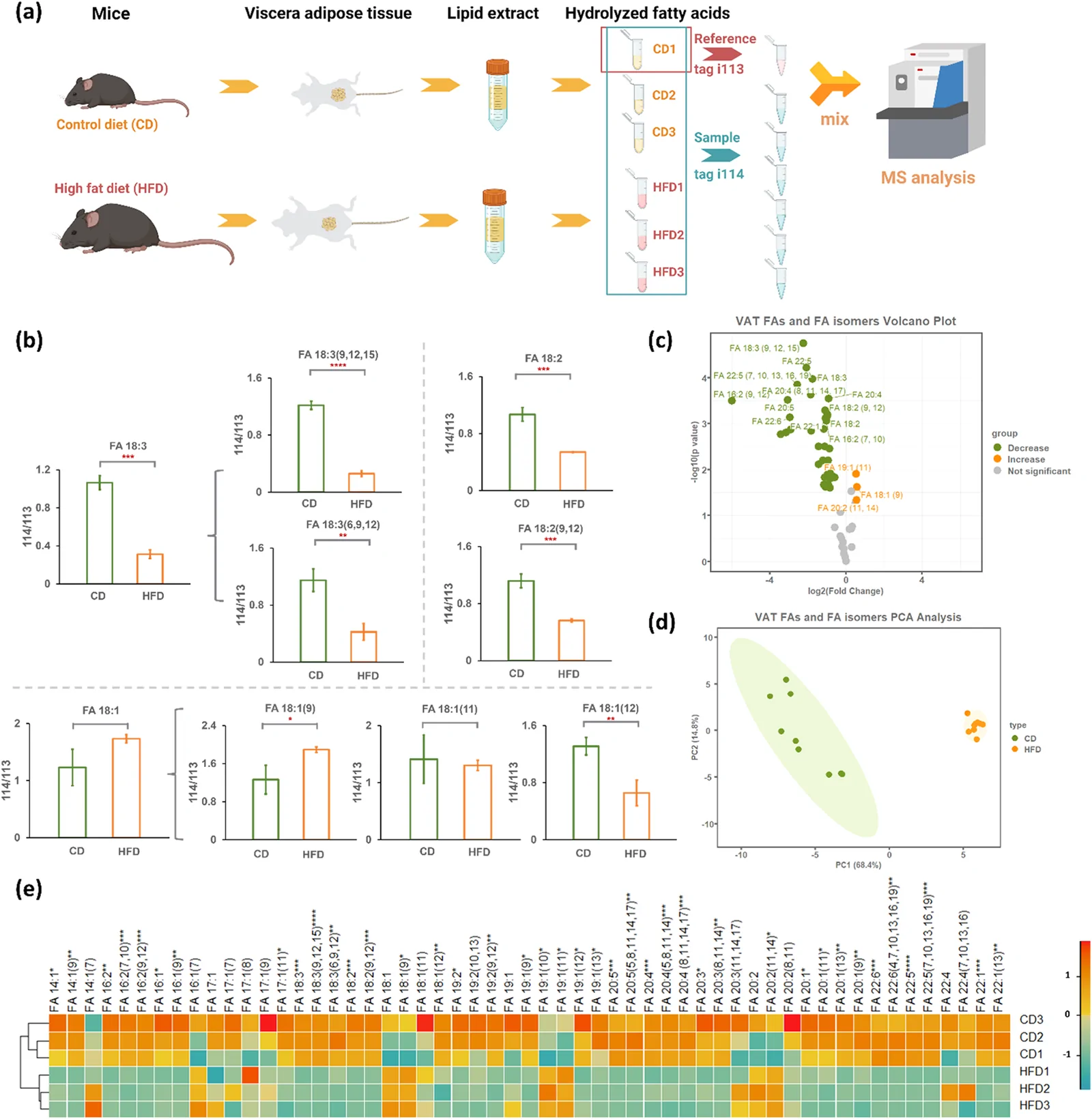
Duplex lipid isomeromics reveals diet-associated FA isomer remodeling in murine visceral adipose tissue (a) Workflow for analysis of FAs released by saponification of lipids extracted from VAT of mice fed CD or HFD for 14 weeks. Hydrolyzed FAs were derivatized with duplex AMPE-tags and analyzed by LC–MS/MS for C=C positional-isomer identification and total- and isomer-level relative quantification. (b) Representative total-level and isomer-level quantification results for FA 18:1, FA 18:2, FA 18:3, and their C=C positional isomers, expressed as HFD/CD abundance ratios. Divergent changes among FA 18:1 positional isomers illustrate isomer masking in total-level analysis. (c) Volcano plot of the 55 total FAs and FA positional isomers quantified in VAT. Log_2_ fold change was calculated as log_2_([FA]_HFD_/[FA]_CD_). Species with log_2_ fold change >0.4 or < −0.4 and *p* < 0.05 were classified as increased or decreased, respectively. (d) Principal component analysis of the combined FA and FA-isomer profiles, showing separation between CD and HFD groups. (e) Hierarchical clustering analysis of the quantified FA and FA-isomer profiles. Statistical significance was assessed from biological replicates and is indicated as *p* < 0.05 (*), *p* < 0.01 (**), *p* < 0.001 (***), and *p* < 0.0001 (****).

Across the VAT samples, 18 unsaturated FAs and 37 corresponding C=C positional isomers were identified and relatively quantified (Table S4). Principal component analysis showed clear separation of the CD and HFD groups based on the combined FA and FA-isomer profiles (Figure 5d), consistent with extensive diet-associated remodeling of the FA pool.^80–82^ Volcano-plot analysis further indicated that most detected unsaturated FAs and positional isomers were decreased in HFD samples relative to CD controls, with several notable exceptions, including FA 18:1(9), FA 19:1(11), and FA 20:2­(11,14) (Figure 5c).

Importantly, isomer-level analysis revealed changes that were obscured or attenuated when the same lipids were considered only at the total level. For example, total FA 18:1 did not differ significantly between the CD and HFD groups; however, its individual positional isomers exhibited divergent trends. FA 18:1(9) increased significantly in HFD VAT, FA 18:1(11) remained comparatively unchanged, and FA 18:1(12) decreased significantly (Figure 5b, see Supporting Information S11.5, for additional details on FA 18:1 positional-isomer assignment). Thus, the aggregate measurement of FA 18:1 masked opposing changes among its constituent isomers. A related pattern was observed for FA 18:3: although the total FA abundance differed between diet groups, FA 18:3­(9,12,15) displayed an even stronger diet-associated change. In contrast, the total-level and isomer-level responses of FA 18:2 were nearly identical, consistent with FA 18:2­(9,12) representing the dominant FA 18:2 isomer in these samples.

Polyunsaturated FAs exhibited some of the largest decreases in HFD VAT. In particular, FA 20:5, FA 22:5, and FA 22:6 each showed log_2_ fold changes below −2, suggesting an overall reduction in the relative abundance of highly unsaturated FA species in the HFD group. This trend is consistent with prior observations of increased FA saturation and reduced polyunsaturated FA content in tissues from HFD-fed mice.^83,84^ The analysis also enabled detection of several unsaturated odd-chain FAs, including FA 17:1, FA 19:1, and FA 19:2, together with their positional isomers. Although their diet-associated changes were generally less pronounced than those of even-chain FAs, these measurements illustrate the additional compositional detail accessible through isomer-resolved analysis.

These results demonstrate that duplex lipid isomeromics can reveal diet-associated FA remodeling that is not fully captured by conventional total-level measurements. The divergent behavior of FA 18:1 positional isomers illustrates the value of coupling structural resolution with quantitative analysis and identifies candidate isomer-level signatures for further investigation in larger biological cohorts.

## Conclusion

We developed AMPE-tags as a new class of duplex PB-derived isobaric reagents for one-step lipid derivatization, C=C positional-isomer identification, and relative quantification at both sum and isomer levels. The design integrates a PB-reactive ketone, an isotope-encoded reporter, and a mass-balancing group within a single reagent. Photoinduced PB reaction installs the reporter-bearing tag directly at lipid C=C bonds, enabling CID generation of position-specific diagnostic ions and subsequent HCD release of reporter ions from either intact PB products or isolated diagnostic ions. This fragmentation hierarchy provides complementary quantitative information: MS^2^ reporter ions measure the combined abundance of lipid positional isomers, whereas MS^3^ reporter ions generated from diagnostic ions measure individual C=C positional isomers.

Using the duplex AMPE-tag pair, we demonstrated accurate relative quantification of unsaturated FAs across a broad concentration range and in structurally diverse mixtures containing PB regioisomers and minor photochemical byproducts. Application to murine visceral adipose tissue revealed diet-associated remodeling of FA C=C positional isomers, including divergent changes among FA 18:1 isomers that were not apparent from total-level analysis alone. These results illustrate how duplex lipid isomeromics can uncover structural changes obscured in conventional sum-composition lipid measurements.

The current implementation is optimized most extensively for FAs, for which PB products generate abundant diagnostic ions. From a practical perspective, although AMPE-tags are structurally more complex than simple PB reagents such as 2-acetylpyridine, they can be prepared from accessible building blocks through a short synthetic route, with detailed procedures provided in the Supporting Information S1 and S2. The full hierarchical workflow requires MS^3^ capability for isomer-level reporter-ion quantification, and such capability is available on ion trap-Orbitrap and related hybrid and tribrid platforms commonly used in advanced lipidomics and proteomics facilities. On instruments without MS^3^, AMPE-tags can still support C=C positional-isomer identification and sum-level reporter-ion quantification by MS^2^.

Future studies will extend this PB-derived isobaric-reagent platform to intact lipid-class-resolved isomeromics, including glycerophospholipids, cholesteryl esters, triacylglycerols, and other unsaturated lipid classes. Such applications will require class-specific optimization of derivatization, separation, and fragmentation conditions, particularly for lipids in which headgroup or neutral-loss pathways compete with PB diagnostic-ion formation. More broadly, this work establishes PB-derived isobaric tagging as a chemical strategy for converting lipid C=C bonds into sites of both structural assignment and quantitative measurement.

## Supplementary Material



## Notes

Experimental details, materials and methods; synthesis and characterization of AMPE-tags, including ^1^H NMR spectra for tags; optimization of PB reaction and tandem MS conditions; investigation of PB reaction between tags and lipid from various categories; evaluation of tag reactivity; additional validation of total- and isomer-level quantification; limit of detection; additional information on analysis of an artificial fatty acid mixture; additional information on structural and quantitative analysis of fatty acids and fatty acid isomers in murine visceral adipose tissue; correspondence between Δ-position and n-position nomenclature (PDF)

## Abbreviations

C=C: carbon–carbon double bond
PB reaction: Paternò–Büchi reaction
AMPE: 1-(4-(aminomethyl)­phenyl)­ethan-1-one
MS: mass spectrometry
HPLC: high-performance liquid chromatography
CID: collision-induced dissociation
HCD: higher-energy collisional dissociation
FA: fatty acid
GPL: glycerophospholipid
CE: cholesteryl ester
TAG: triacylglycerol
TMT: tandem mass tag
iTRAQ: isobaric tags for relative and absolute quantification
VAT: visceral adipose tissue
CD: chow diet
HFD: high-fat diet

## References

[1] Brügger B. (2014). Lipidomics:
Analysis of the lipid composition of cells and subcellular organelles
by electrospray ionization mass spectrometry. Annu. Rev. Biochem..

[2] Züllig T., Trötzmüller M., Köfeler H. C. (2020). Lipidomics
from sample preparation to data analysis: A primer. Anal. Bioanal. Chem..

[3] Han X. (2016). Lipidomics
for studying metabolism. Nat. Rev. Endocrinol..

[4] Paine M. R. L., Poad B. L. J., Eijkel G. B., Marshall D. L., Blanksby S. J., Heeren R. M. A., Ellis S. R. (2018). Mass spectrometry imaging with isomeric
resolution enabled by ozone-induced dissociation. Angew. Chem., Int. Ed..

[5] Tang F., Guo C., Ma X., Zhang J., Su Y., Tian R., Shi R., Xia Y., Wang X., Ouyang Z. (2018). Rapid in situ profiling
of lipid C=C location isomers in tissue using ambient mass
spectrometry with photochemical reactions. Anal.
Chem..

[6] Zhang W., Zhang D., Chen Q., Wu J., Ouyang Z., Xia Y. (2019). Online photochemical derivatization
enables comprehensive mass spectrometric
analysis of unsaturated phospholipid isomers. Nat. Commun..

[7] Kuo T.-H., Chung H.-H., Chang H.-Y., Lin C.-W., Wang M.-Y., Shen T.-L., Hsu C.-C. (2019). Deep lipidomics
and molecular imaging
of unsaturated lipid isomers: A universal strategy initiated by mcpba
epoxidation. Anal. Chem..

[8] Cao W., Cheng S., Yang J., Feng J., Zhang W., Li Z., Chen Q., Xia Y., Ouyang Z., Ma X. (2020). Large-scale
lipid analysis with C=C location and *sn*-position
isomer resolving power. Nat. Commun..

[9] Zhang H., Xu M., Shi X., Liu Y., Li Z., Jagodinsky J. C., Ma M., Welham N. V., Morris Z. S., Li L. (2021). Quantification and
molecular imaging of fatty acid isomers from complex biological samples
by mass spectrometry. Chem. Sci..

[10] Blanksby S.
J., Mitchell T. W. (2010). Advances
in mass spectrometry for lipidomics. Annu. Rev.
Anal. Chem..

[11] Li L., Han J., Wang Z., Liu J., Wei J., Xiong S., Zhao Z. (2014). Mass spectrometry methodology
in lipid analysis. Int. J. Mol. Sci..

[12] Xu T., Hu C., Xuan Q., Xu G. (2020). Recent advances in analytical strategies
for mass spectrometry-based lipidomics. Anal.
Chim. Acta.

[13] Züllig T., Köfeler H. C. (2021). High resolution mass spectrometry in lipidomics. Mass Spectrom. Rev..

[14] Zhang W., Jian R., Zhao J., Liu Y., Xia Y. (2022). Deep-lipidotyping
by mass spectrometry: Recent technical advances and applications. J. Lipid Res..

[15] Ekroos K., Ejsing C. S., Bahr U., Karas M., Simons K., Shevchenko A. (2003). Charting molecular
composition of phosphatidylcholines
by fatty acid scanning and ion trap MS^3^ fragmentation. J. Lipid Res..

[16] Hsu F.-F., Turk J. (2008). Structural characterization
of unsaturated glycerophospholipids by
multiple-stage linear ion-trap mass spectrometry with electrospray
ionization. J. Am. Soc. Mass Spectrom..

[17] Campbell J. L., Baba T. (2015). Near-complete structural characterization of phosphatidylcholines
using electron impact excitation of ions from organics. Anal. Chem..

[18] Poad B. L. J., Pham H. T., Thomas M. C., Nealon J. R., Campbell J. L., Mitchell T. W., Blanksby S. J. (2010). Ozone-induced
dissociation on a modified
tandem linear ion-trap: Observations of different reactivity for isomeric
lipids. J. Am. Soc. Mass Spectrom..

[19] Pham H. T., Maccarone A. T., Campbell J. L., Mitchell T. W., Blanksby S. J. (2013). Ozone-induced
dissociation of conjugated lipids reveals significant reaction rate
enhancements and characteristic odd-electron product ions. J. Am. Soc. Mass Spectrom..

[20] O’Brien J. P., Needham B. D., Henderson J. C., Nowicki E. M., Trent M. S., Brodbelt J. S. (2014). 193 nm ultraviolet
photodissociation mass spectrometry
for the structural elucidation of Lipid A compounds in complex mixtures. Anal. Chem..

[21] Williams P. E., Klein D. R., Greer S. M., Brodbelt J. S. (2017). Pinpointing double
bond and *sn*-positions in glycerophospholipids via
hybrid 193 nm ultraviolet photodissociation (UVPD) mass spectrometry. J. Am. Chem. Soc..

[22] Brodbelt J. S., Morrison L. J., Santos I. (2020). Ultraviolet
photodissociation mass
spectrometry for analysis of biological molecules. Chem. Rev..

[23] Thomas M. C., Mitchell T. W., Blanksby S. J. (2006). Ozonolysis
of phospholipid double
bonds during electrospray ionization: A new tool for structure determination. J. Am. Chem. Soc..

[24] Thomas M. C., Mitchell T. W., Harman D. G., Deeley J. M., Murphy R. C., Blanksby S. J. (2007). Elucidation of double
bond position in unsaturated
lipids by ozone electrospray ionization mass spectrometry. Anal. Chem..

[25] Sun C., Zhao Y.-Y., Curtis J. M. (2013). The direct
determination of double
bond positions in lipid mixtures by liquid chromatography/in-line
ozonolysis/mass spectrometry. Anal. Chim. Acta.

[26] Poad B. L. J., Zheng X., Mitchell T. W., Smith R. D., Baker E. S., Blanksby S. J. (2018). Online ozonolysis
combined with ion mobility-mass spectrometry
provides a new platform for lipid isomer analyses. Anal. Chem..

[27] Feng Y., Chen B., Yu Q., Li L. (2019). Identification of double
bond position isomers in unsaturated lipids by m-CPBA epoxidation
and mass spectrometry fragmentation. Anal. Chem..

[28] Cao W., Ma X., Li Z., Zhou X., Ouyang Z. (2018). Locating carbon–carbon
double bonds in unsaturated phospholipids by epoxidation reaction
and tandem mass spectrometry. Anal. Chem..

[29] Tang S., Cheng H., Yan X. (2020). On-demand electrochemical epoxidation
in nano-electrospray ionization mass spectrometry to locate carbon–carbon
double bonds. Angew. Chem., Int. Ed..

[30] Tang S., Fan L., Cheng H., Yan X. (2021). Incorporating electro-epoxidation
into electrospray ionization mass spectrometry for simultaneous analysis
of negatively and positively charged unsaturated glycerophospholipids. J. Am. Soc. Mass Spectrom..

[31] Chintalapudi K., Badu-Tawiah A. K. (2020). An integrated
electrocatalytic nESI-MS platform for
quantification of fatty acid isomers directly from untreated biofluids. Chem. Sci..

[32] Wan L., Gong G., Liang H., Huang G. (2019). In situ analysis of
unsaturated fatty acids in human serum by negative-ion paper spray
mass spectrometry. Anal. Chim. Acta.

[33] Hirtzel E., Edwards M., Freitas D., Liu Z., Wang F., Yan X. (2023). Aziridination-assisted mass spectrometry
of nonpolar sterol lipids
with isomeric resolution. J. Am. Soc. Mass Spectrom..

[34] Wan Q., Xiao Y., Feng G., Dong X., Nie W., Gao M., Meng Q., Chen S. (2024). Visible-light-activated aziridination
reaction enables simultaneous resolving of C=C bond location
and the *sn*-position isomers in lipids. Chin. Chem. Lett..

[35] Zhang B., Wang Y., Zhou B.-W., Cheng J., Xu Q., Zhang L., Sun T.-q., Zhang J., Guo Y.-l. (2022). Chloramine-T-enabled
mass spectrometric analysis of C=C isomers of unsaturated fatty
acids and phosphatidylcholines in human thyroids. Anal. Chem..

[36] Yang T., Tang S., Kuo S.-T., Freitas D., Edwards M., Wang H., Sun Y., Yan X. (2022). Lipid mass
tags via
aziridination for probing unsaturated lipid isomers and accurate relative
quantification. Angew. Chem., Int. Ed..

[37] Feng G., Gao M., Chen H., Zhang Z., Chen J., Tong Y., Wu P., Fu R., Lin Y., Chen S. (2024). Stable-isotope N-Me
aziridination enables accurate quantitative C=C isomeric lipidomics. Anal. Chem..

[38] Minnikin D. E. (1978). Location
of double bonds and cyclopropane rings in fatty acids by mass spectrometry. Chem. Phys. Lipids.

[39] Francis G. W. (1981). Alkylthiolation
for the determination of double-bond position in unsaturated fatty
acid esters. Chem. Phys. Lipids.

[40] Kwon Y., Lee S., Oh D.-C., Kim S. (2011). Simple determination of double-bond
positions in long-chain olefins by cross-metathesis. Angew. Chem., Int. Ed..

[41] Unsihuay D., Su P., Hu H., Qiu J., Kuang S., Li Y., Sun X., Dey S. K., Laskin J. (2021). Imaging and analysis of isomeric
unsaturated lipids through online photochemical derivatization of
carbon–carbon double bonds. Angew. Chem.,
Int. Ed..

[42] Ma X., Xia Y. (2014). Pinpointing double bonds in lipids by Paternò-Büchi
reactions and mass spectrometry. Angew. Chem.,
Int. Ed..

[43] Ma X., Chong L., Tian R., Shi R., Hu T. Y., Ouyang Z., Xia Y. (2016). Identification and quantitation of
lipid C=C location isomers: A shotgun lipidomics approach enabled
by photochemical reaction. Proc. Natl. Acad.
Sci. U.S.A..

[44] D’Auria M. (2019). The Paternò–Büchi
reaction – a comprehensive review. Photochem.
Photobiol. Sci..

[45] Fréneau M., Hoffmann N. (2017). The Paternò-Büchi
reaction—mechanisms
and application to organic synthesis. J. Photochem.
Photobiol., C.

[46] Stinson C. A., Xia Y. (2016). A method of coupling the Paternò–Büchi reaction
with direct infusion ESI-MS/MS for locating the C=C bond in
glycerophospholipids. Analyst.

[47] Xie X., Xia Y. (2019). Analysis of conjugated
fatty acid isomers by the Paternò-Büchi
reaction and trapped ion mobility mass spectrometry. Anal. Chem..

[48] Xie X., Zhao J., Lin M., Zhang J.-L., Xia Y. (2020). Profiling
of cholesteryl esters by coupling charge-tagging Paternò–Büchi
reaction and liquid chromatography–mass spectrometry. Anal. Chem..

[49] Franklin E. T., Xia Y. (2020). Structural elucidation
of triacylglycerol using online acetone Paternò–Büchi
reaction coupled with reversed-phase liquid chromatography mass spectrometry. Analyst.

[50] Bednařík A., Bölsker S., Soltwisch J., Dreisewerd K. (2018). An on-tissue
Paternò–Büchi reaction for localization of carbon–carbon
double bonds in phospholipids and glycolipids by matrix-assisted laser-desorption–ionization
mass-spectrometry imaging. Angew. Chem., Int.
Ed..

[51] Wäldchen F., Spengler B., Heiles S. (2019). Reactive matrix-assisted laser desorption/ionization
mass spectrometry imaging using an intrinsically photoreactive Paternò–Büchi
matrix for double-bond localization in isomeric phospholipids. J. Am. Chem. Soc..

[52] Zhang J., Guo C., Huo X., Ma X., Li X., Abliz Z., Chu Y., Wang X., Tang F. (2021). Unsaturated lipid isomeric imaging
based on the Paternò–Büchi reaction in the solid
phase in ambient conditions. Talanta.

[53] Rustam Y. H., Reid G. E. (2018). Analytical challenges
and recent advances in mass spectrometry
based lipidomics. Anal. Chem..

[54] Wang M., Wang C., Han X. (2017). Selection
of internal standards for
accurate quantification of complex lipid species in biological extracts
by electrospray ionization mass spectrometry—what, how and
why?. Mass Spectrom. Rev..

[55] Oda Y., Huang K., Cross F. R., Cowburn D., Chait B. T. (1999). Accurate
quantitation of protein expression and site-specific phosphorylation. Proc. Natl. Acad. Sci. U.S.A..

[56] Gygi S. P., Rist B., Gerber S. A., Turecek F., Gelb M. H., Aebersold R. (1999). Quantitative
analysis of complex protein mixtures using
isotope-coded affinity tags. Nat. Biotechnol..

[57] Thompson A., Schäfer J., Kuhn K., Kienle S., Schwarz J., Schmidt G., Neumann T., Hamon C. (2003). Tandem mass tags: A
novel quantification strategy for comparative analysis of complex
protein mixtures by MS/MS. Anal. Chem..

[58] Ross P. L., Huang Y. N., Marchese J. N., Williamson B., Parker K., Hattan S., Khainovski N., Pillai S., Dey S., Daniels S. (2004). Multiplexed
protein quantitation in *S*
*accharomyces*
*cerevisiae* using amine-reactive isobaric tagging
reagents. Mol. Cell. Proteomics.

[59] Rauniyar N., Yates J. R. (2014). III. Isobaric labeling-based relative quantification
in shotgun proteomics. J. Proteome Res..

[60] Tokuoka S.
M., Kita Y., Sato M., Shimizu T., Yatomi Y., Oda Y. (2021). Development
of tandem mass tag labeling method for lipid molecules
containing carboxy and phosphate groups, and their stability in human
serum. Metabolites.

[61] Sun F., Choi A. A., Wu R. (2018). Systematic
analysis of fatty acids
in human cells with a multiplexed isobaric tag (TMT)-based method. J. Proteome Res..

[62] Tokuoka S. M., Kita Y., Shimizu T., Oda Y. (2019). Isobaric mass tagging
and triple quadrupole mass spectrometry to determine lipid biomarker
candidates for Alzheimer’s disease. PLoS
One.

[63] Zemski
Berry K. A., Murphy R. C. (2006). Analysis of polyunsaturated aminophospholipid
molecular species using isotope-tagged derivatives and tandem mass
spectrometry/mass spectrometry/mass spectrometry. Anal. Biochem..

[64] Armbruster M. R., Mostafa M. E., Caldwell R. N., Grady S. F., Arnatt C. K., Edwards J. L. (2023). Isobaric 6-plex and tosyl dual tagging
for the determination
of positional isomers and quantitation of monounsaturated fatty acids
using rapid UHPLC-MS/MS. Analyst.

[65] Armbruster M. R., Grady S. F., Arnatt C. K., Edwards J. L. (2022). Isobaric 4-plex
tagging for absolute quantitation of biological acids in diabetic
urine using capillary LC–MS/MS. ACS Measurement
Science Au.

[66] Gu T.-J., Feng Y., Wang D., Li L. (2022). Simultaneous multiplexed
quantification and C=C localization of fatty acids with LC-MS/MS
using isobaric multiplex reagents for carbonyl-containing compound
(SUGAR) tags and C=C epoxidation. Anal.
Chim. Acta.

[67] Feng Y., Lv Y., Gu T.-J., Chen B., Li L. (2022). Quantitative analysis
and structural elucidation of fatty acids by isobaric multiplex labeling
reagents for carbonyl-containing compound (SUGAR) tags and m-CPBA
epoxidation. Anal. Chem..

[68] Gu T.-J., Liu P.-K., Wang Y.-W., Flowers M. T., Xu S., Liu Y., Davis D. B., Li L. (2024). Diazobutanone-assisted isobaric labelling
of phospholipids and sulfated glycolipids enables multiplexed quantitative
lipidomics using tandem mass spectrometry. Nat.
Chem..

[69] Zhao J., Xie X., Lin Q., Ma X., Su P., Xia Y. (2020). Next-generation
Paternò–Büchi reagents for lipid analysis by
mass spectrometry. Anal. Chem..

[70] Sniderman A. D., Cianflone K., Arner P., Summers L. K. M., Frayn K. N. (1998). The adipocyte,
fatty acid trapping, and atherogenesis. Arterioscler.,
Thromb., Vasc. Biol..

[71] Luo L., Liu M. (2016). Adipose tissue in control of metabolism. J.
Endocrinol..

[72] Speakman J. R. (2019). Use of
high-fat diets to study rodent obesity as a model of human obesity. Int. J. Obes..

[73] Li J., Wu H., Liu Y., Yang L. (2020). High fat diet induced obesity model
using four strains of mice: Kunming, c57BL/6, BALB/c and ICR. Exp. Anim..

[74] Stott N. L., Marino J. S. (2020). High fat rodent models of type 2 diabetes: From rodent
to human. Nutrients.

[75] Echeverría F., Valenzuela R., Bustamante A., Álvarez D., Ortiz M., Espinosa A., Illesca P., Gonzalez-Mañan D., Videla L. A. (2019). High-fat diet induces mouse liver steatosis with a
concomitant decline in energy metabolism: Attenuation by eicosapentaenoic
acid (EPA) or hydroxytyrosol (HT) supplementation and the additive
effects upon EPA and HT co-administration. Food
Funct..

[76] Gallou-Kabani C., Vigé A., Gross M.-S., Rabès J.-P., Boileau C., Larue-Achagiotis C., Tomé D., Jais J.-P., Junien C. (2007). C57BL/6J and A/J mice fed a high-fat
diet delineate components of metabolic syndrome. Obesity.

[77] Hou B., Zhao Y., He P., Xu C., Ma P., Lam S. M., Li B., Gil V., Shui G., Qiang G. (2020). Targeted lipidomics and transcriptomics profiling reveal
the heterogeneity of visceral and subcutaneous white adipose tissue. Life Sci..

[78] Young R. S. E., Flakelar C. L., Narreddula V. R., Jekimovs L. J., Menzel J. P., Poad B. L. J., Blanksby S. J. (2022). Identification
of carbon-carbon double
bond stereochemistry in unsaturated fatty acids by charge-remote fragmentation
of fixed-charge derivatives. Anal. Chem..

[79] Xia T., Jin X., Zhang D., Wang J., Jian R., Yin H., Xia Y. (2023). Alternative fatty acid desaturation pathways revealed by deep profiling
of total fatty acids in RAW 264.7 cell line. J. Lipid Res..

[80] Yu H., Bi Y., Ma W., He L., Yuan L., Feng J., Xiao R. (2010). Long-term effects of high lipid and
high energy diet on serum lipid,
brain fatty acid composition, and memory and learning ability in mice. Int. J. Dev. Neurosci..

[81] Oosterveer M. H., van Dijk T. H., Tietge U. J. F., Boer T., Havinga R., Stellaard F., Groen A. K., Kuipers F., Reijngoud D.-J. (2009). High fat
feeding induces hepatic fatty acid elongation in mice. PLoS One.

[82] Pakiet A., Jakubiak A., Mierzejewska P., Zwara A., Liakh I., Sledzinski T., Mika A. (2020). The effect of a high-fat diet on
the fatty acid composition in the hearts of mice. Nutrients.

[83] Ampong I., Ikwuobe O. J., Brown J. E. P., Bailey C. J., Gao D., Gutierrez-Merino J., Griffiths H. R. (2022). Odd chain fatty acid metabolism in
mice after a high fat diet. Int. J. Biochem.
Cell Biol..

[84] Yew
Tan C., Virtue S., Murfitt S., Roberts L. D., Phua Y. H., Dale M., Griffin J. L., Tinahones F., Scherer P. E., Vidal-Puig A. (2015). Adipose tissue fatty acid chain length
and mono-unsaturation increases with obesity and insulin resistance. Sci. Rep..

