# Supplementary material for: Duplex Paternò–Büchi Isobaric Reagents for One-Step C=C-Resolved Lipid Isomer Quantification
Source: J Am Chem Soc. 2026 Sep 28;148(39):42508–20. doi: 10.1021/jacs.6c13813 (PMC13651533; doi:10.1021/jacs.6c13813)
Supplement: Supplementary file 1 [file ja6c13813_si_001.pdf]

Supporting Information

Duplex Paternò–Büchi Isobaric Reagents for One-Step C=C-Resolved Lipid Isomer Quantification

Xi Chen[a], Gopal Reddy Ramidi[a], Nitya Shree[b], Dallas Freitas[a], Syuan-Ting Kuo[a], Huiying Guo[a], Luchen Wuyang[a], Aidan Slagter[a], Peiyu Cai[a], Ashok Polu[a], Mahua Choudhury[b], Xin Yan*[a]

[a] Department of Chemistry, Texas A&M University, College Station, TX 77843, USA

[b] Department of Pharmaceutical Sciences, Texas A&M University, College Station, TX 77843, USA

KEYWORDS: Lipid Quantification, Lipid C=C Bond Positional Isomer, Isobaric Mass Tag, Paternò-Büchi Reaction, Mass Spectrometry

Table of Contents

S1. Experiments and methods

S2. Synthesis of AMPE-tag, AMPE-tag i113 and AMPE-tag i114

S3. Dissociation investigation of AMPE-tag

S4. Optimization of PB reaction with PC 18:1(9)/18:1(9)

S5. PB reaction between AMPE-tag and lipids from different categories

S6. Condition investigation for tagging FAs with the duplex AMPE-tags

S7. Tag reactivity investigation for accurate quantification

S8. Further validation of AMPE-tag quantification feasibility

S9. Limit of detection

S10. Artificial FA mixture analysis by direct infusion

S11. Structural and quantitative analysis of FAs and FA isomers in murine VAT

S12. Correspondence between Δ-position and n-position nomenclature

S1. Experiments and Methods

S1.1 Research Compliance

C57BL/6J mice were bred, and 6-week-old male mice were randomized into two groups. One group was on a chow diet (# 8604, Teklad™ Diet) and the other was on a high-fat diet (#D12492, Research Diets Inc.) regimen for 14 weeks. All the mice were housed at ~22 °C with *ad libitum* access to their respective diet and water throughout, following a 12:12 h light and dark cycle. Mice were sacrificed at 20 weeks of age and VAT was collected for lipid isomeromics analysis. All procedures were performed in compliance with relevant laws and institutional guidelines and have been approved by the Institutional Animal Care and Use Committee of the Texas A&M Health Science Center (IACUC 2024-0191).

S1.2 Nomenclature

Lipid nomenclature used here refers to the guidelines by Liebisch *et al*.[1] The positions of C=C bonds are indicated by the numbers in brackets in lipid names using the nomenclature system. For example, FA 18:1(9) represents a fatty acid composed of an unsaturated chain 18:1 with one C=C bond between carbon 9 and 10 from the carbonyl group. For GPLs and TAGs which involve the stereonumbering (*sn*) positions between headgroups and fatty acyl chains, the *sn* positions of fatty acyl chains are denoted by the symbol of "/" and the fatty acyl chains at *sn*-1 position are listed first, followed by the ones at *sn*-2 position and then those at *sn*-3 position. If *sn* positions are not determined, the sign "_" is used.

S1.3 Tag Synthesis

Tag synthesis protocol was referred to the previous procedures.[2-6] 1-methylpiperazine, ethyl 2-bromoacetate, hydrochloric acid, and triethylamine were purchased from Sigma-Aldrich (St. Louis, MO). Isotope-labeled reagent ethyl 2-bromoacetate-1-^{13}C and ethyl 2-bromoacetate-2-^{13}C were purchased from Cambridge Isotopes Laboratories (Tewksbury, MA). *N,N'*-disuccinimidyl carbonate and trifluoroacetic acid were purchased from Fisher Scientific (Pittsburgh, PA). 1-(4-(aminomethyl)phenyl) ethan-1-one·HCl was purchased from Combi-Blocks (San Diego, CA). HPLC grade solvents ethyl acetate, water, acetonitrile, and methanol, as well as ACS grade solvents ethanol and dichloromethane were purchased from Sigma-Aldrich (St. Louis, MO). LC-MS grade dimethyl sulfoxide was purchased from Fisher Scientific (Pittsburgh, PA). The synthesis route is provided in Supporting Information, Scheme S1. MS and NMR data are also available in the Supporting Information below.

S1.4 Synthesis of Ethyl 2-(4-Methylpiperazin-1-yl)acetate Isotopologues

To a solution of 1-methylpiperazine 1 (2.41 g, 24 mmol, 4 eq) in ethyl acetate (15 ml) at room temperature was added ethyl 2-bromoacetate or ethyl 2-bromoacetate-1-^{13}C/ethyl 2-bromoacetate-2-^{13}C (1 g, 6 mmol, 1 eq), dropwise. The immediate formation of a white solid was observed. The reaction mixture was then heated in an oil bath at 80 °C for 2 hr. After completion of the reaction (monitored by TLC), the mixture was cooled to room temperature, diluted with ethyl acetate (15 ml), and washed with saturated aqueous $NaHCO_3$ solution (5 ml). The organic layer was dried over anhydrous Na_2SO_4, evaporated under reduced pressure, and the residue was dried under high vacuum for 1 hour to yield ethyl 2-(4-methylpiperazin-1-yl)acetate or its isotopologues **3** (0.9 g, 80%) as an off-white oil. The crude product was analyzed by MS and ^{1}H NMR, and was directly used for the next step without further purification.

S1.5 Synthesis of 2-(4-Methylpiperazin-1-yl)acetic Acid Isotopologues

The crude ester was suspended in a 1:1 mixture of concentrated hydrochloric acid and water (12 mL). This mixture was refluxed for 3 h, cooled to 40 °C, and evaporated under reduced pressure to dryness. The white residue was redissolved in water (6 ml) and evaporated to remove residual acid followed by co-distillation with ethanol (10 ml). The solid was redissolved in ethanol (15 ml) and refluxed with a small amount of water (0.75 ml) added. The refluxing

mixture was allowed to cool slowly and refrigerated overnight to aid crystallization. Filtered the solid and washed the solid with ethanol (5 ml) and dried under high vacuum for 1 hour to yield 2-(4-methylpiperazin-1-yl) acetic acid or its isotopologues **4** (0.6 g, 79%) as a white solid.

S1.6 Synthesis of *N*-(4-acetylbenzyl)-2-(4-methylpiperazin-1-yl)acetamide Isotopologues

2-(4-Methylpiperazin-1-yl)acetic acid or its isotopologue **4** (0.2 g, 1.2 mmol, 1 eq) and triethylamine (0.19 g, 1.9 mmol, 1.5 eq) were dissolved in acetonitrile (6 ml) at rt and *N,N'*-disuccinimidyl carbonate **5** (0.39 g, 1.5 mmol, 1.2 eq) was added dropwise at rt. Then the reaction mixture was stirred for 2 h at room temperature. 1-(4-(aminomethyl)phenyl) ethan-1-one·HCl **7** (0.22 g, 1.2 mmol, 1.2 eq) was pre-suspended in acetonitrile (1.2 ml) with triethylamine (0.19 g, 1.9 mmol, 1.5 eq) and added to the above reaction mass at rt. The reaction mass was heated to 40 °C and maintained overnight. After completion of the reaction (monitored by TLC), the solvent was removed under vacuum below 40 °C and purified by column chromatography over silica gel (methanol/dichloromethane v/v, 1:9), which afforded the desired product **8** (0.15 g, 41%, over two steps) as an off-white solid. As 1-(4-(aminomethyl)phenyl) ethan-1-one, which provides the carbonyl group for photoreaction is abbreviated as AMPE here, the tag synthesized is referred to as AMPE-tag, and two isotopologues are distinguished as AMPE-tag i113 and AMPE-tag i114 due to the release of a mass reporter ion at *m/z* 113 or 114. Final tags were dissolved in dimethyl sulfoxide (DMSO) containing 5% trifluoroacetic acid at a concentration of 500 mM for further use.

S1.7 Photoreaction and Isobaric Tag Labeled Lipids Generation

Photoreaction was conducted with a micro-photoreactor described by Zhang et al.[7, 8] The photoreactor was home-built with a 3D printer. The scaffold of the photoreactor was composed of black ABS filament (Hatchbox, Pomona, CA) with two ends connected by stainless steel machined rods with a diameter of 2.3 mm. The photoreactor dimensions are 40 mm x 40 mm x 50 mm. A low-pressure mercury UV lamp at 254 nm (BHK, Ontario, CA) was inserted through the hole on one end of the photoreactor. Fluorinated ethylene propylene (FEP) tubing with 1/16" OD x 0.030" ID (Fisher Scientific, Pittsburgh, PA) was coiled concentrically around the stainless-steel rod with a length of 880 mm and exposed to the UV light at a distance of 2.5 cm, which provides a pathway for the reaction solution irradiated by UV light. The photoreactor is covered with aluminum foil to prevent any interference light from the environment. A fan is placed aside to cool down the temperature during the photoreaction. More information about the photoreactor can be found in SI.

Validation of methods was tested with lipid standards. GPL standards were purchased from Avanti Polar Lipids (Alabaster, AL); FA, CE, and TAG standards were purchased from Nu-Chek Prep (Elysian, MN). For lipid standards, 1-200 µM lipid was prepared in acetonitrile/dichloromethane 9/1 (v/v) and added with 2.5 mM AMPE-tag or the isotopologues. The mixture was purged with N_2 for 2 min. Then the solution was transferred to a syringe and injected into the micro-photoreactor at a flow rate of 0.4 mL/min by a syringe pump (Harvard Apparatus, Holliston, MA), with the UV lamp prewarmed for at least 15 min. The reaction time was around 1 min. A microcentrifuge tube was placed at the opening end of the FEP tubing to gather the reacted solution and the first 3 drops were discarded. To compare the lipid concentration in two samples, AMPE-tag i113/114 was used for each sample separately, and then the solutions were mixed with the same volume. The mixed solution can be used directly for MS analysis without any other preparation.

An artificial mixture of six FAs was diluted to 10 µM and 20 µM per FA. Tag 3.0 mM was added, and photoreaction was conducted as described above.

S1.8 Lipid Extraction from Mouse Visceral Adipose Tissue (VAT), Fatty Acid Hydrolysis and Tag Labeling

LC-MS grade formic acid and KOH were purchased from Fisher Scientific (Pittsburgh, PA). HPLC-grade solvents were purchased from Sigma-Aldrich (St. Louis, MO), except for isooctane, purchased from Fisher Scientific (Pittsburgh, PA).

The lipid extraction protocol was adapted from the modified Bligh & Dyer extraction procedure by Zacharia et al.[9] Mouse VAT (20 mg) was added to 800 µL ice-cold extraction solvent, composed of: MeOH/H_2O/formic acid 85/13/2 (v/v/v), and an internal standard of TAG 19:0/19:0/19:0 at a concentration of 0.1 mg/mL. Then the sample was ground with a homogenizer for 5 min, followed by vortexing for 30 s and sonication in ice-cold water for 15 min. The homogenate was added with 400 µL chloroform, and vortexed for 30 s. Water was added at the same volume as chloroform, and the mixture was vortexed again. Afterward, the sample was incubated on ice for 20 min, followed by another 15 min of sonication. Then it was centrifuged at 5000 rpm for 10 min. The hydrophilic upper layer was discarded, while the lipophilic lower phase was transferred to a clean glass vial. Solvent was removed under a N_2 stream, and the dry sample was kept at -80 °C until further steps.

The lipid extract was hydrolyzed to yield free FAs. The hydrolysis procedure was adapted from saponification procedure as previously described.[10-12] The dried lipid extract was redissolved in chloroform (1 mL). 40 µL solution was added to a 10 mL glass vial and then saponified with 2 mL acetonitrile/15% KOH 50/50 (v/v) at 85 °C for 1 h, and acetonitrile containing 2 M FA 15:0 as internal standard. 1 M HCl was added to acidify the solution and terminate hydrolysis. The hydrolyzed total FAs were extracted twice with 2 mL isooctane and sonicated for 5 minutes each time. The mixture was centrifuged at 4000 rpm for 5 min for phase separation. The organic layers were collected, and the solvent was evaporated under N_2. 1 mL of dichloromethane was added to dissolve all the extracted fatty acids.

Aforementioned hydrolyzed fatty acids were diluted 10 times with acetonitrile. AMPE-tag i113/i114 was added to the solution at a concentration of 10 mM. PB reaction was initiated using the same apparatus and protocol as described above.

S1.9 MS Analysis

All samples were analyzed with an LTQ XL linear ion trap mass spectrometer (Thermo Fisher Scientific, San Jose, CA) and an Orbitrap Velos Pro mass spectrometer (Thermo Fisher Scientific, San Jose, CA). Most analyses were conducted in positive ion mode. The following MS parameters were used for direct infusion acquisition with a homemade wire-in-a-capillary nanoelectrospray ionization (nanoESI) source: spray voltage 1.5 kV, S-lens RF level 67.9%, capillary temperature 150 °C. Full MS scans were acquired at *m/z* 200-1200 at a resolving power of 60000. A maximum injection time of 500 ms and 1 micro-scan were used for full MS scans. MS/MS and MS/MS/MS acquisitions were performed upon CID and HCD. The normalized CID energy was optimized to 40 arbitrary units (a.u.) and the normalized HCD energy was optimized to 45 a.u. in MS/MS and 40 in MS/MS/MS. In MS/MS, the isolation window width was set as 1.0; while in MS/MS/MS, the isolation window width was widened to 2.0. The auto gain control (AGC) was set as $1x10^6$ for full MS acquisition, $5x10^4$ for MS/MS acquisition, and $5x10^5$ for MS/MS/MS acquisition. For the HPLC-HESI-MS platform, the following parameters were used: spray voltage 3 kV, capillary temperature 350 °C, S-lens RF level 60%. The sheath gas, auxiliary gas, and sweep gas flow rate were 40, 10, and 1 arb, respectively; MS resolution was selected at 30000 for full MS and 15000 for tandem MS. CID, HCD energy, and isolation window width were the same as those used for direct infusion. AGC was set at $1x10^5$ for all tandem MS.

S1.10 Liquid Chromatography

LC-MS grade water, methanol, and acetonitrile were purchased from Sigma-Aldrich (St. Louis, MO). LC-MS grade formic acid, ammonium formate and isopropanol were purchased from Fisher Scientific (Pittsburgh, PA). A Vanquish UHPLC system (Thermo Fisher Scientific, San Jose, CA) was connected to the Orbitrap Velos Pro mass spectrometer via the commercial heated electrospray ionization (HESI) source for LC-MS analysis. Separation was performed on the Accucore C30 column (C30, 2.1 mm x 150 mm, 2.6 µm) (Thermo Fisher Scientific, San Jose, CA). The column temperature was set at 40 °C and the flow rate at 0.2 mL/min. For the separation of hydrolyzed fatty acids and tag-labeled fatty acids, the mobile phase consisted of A: water and B: methanol, both containing 0.1% formic acid. Gradient elution started with mobile phase A 90% and mobile phase B 10%, and changed as follows: 10% B at 0-4 min, 10-75% B at 4-10 min, 75% B at 10-13 min, 75-95% B at 13-32 min, 95% B at 32-35 min, 95%-10% B at 35-42

min and 10% B at 42-45 min. For the separation of lipid extract from VAT, mobile phase consisted of A: acetonitrile/water 6/4 (v/v) and B: acetonitrile/isopropanol 1/9 (v/v), both containing 0.1% formic acid and 10 mM ammonium formate. The elution gradient was as follows: 30−50% B at 0−3 min (to waste), 50−70% B at 3−7 min, 70−99% B at 7−12 min, 99% B at 12−20 min, 99-70% B at 20-25 min, 70-30% B at 25-28 min and 30% B at 28-30 min. All eluting solvents were degassed by sonicating for 20 min and filtered via the in-line filter. Samples were filtered with 0.22 µm filters before injection and an aliquot of 5 µL was injected for separation and analysis.

S1.11 ^{1}H NMR

^{1}H NMR spectra were obtained from a Bruker 400 MHz instrument. $CDCl_3$, MeOH-d_4 and DMSO-d_6 were used as solvents. Chemical shifts (δ) are expressed in ppm.

S1.12 Data Analysis

The relative intensities of the corresponding C=C diagnostic ions and mass reporter ions of lipids were read from .raw files from Xcalibur Qual Browser from Thermo Fisher Scientific and were used for analysis and quantification. Statistical analysis was done with Microsoft Excel 2016 and Origin 2022. R (version 4.1.3) was used for further data analysis and visualization, such as a volcano plot, principal component analysis (PCA) and hierarchical cluster analysis (HCA). The significant differences were determined using unpaired t tests with a p-value < 0.05 indicating significance.

S2. Synthesis of AMPE-tag, AMPE-tag i113 and AMPE-tag i114

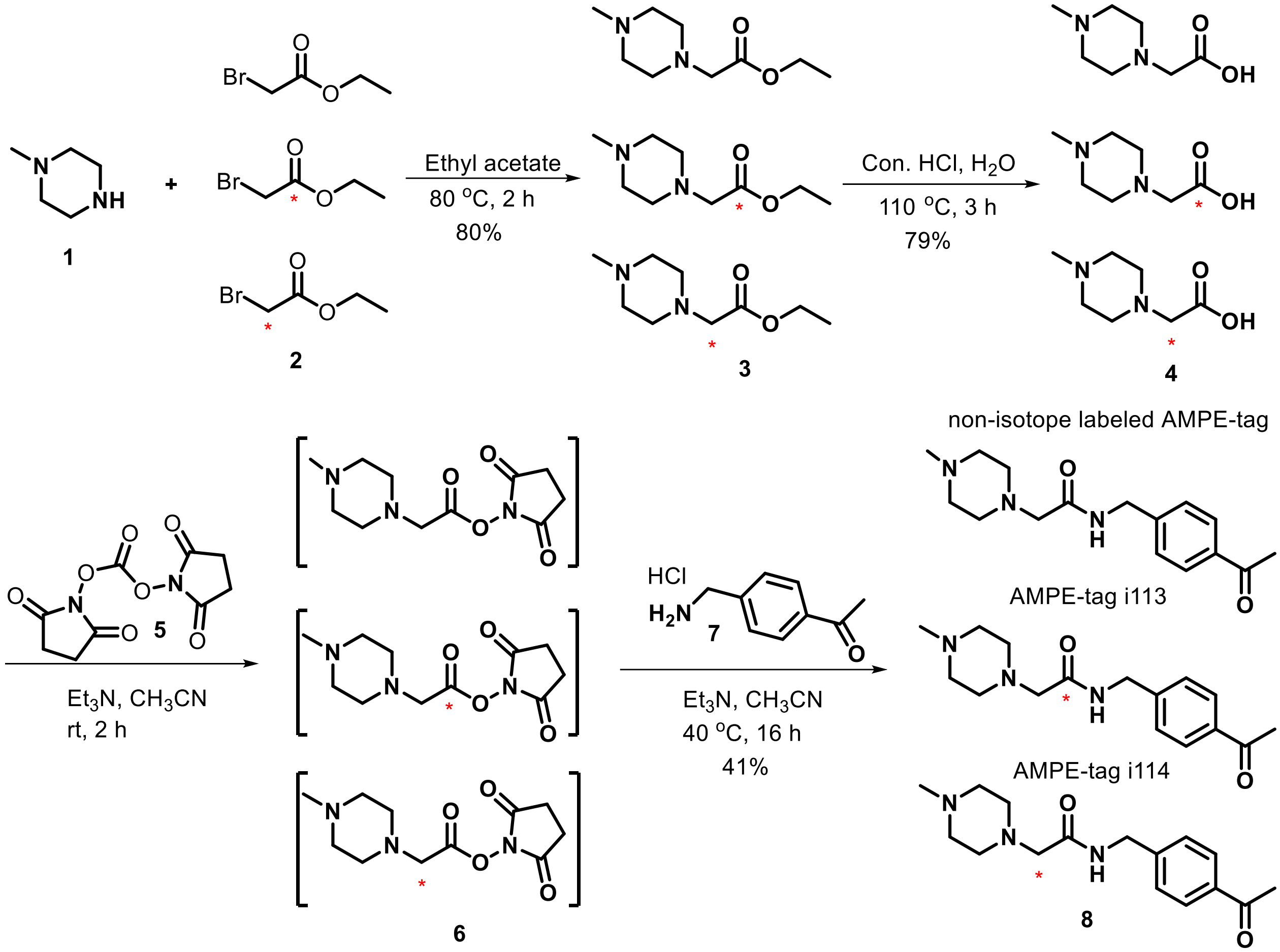

Scheme S1. Details of AMPE-tag and the isotopologues AMPE-tag i113 and AMPE-tag i114. The red asterisk represents ^{13}C.

MS and ^1H NMR data for non-isotope labeled AMPE-tag, AMPE-tag i113 and AMPE-tag i114. MS data here was acquired with an LTQ XL linear ion trap mass spectrometer from Thermo Fisher Scientific, and NMR data were acquired with a Bruker 400 MHz instrument.

S2.1 MS and ^1H NMR data of non-isotope labeled AMPE-tag

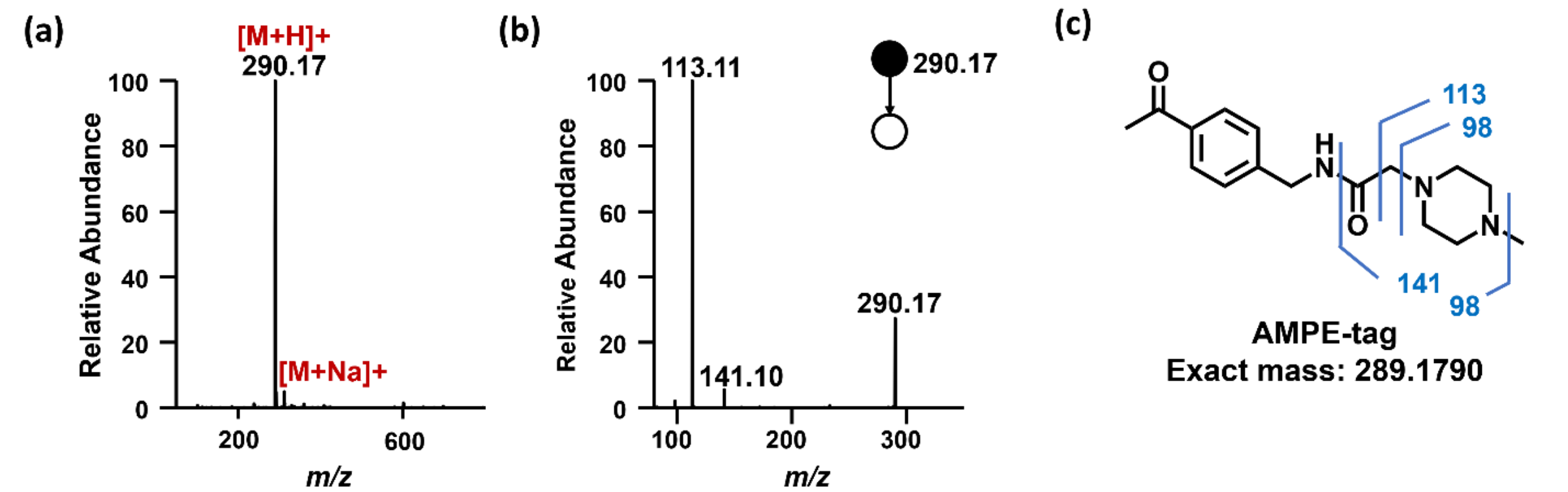

Figure S1. MS spectra of non-isotope labeled AMPE-tag in positive ion mode. (a) Full mass spectrum of AMPE-tag, which is found at *m/z* 290; (b) MS/MS spectrum of AMPE-tag, in which a mass reporter ion at *m/z* 113 is the major fragment upon CID; (c) the structure and fragments in tandem MS of AMPE-tag.

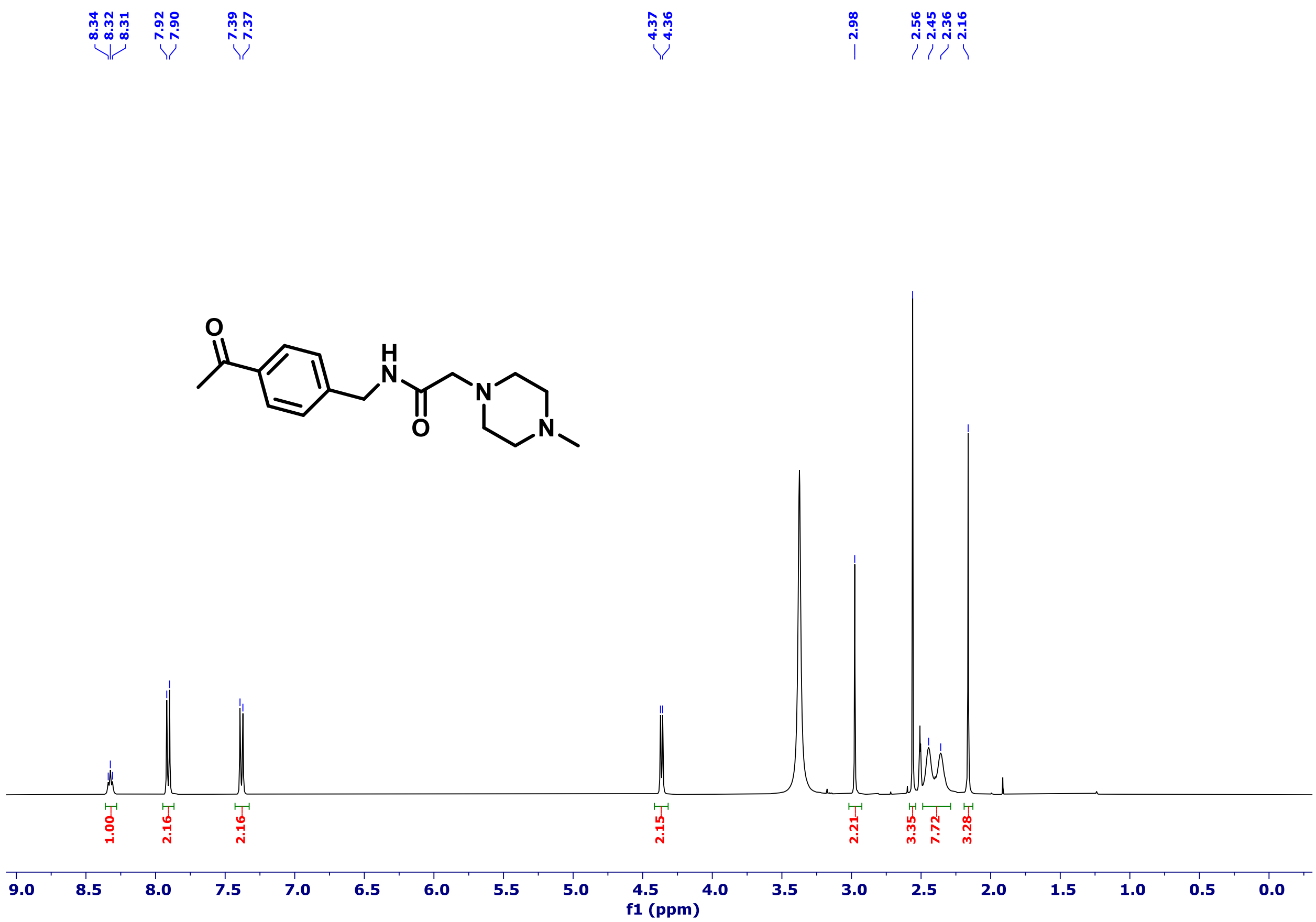

Figure S2. ^{1}H NMR spectrum of AMPE-tag (400 MHz, DMSO-d_6) δ 8.32 (t, *J* = 6.2 Hz, 1H), 7.91 (d, *J* = 8.3 Hz, 2H), 7.38 (d, *J* = 8.3 Hz, 2H), 4.36 (d, *J* = 6.3 Hz, 2H), 2.98 (s, 2H), 2.56 (s, 3H), 2.40 (m, 8H), 2.16 (s, 3H) ppm.

S2.2 MS and ^{1}H NMR data of AMPE-tag i113

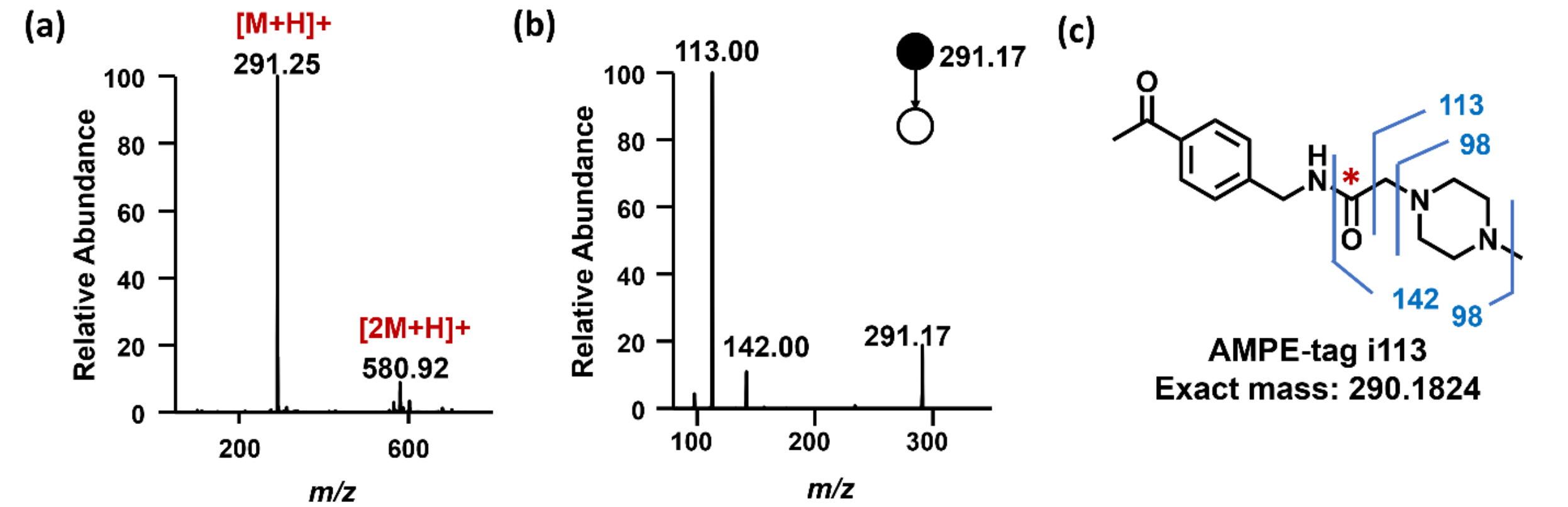

Figure S3. MS spectra of AMPE-tag i113 in positive ion mode. (a) Full mass spectrum of AMPE-tag i113, which is found at *m/z* 291; (b) MS/MS spectrum of AMPE-tag i113, in which a mass reporter ion at *m/z* 113 is the major fragment upon CID; (c) the structure and fragments in tandem MS of AMPE-tag i113.

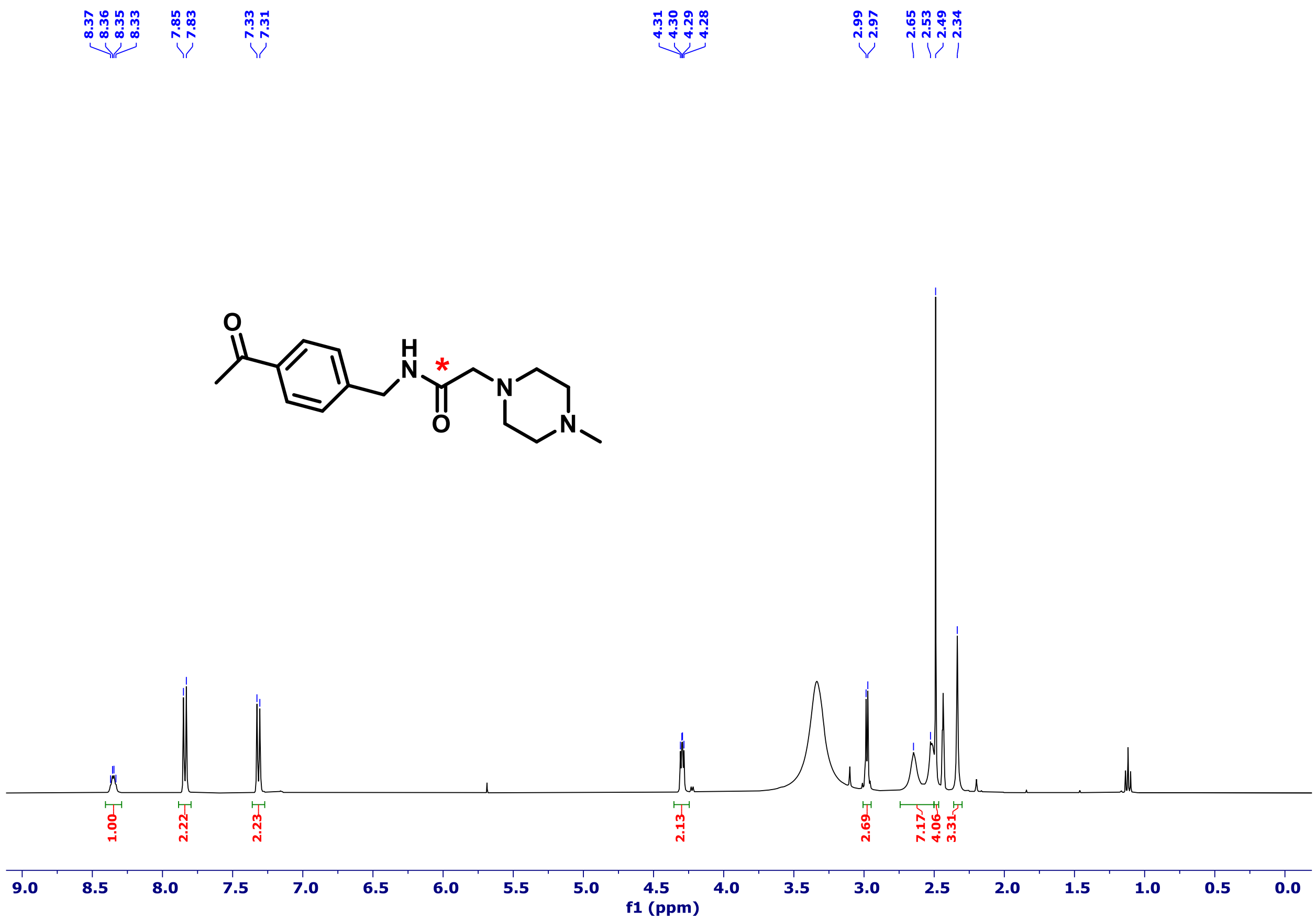

Figure S4. ^{1}H NMR spectrum of AMPE-tag i113 (400 MHz, DMSO-d_6) δ 8.35 (dd, J = 9.6, 5.6 Hz, 1H), 7.84 (d, J = 8.3 Hz, 2H), 7.32 (d, J = 8.2 Hz, 2H), 4.30 (dd, J = 5.9, 4.1 Hz, 2H), 2.98 (d, J = 5.0 Hz, 2H), 2.59 (m, 8H), 2.34 (s, 3H) ppm.

S2.3 MS and ^{1}H NMR data of AMPE-tag i114

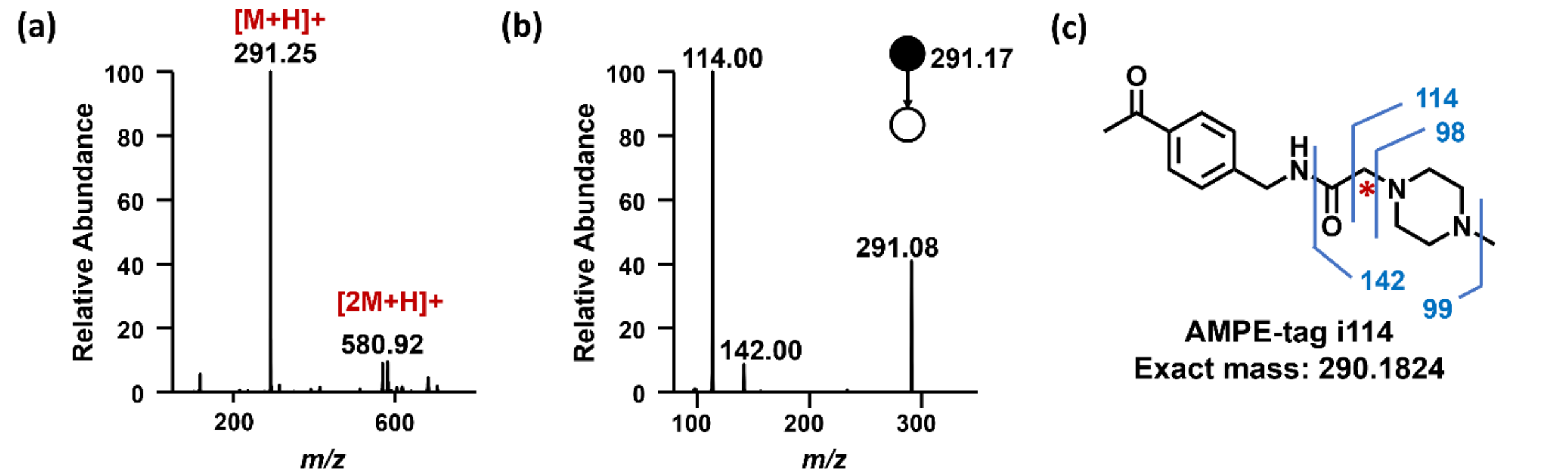

Figure S5. MS spectra of AMPE-tag i114 in positive ion mode. (a) Full mass spectrum of AMPE-tag i114, which is found at *m/z* 291; (b) MS/MS spectrum of AMPE-tag i114, in which a mass reporter ion at *m/z* 114 is the major fragment upon CID; (c) the structure and fragments in tandem MS of AMPE-tag i114.

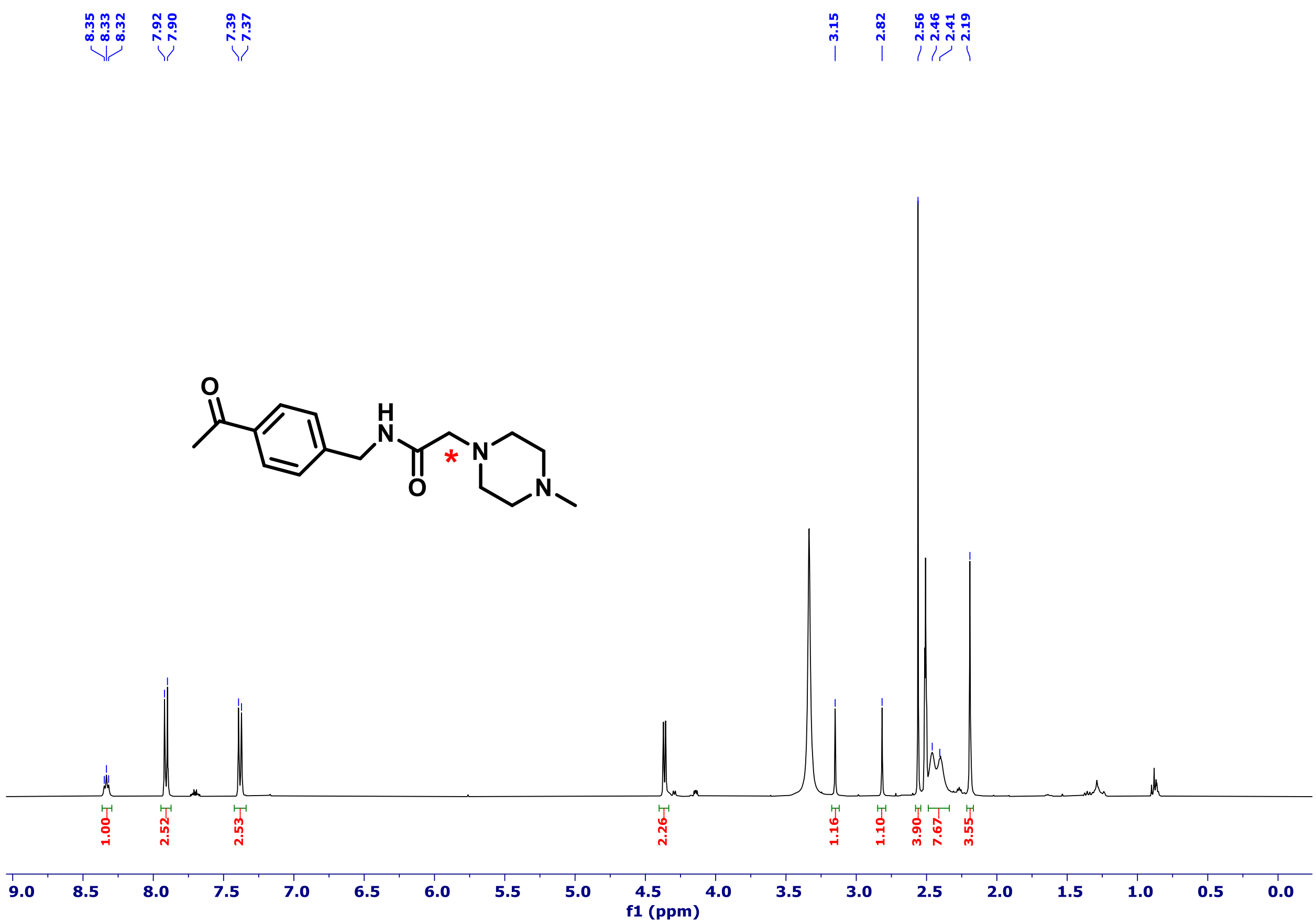

Figure S6. ^{1}H NMR spectrum of AMPE-tag i114 (400 MHz, DMSO-d_6): δ 8.33 (t, J = 6.1 Hz, 1H), 7.91 (m, 2H), 7.38 (d, J = 8.4 Hz, 2H), 4.36 (d, J = 6.3 Hz, 2H), 3.16 (s, 1H), 2.82 (s, 1H), 2.56 (s, 3H), 2.43 (m, 8H), 2.19 (s, 3H) ppm.

S3. Dissociation investigation of non-isotope labeled AMPE-tag

The AMPE-tag we designed and synthesized has high ionization efficiency and is susceptible to CID and HCD. Dissociation efficiency with collision energy (CE) was tested with the non-isotope labeled AMPE-tag. The precursor ion of AMPE-tag was isolated with a width of 1.0, and CID and HCD with different CE were applied to it. The result is shown in Figure S7. Upon CID, mass reporter ion is the dominant fragment, and its percentage among all fragments is stable after the CE reaches 30, when the AMPE-tag is completely dissociated. Increasing CE over 65 may cause the elimination of mass reporter ions due to further fragmentation. While upon HCD, more fragments are observed, as HCD requires a non-equilibrium process and is easy to cleave at various bond positions. But mass reporter ion is still the most abundant, with the highest percentage at CE around 45. Hence, optimized CE is chosen at 40 for CID and 45 for HCD.

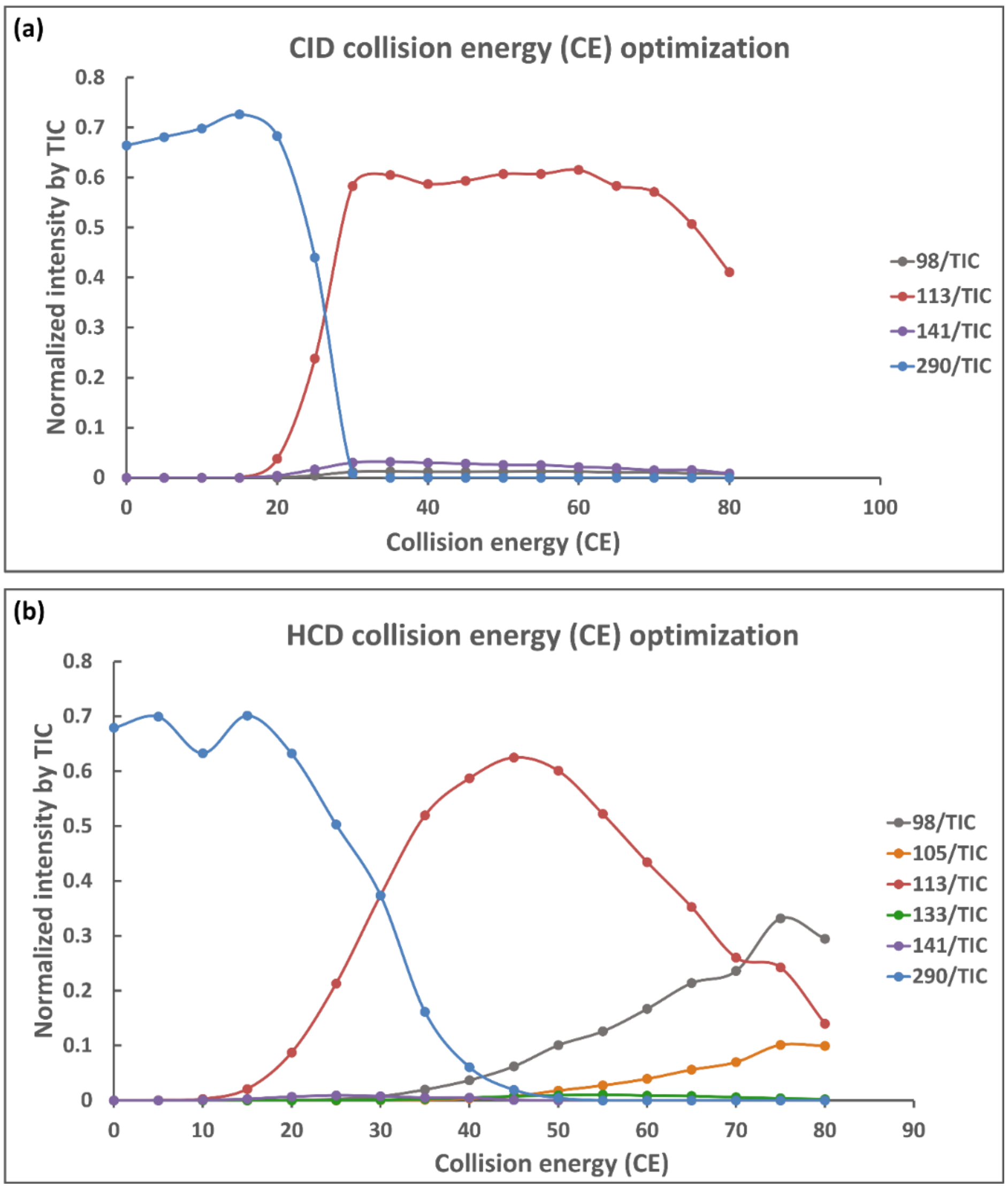

Figure S7. CE test and optimization of AMPE-tag upon (a) CID and (b) HCD.

It is very important that the dissociation of the duplex isobaric tags is identical and doesn't change with other factors. Total concentration of AMPE-tag i113 and i114 was maintained at 100 µM, while the concentration ratio of tag i113 to i114 ranged from 0.1, 1, 50, to 100. The intensity ratio of m/z 113 and 114 was plotted against the concentration ratio of tag i113 and i114, which resulted in a linear line with a slope close to 1 and an intercept close to 0 upon both

CID and HCD (Figure S8). The result indicated that AMPE-tag i113 and i114 have identical dissociation efficiency even if their concentrations are distinct. The mass reporter ion intensity is directly related to the relative abundance of the tag, which is the foundation of using the isobaric tag for quantification.

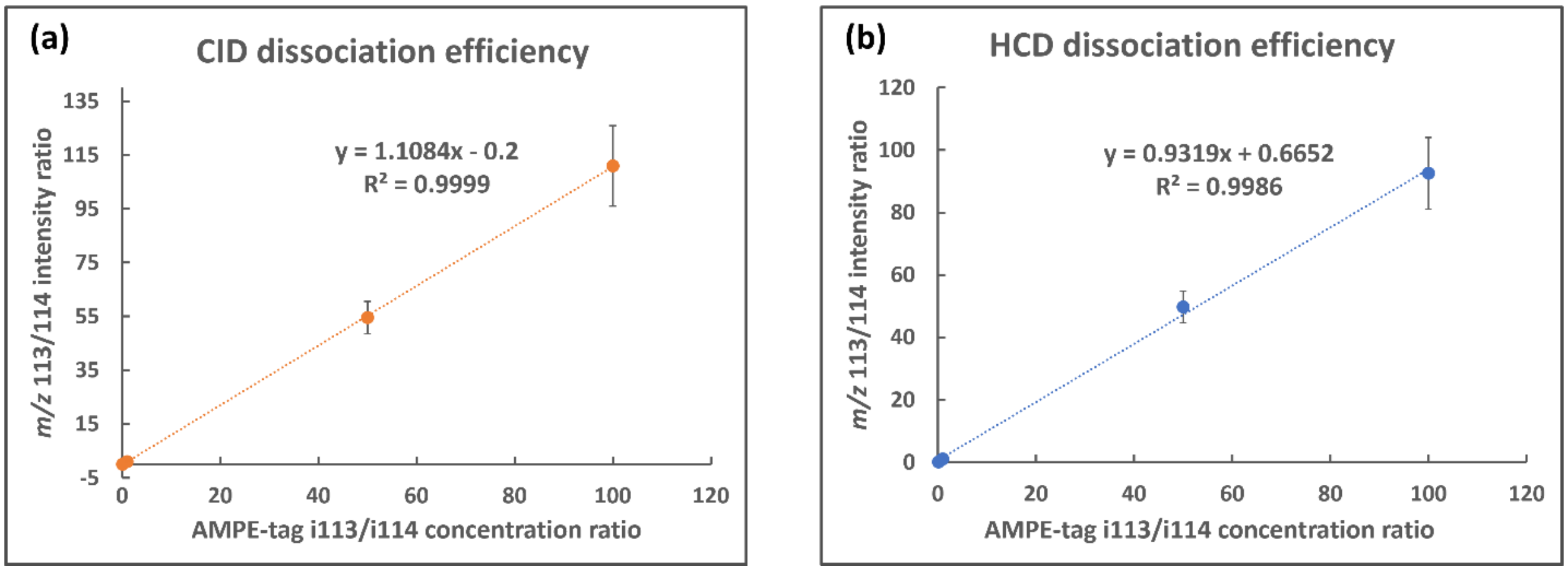

Figure S8. Dissociation efficiency with concentration for AMPE-tag i113 and i114 upon (a) CID and (b) HCD. In both dissociation methods, the duplex tags displayed identical dissociation efficiency with changed cocentrations.

S4. Optimization of PB reaction with PC 18:1(9)/18:1(9)

Optimization was done with non-isotope labeled AMPE-tag and PC 18:1(9)/18:1(9), as both reactant and product could be observed in positive ion mode. PC conversion rate was calculated for PB reaction yield, with the formula PC conversion rate = $\mathbf{I}([PC+Tag_{PB}+H]^+)/(\mathbf{I}([PC+H]^+)+\mathbf{I}([PC+Na]^+)+\mathbf{I}([PC+Tag_{PB}+H]^+))$, and here **I** represents intensity. Nitrogen purging time, lipid-tag ratio, solvent, and reaction time were investigated, with a triplicated experiment.

S4.1 Nitrogen purging investigation

Nitrogen purging is recommended to remove trace O_2 contained in solutions, as it could lead to oxidation and degradation of lipids, which decreases the yield of the PB reaction.[13] Nitrogen purging was conducted by sealing the microcentrifuge tube with two layers of parafilm (Parafilm "M"; Fisher Scientific, Pittsburgh, PA) and a 22*g* stainless steel needle (Air-Tite, Virginia Beach, VA) was inserted to pump N_2 into the solutions. Purging time varied from 0 to 15 min to examine the effect of purging time on PB reaction yield. The result shows that N_2 purging can help improve PB reaction yield, and a short time of 1 min has already made a difference (PC conversion rate 56% without N_2 purging and 78% with N_2 purging for 1 min). Purging for a longer time doesn't give a higher yield.

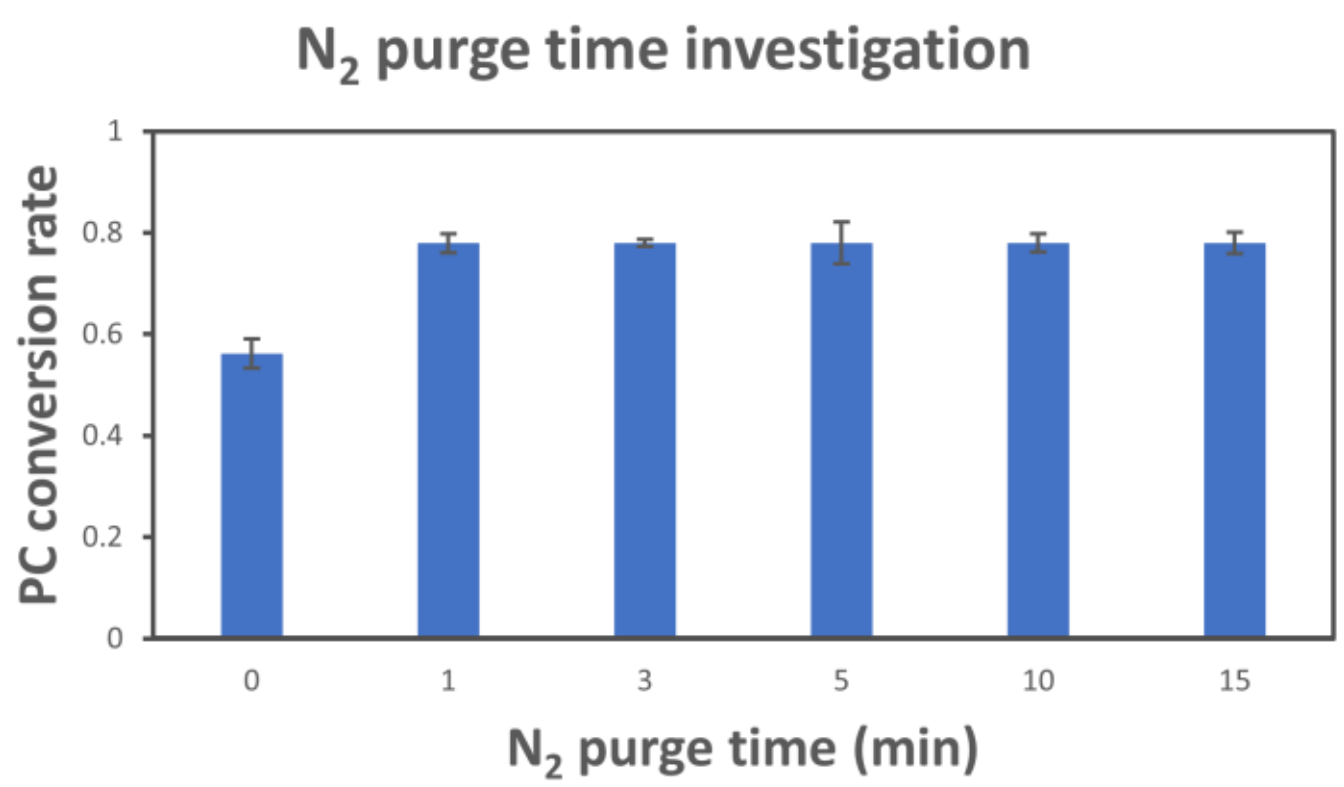

Figure S9. Investigation of N_2 purging time on PB reaction yield. N_2 purging improves reaction yield by removing trace O_2 in solutions and alleviating lipid radical-induced oxidation and degradation. A short time of 1 min purging is enough for the improvement. Reaction condition: PC 18:1(9)/18:1(9) 100 µM, AMPE-tag 100 µM, reactor tube 880 mm, flow rate 0.2 mL/min, reaction time 2 min, purging time 0-15 min, in ACN.

S4.2 Lipid-tag ratio investigation

PC 18:1(9)/18:1(9) 1 µM, 10 µM and 100 µM were reacted with AMPE-tag with ratios from 1:1 to 1:500, respectively. The reaction yield was related to tag concentration, as well as lipid concentration. When the lipid was at low concentration (1 µM in this case), much excessive tag was required for the reaction to happen. Only with a lipid-tag ratio higher than 1:100 (lipid 1 µM, tag 100 µM), the PB product was observed. And with the lipid-tag ratio reaching 1:500, the reaction yield reached 70% to 80%. With higher PC concentration (PC 10 µM), lipid-tag ratio only required 1:50 to get to 70%-80% yield. When PC concentration came to 100 µM, even 100 µM tag (lipid-tag ratio 1:1) could give that yield, and an increase in tag didn't affect the yield much. Therefore, excessive tag was recommended to give a consistent yield for lipids with a concentration in a wide range (1 to 100 µM).

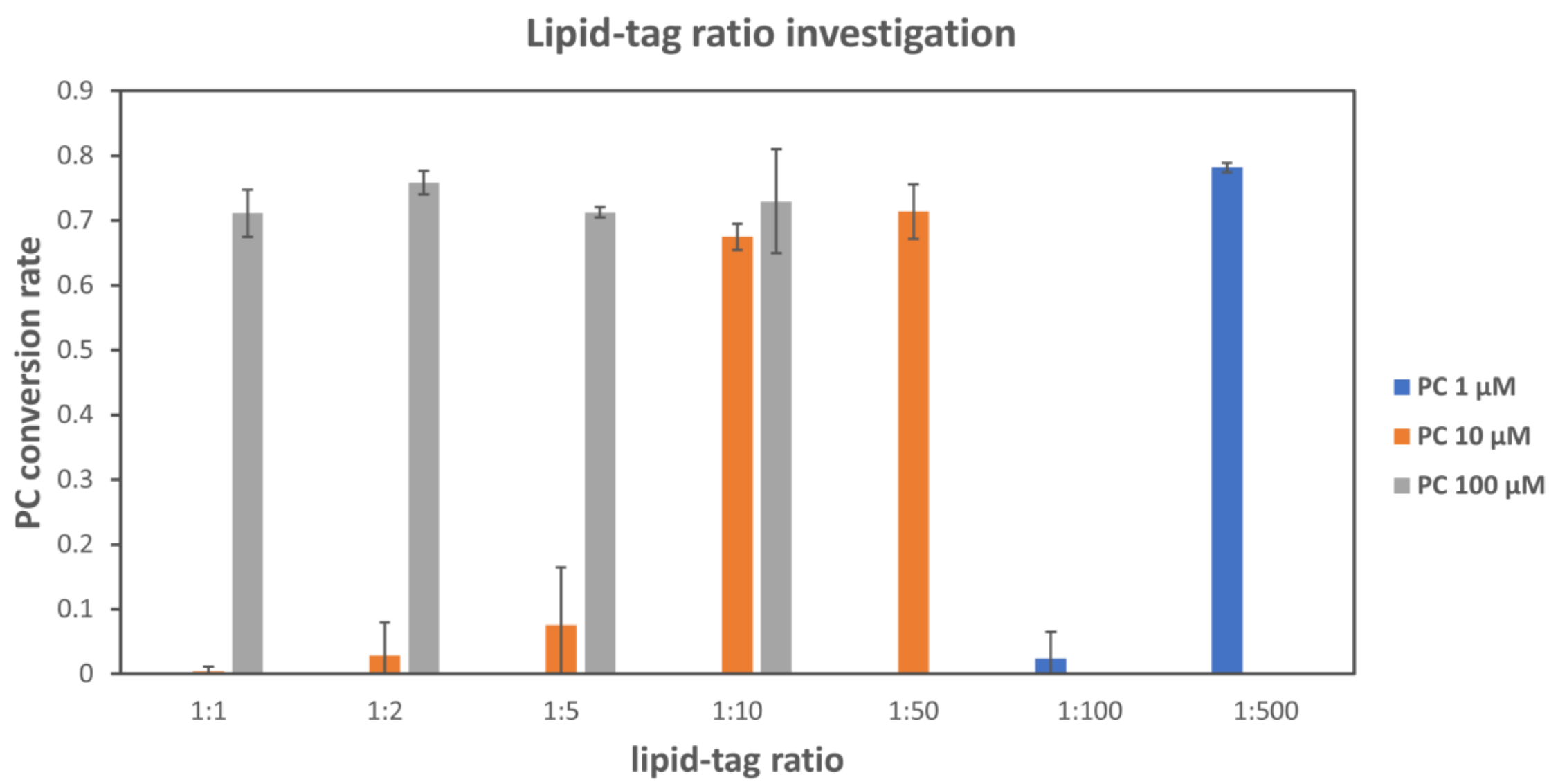

Figure S10. Investigation of lipid-tag ratio on PB reaction yield, with lipid concentration at 1, 10 and 100 µM. An excessive tag was required to give consistent and best yield for lipids at different concentrations. Reaction condition: PC 18:1(9)/18:1(9) 1 to 100 µM, AMPE-tag 1 to 1000 µM, reactor tube 880 mm, flow rate 0.2 mL/min, reaction time 2 min, purging time 10 min, in ACN.

S4.3 Solvent investigation

Different solvents were also tested for optimized reaction yield. Pure ACN gave the best yield, while mixing with various protonic solvents led to a decrease in yield. Chloroform was deleterious for this PB reaction, with 5% (V/V) addition lowering the reaction yield to less than 20%, which may be due to the active reactivity of chloroform to light. The addition of DCM was also harmful to this reaction, but 10% addition could still give a yield of 40%. Though pure ACN is the best for PB reaction of AMPE-tag, mixing with other solvents such as DCM is inevitable, as some lipids, especially at high concentrations, can't dissolve completely in ACN.

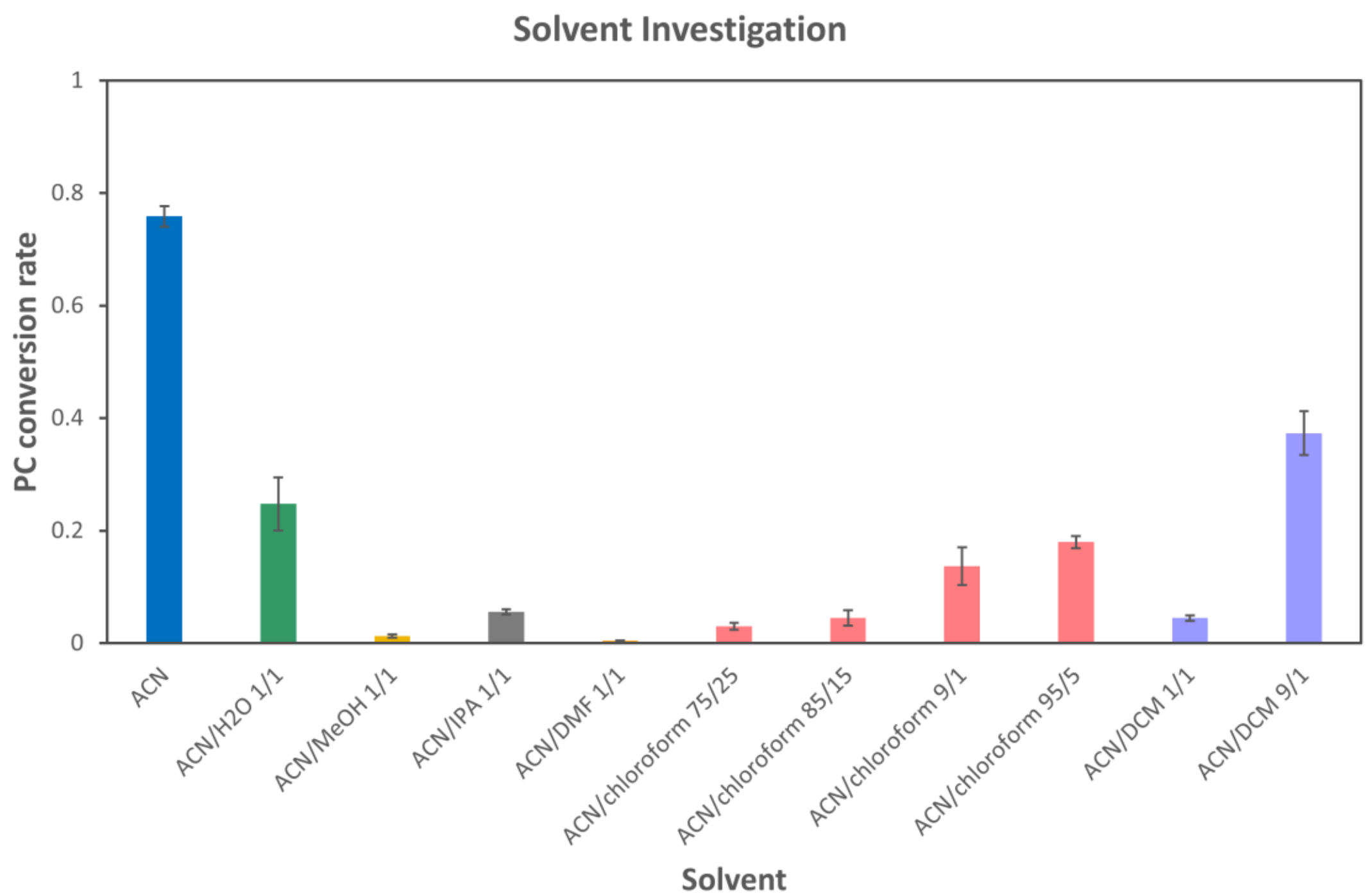

Figure S11. Investigation of the solvent on the PB reaction yield. Reaction showed the best yield in pure ACN, and mixing with 10% DCM could still give a yield of 40%, while mixing with other solvents interfered with the reaction greatly. Reaction condition: PC 18:1(9)/18:1(9) 100 µM, AMPE-tag 200 µM, reactor tube 880 mm, flow rate 0.2 mL/min, reaction time 2 min, purging time 10 min, in various solvent systems.

S4.4 Reaction time investigation

Reaction time is susceptible to two factors: the length of the FEP tubing for photoirradiation, and the flow rate of the solution through the FEP tubing. Hence, these two factors were both investigated. FEP tubing length was tested from 110 mm to 880 mm, with a flow rate of 0.1 mL/min, which resulted in reaction time from 0.5 min to 4 min. The reaction yield showed little difference in this time range, with reaction time 1 to 1.5 min having a slightly higher yield than the others. The flow rate effect was checked by pumping the solution through the tubing of 440 mm at the rates of 0.05, 0.1, and 0.2 mL/min, respectively. Yield with reaction time 1-2 min (flow rate 0.2 mL/min and 0.1 mL/min) was slightly higher than that of 4 min (flow rate 0.05 mL/min). When reaction time remained the same at 1 min, with various tubing lengths and flow rates. There wasn't much distinction, and either changing the tubing length or flow rate could work for changing the reaction time.

In ACN, the reaction yield reached its best when the reaction time was less than 2 min, but the difference was not very obvious. However, when DCM was added into the solvent, shorter reaction was usually required. Examination of reaction time was done in ACN/DCM 9/1 with a flow rate of 0.2 mL/min to 0.8 mL/min (Figure S13). The reaction yield decreased with longer reaction time, and the trend was more obvious than in ACN. Therefore, when a reaction is taken in an ACN/DCM mixture, a shorter reaction time is recommended.

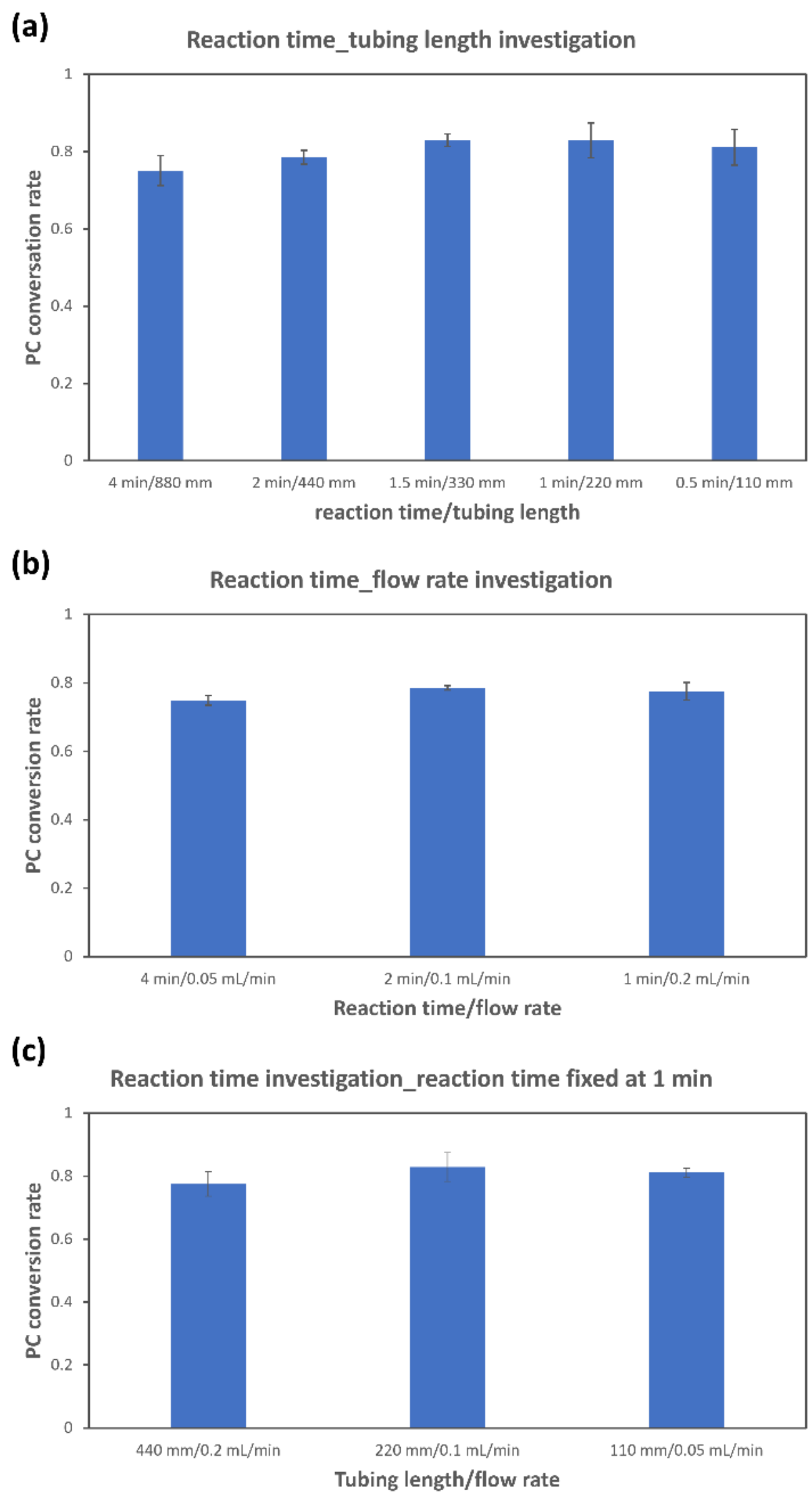

Figure S12. Investigation of the reaction time on the PB reaction yield. (a) FEP tubing length changed and resulted in a reaction time from 0.5 to 4 min; (b) flow rate changed and resulted in a reaction time from 1 to 4 min; (c) reaction time remained the same with various tubing lengths and flow rates. Reaction condition: PC 18:1(9)/18:1(9) 100 µM, AMPE-tag 200 µM, reactor tube 110 to 880 mm, flow rate 0.05 to 0.2 mL/min, reaction time 0.5 to 4 min, purging time 10 min, in ACN.

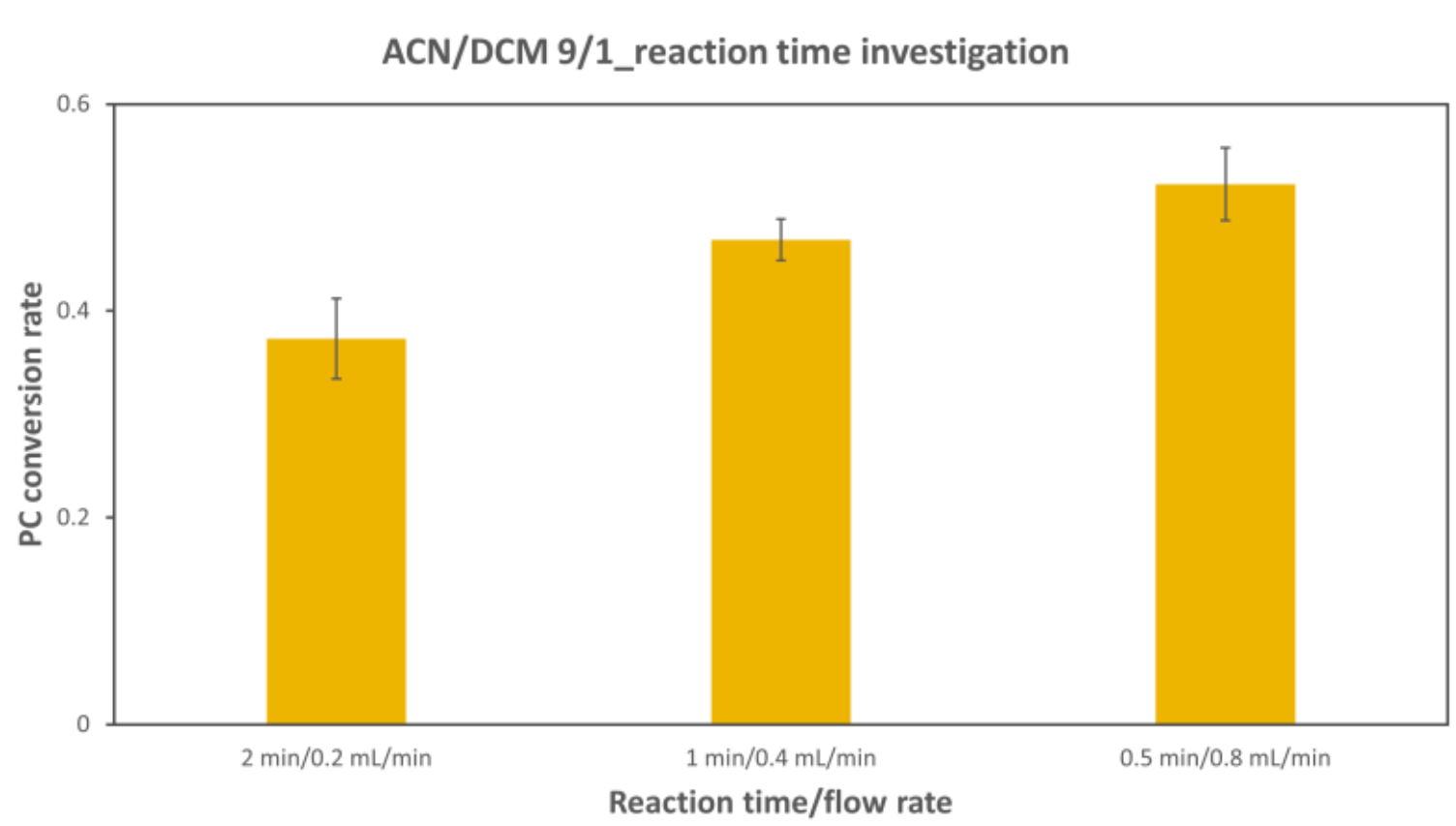

Figure S13. Investigation of reaction time on PB reaction yield in ACN/DCM 9/1. The reaction yield increased with shorter reaction time, and the trend was more obvious than in ACN. Reaction condition: PC 18:1(9)/18:1(9) 100 μM, AMPE-tag 200 μM, reactor tube 880 mm, flow rate 0.2 to 0.8 mL/min, reaction time 0.5 to 2 min, purging time 10 min, in ACN/DCM 9/1.

S5. PB reaction between AMPE-tag and lipids from different categories

After optimization, PB reaction via AMPE-tag was validated and investigated with PC 18:1(9)/18:1(9), and lipids from different categories were tested as well. All the lipids were kept at 100 µM, and reacted with non-isotopically labeled AMPE-tag at 200 µM. The reaction mixture was purged with N_2 for 10 min before UV irradiation. Reaction time was 2 min, with a flow rate of 0.2 mL/min and tubing length 880 mm. For PCs, FAs, and FAEEs, ACN was used as a solvent as they could be readily dissolved. For the other lipids, an ACN/H_2O solvent system was required for a clear reaction solution before irradiation.

S5.1 Investigation of PB reaction of AMPE-tag with PC 18:1(9)/18:1(9)

AMPE-tag reacted with PC 18:1(9)/18:1(9) under the irradiation of UV light of 254 nm. Mono-PB product (PC + tag) was the major product, since the di-PB product (PC + 2tags) was hardly observed: *m/z* 1365.96 ($[M+H]^+$), *m/z* 682.98 ($[M+2H]^{2+}$), and *m/z* 456.32 ($[M+3H]^{3+}$) were of very low intensity in the spectra after reaction (Figure S14). A structure yielded after one oxetane ring opening from the di-PB product could be found (*m/z* 965.63), which may imply that di-PB products are not stable. Therefore, the mono-PB product was mainly investigated in this study. For C=C position identification, CID was applied to cleave the PB product. The mono-PB product of PB 18:1(9)/18:1(9) had a singly-charged form at *m/z* 1075.78 and a doubly-charged form at *m/z* 538.39; however, dissociation upon CID of the doubly-charged mono-PB product gave very few diagnostic ions for C=C position identification (Figure S15). Three diagnostic ions were expected (structures shown in Figure S16), but one diagnostic ion (*m/z* 400.33) was missing, which may not be favored upon CID.

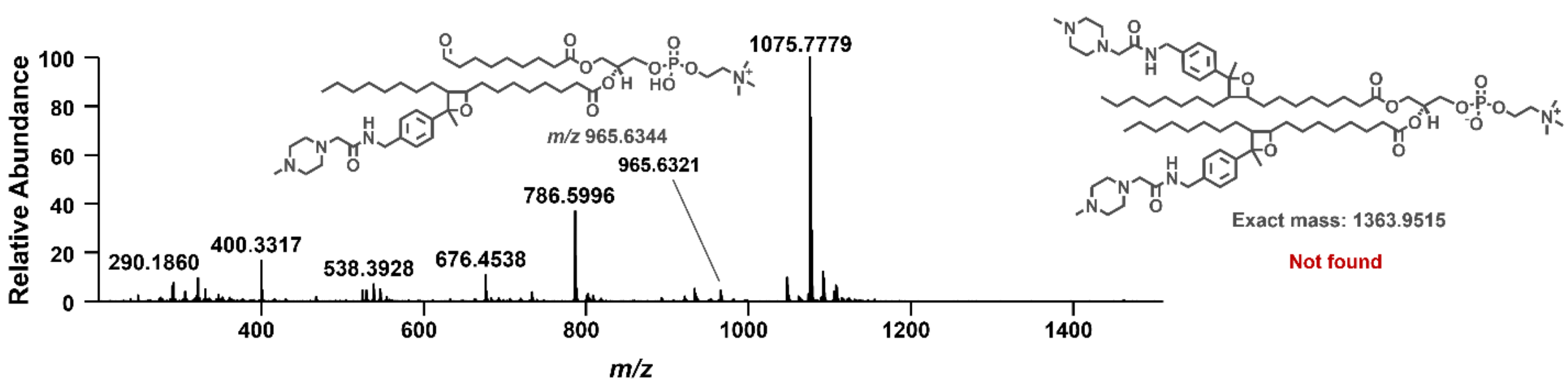

Figure S14. Full MS spectrum of PC 18:1(9)/18:1(9) 100 µM with AMPE-tag 200 µM in ACN after PB reaction, mass range 200-1500 in positive ion mode. Mono-PB product was the major PB product.

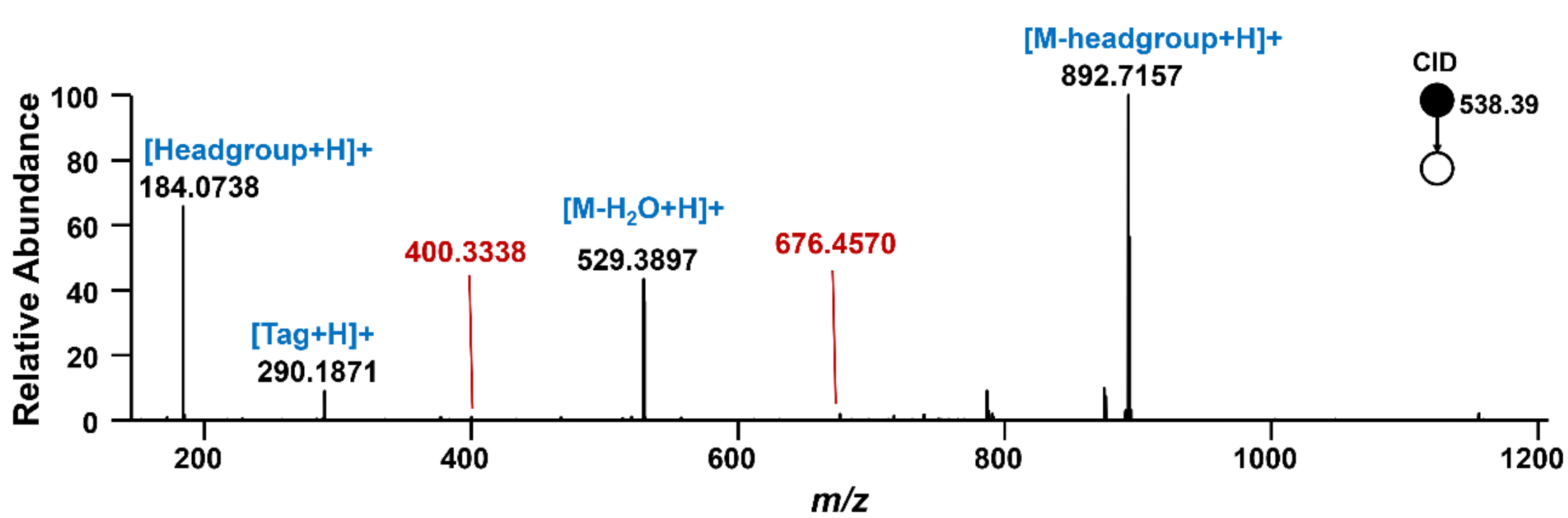

Figure S15. MS/MS spectrum of the doubly-charged product of PC 18:1(9)/18:1(9) and AMPE-tag. Diagnostic ions are labeled in red, but their intensities are very low.

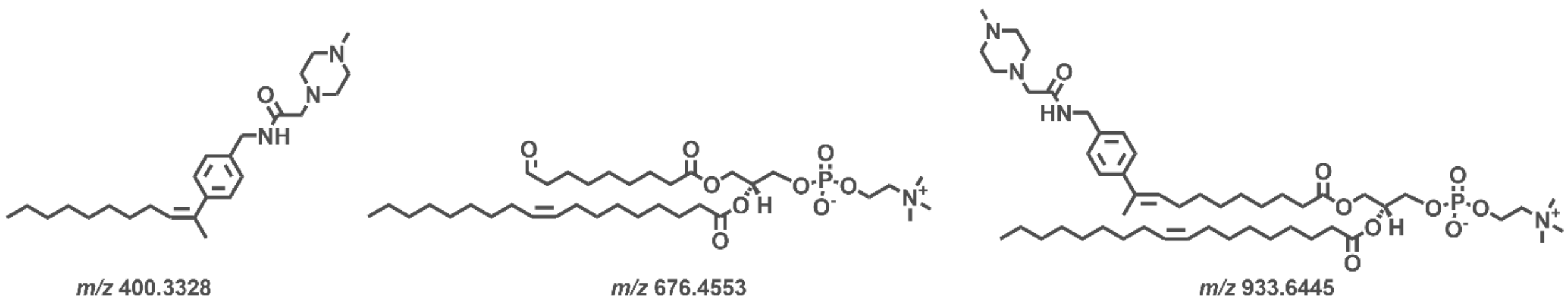

Figure S16. Structures and theoretical *m/z* of the three diagnostic ions of PC 18:1(9)/18:1(9) mono-PB product

Note: Preferential formation of mono-PB products

The predominance of mono-PB addition is likely a consequence of the short microflow irradiation conditions and the lower probability of a second PB reaction occurring on the already derivatized lipid. After the first PB addition, the lipid carries a bulky AMPE-derived oxetane structure, which can reduce the efficiency of further C=C derivatization. Limiting the irradiation time also suppresses over-reaction and photochemical side products. Similar preferential formation of mono-PB products has been observed in prior PB-based lipid-analysis studies (J. Zhao, X. Xie, Q. Lin, et al. Next-generation Paternò–Büchi reagents for lipid analysis by mass spectrometry. *Anal Chem*. **2020**, *92*, 13470-13477), supporting mono-addition as a common outcome under controlled photochemical derivatization conditions.

S5.2 Lipids from various categories after PB reaction with AMPE-tag

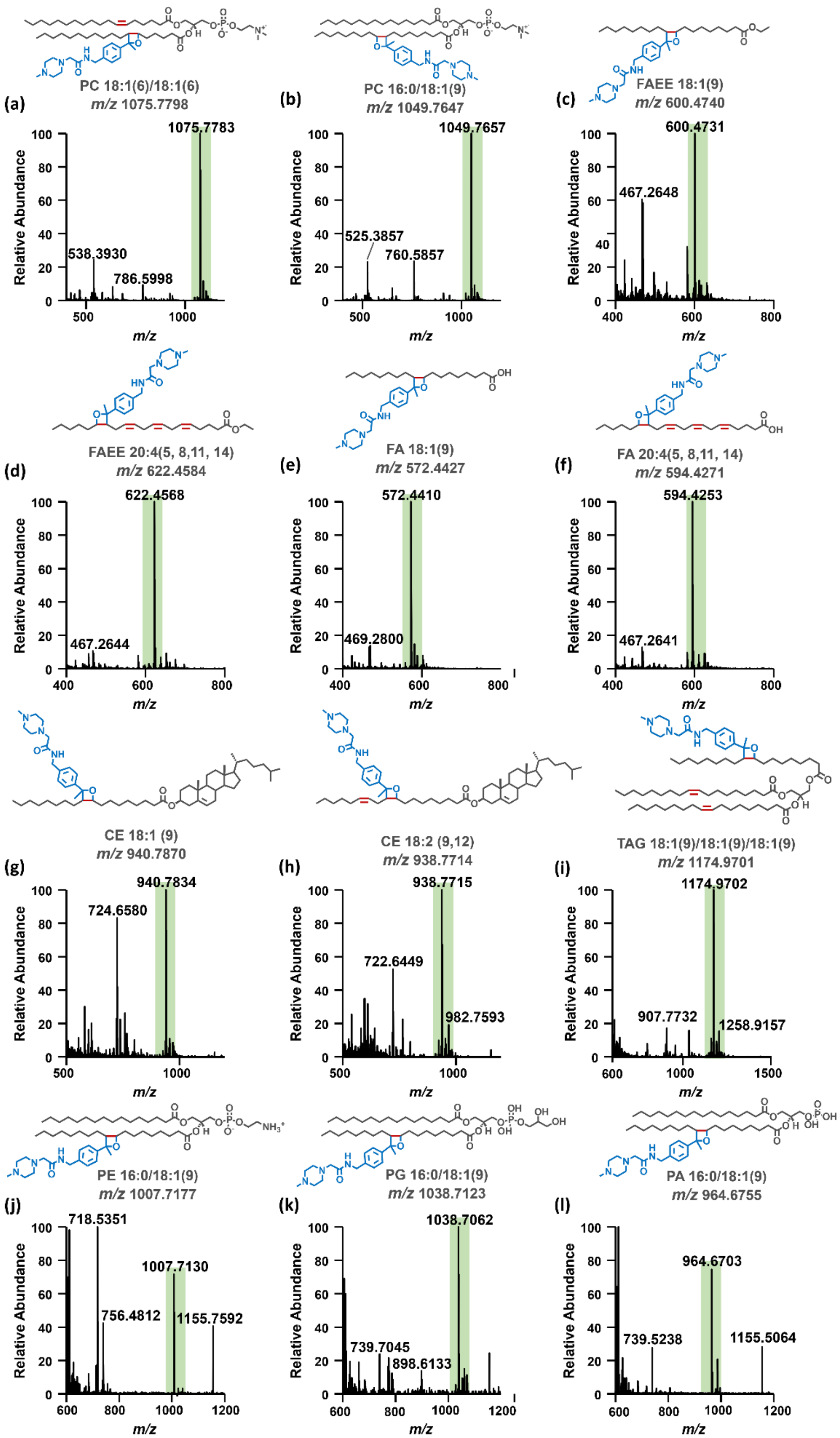

Figure S17. Full mass spectra of various lipids after PB reaction with non-isotope-labeled AMPE-tag. (a) PC 18:1(6)/18:1(6); (b) PC 16:0/18:1(9); (c) FAEE 18:1(9); (d) FAEE 20:4(5,8,11,14); (e) FA18:1(9); (f) FA 20:4(5,8,11,14); (g) CE 18:1 (9); (h) CE 18:2(9,12); (i) TAG 18:1(9)/18:1(9)/18:1(9); (j) PE 16:0/18:1(9); (k) PG 16:0/18:1(9); (l) PA 16:0/18:1(9). Product structures and theoretical *m/z* are provided above the spectra, and the product peaks are labeled in green.

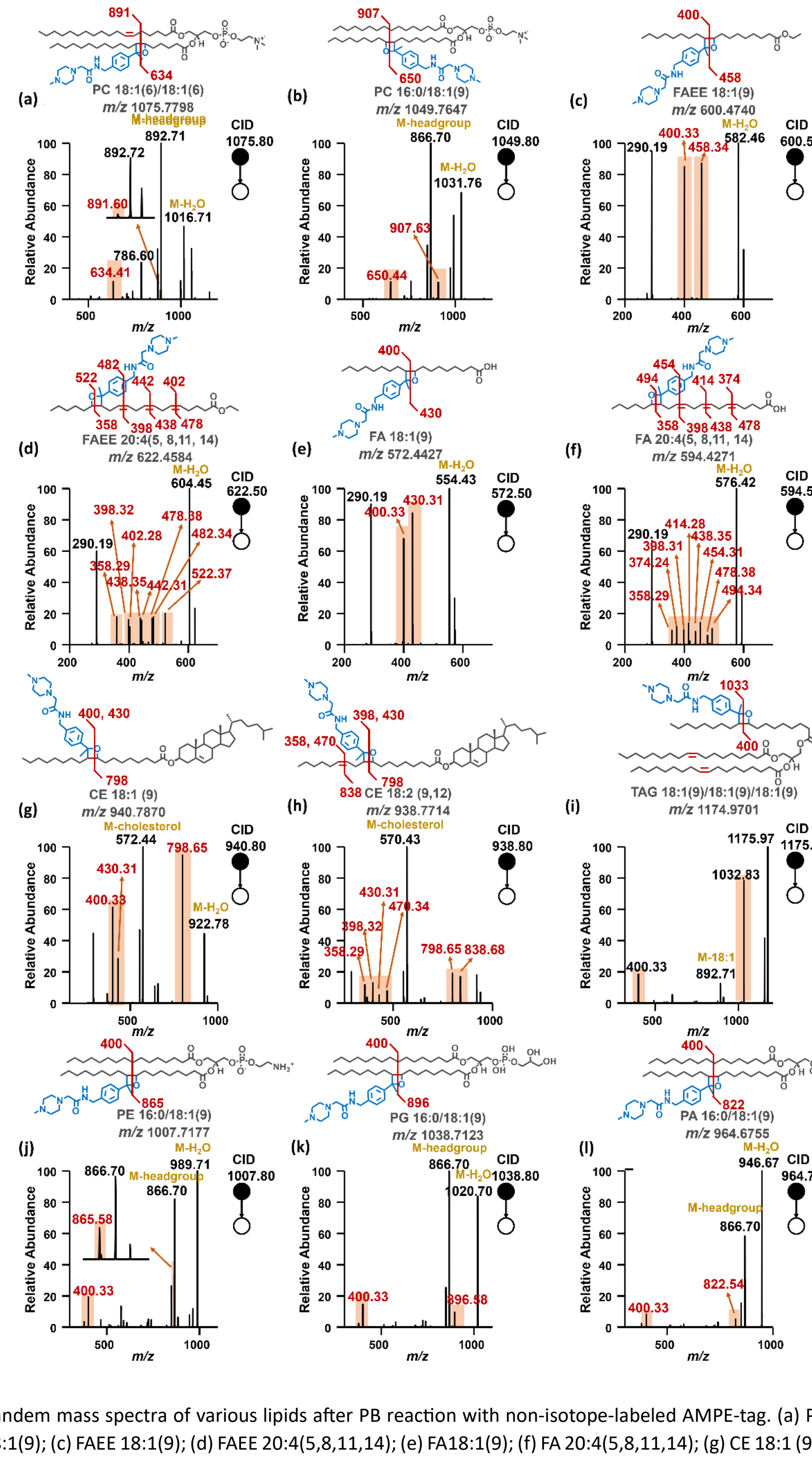

Figure S18. Tandem mass spectra of various lipids after PB reaction with non-isotope-labeled AMPE-tag. (a) PC 18:1(6)/18:1(6); (b) PC 16:0/18:1(9); (c) FAEE 18:1(9); (d) FAEE 20:4(5,8,11,14); (e) FA18:1(9); (f) FA 20:4(5,8,11,14); (g) CE 18:1 (9); (h) CE 18:2(9,12);

(i) TAG 18:1(9)/18:1(9)/18:1(9); (j) PE 16:0/18:1(9); (k) PG 16:0/18:1(9); (l) PA 16:0/18:1(9). Cleavage is shown for diagnostic ions generation, and diagnostic ions are labeled in orange in the spectra.

S6. Condition investigation for tagging FAs with the duplex AMPE-tags

The tagging of AMPE-tag to FAs was conducted in ACN/DCM 9/1 solvent system to ensure complete dissolution of lipids. The flow rate needs to be faster for better reaction yield (Fig. S13), and therefore, the flow rate was set as 0.4 mL/min for a total reaction time of 1 min, with tubing length of 880 mm. Excessive AMPE-tags of 2.5 mM were added to FA solutions, 50 eq. to 50 µM (the reference conc.), to ensure maximum conversion of tagged FAs. N_2 purging time was shortened to 2 min to decrease uncertainty within the process (e.g., evaporation).

CE of HCD and CID and isolation window width (IWW) were investigated for 50 µM FA 18:1(9) after reaction with a mixture of 2.5 mM AMPE-tag i113/i114 1:1 with conditions above. It was observed that the tagged FAs with AMPE-tag i113 or i114 were similar to each other, and the pattern of mass reporter ions generation was also similar to the tag itself, with mass reporter ions dominating the tandem MS spectra and little difference in CE required for HCD. For mass reporter ions indicating total FA 18:1(9) relative abundance, the CE and IWW of HCD for MS^2 were changed from 30 to 55 at a step size of 5, and 0.2 to 3, respectively, and 113/114 intensities were normalized by TIC to compare which conditions gave the best dissociation efficiency of mass reporter ions. For mass reporter ion indicating isomer abundance, they are released at MS^3 and can be affected by CID for MS^3 and then HCD for MS^3. Thus, CE and IWW for both CID and HCD were interrogated on the generation of diagnostic ions and subsequent mass reporter ions. The results showed that mass reporter ions at MS^2 reached the optimized dissociation efficiency at CE 45 and IWW 1.0; while mass reporter ions at MS^3 reached the maximum at CID CE 40, IWW 2.0 at MS^2 and HCD CE 40, IWW 2.0 at MS^3.

S6.1 CE investigation of tagged FAs dissociation upon HCD and CID

Generation of mass reporter ions for quantitation of the FAs at the total level was related to CE of HCD for MS^2. When CE was in the range of 45 and 55, the dissociation efficiency didn't increase anymore and remained the most with a normalized intensity of 113/114 at about 0.23 (Figure S19a).

CE of CID for MS^2 rarely made a difference in the generation of diagnostic ions or mass reporter ions at the isomeric level. From CE 30 to 55, the results were consistent (Figure S19b-d).

However, HCD for MS^3 showed the best cleavage for mass reporter ions at CE 40, for both diagnostic ions at *m/z* 401.3 and *m/z* 431.3 (Figure S19e-f).

In conclusion, CE of HCD was selected at 45 for MS^2 and 40 for MS^3; with CE of CID at 40 for MS^2 in further experiments.

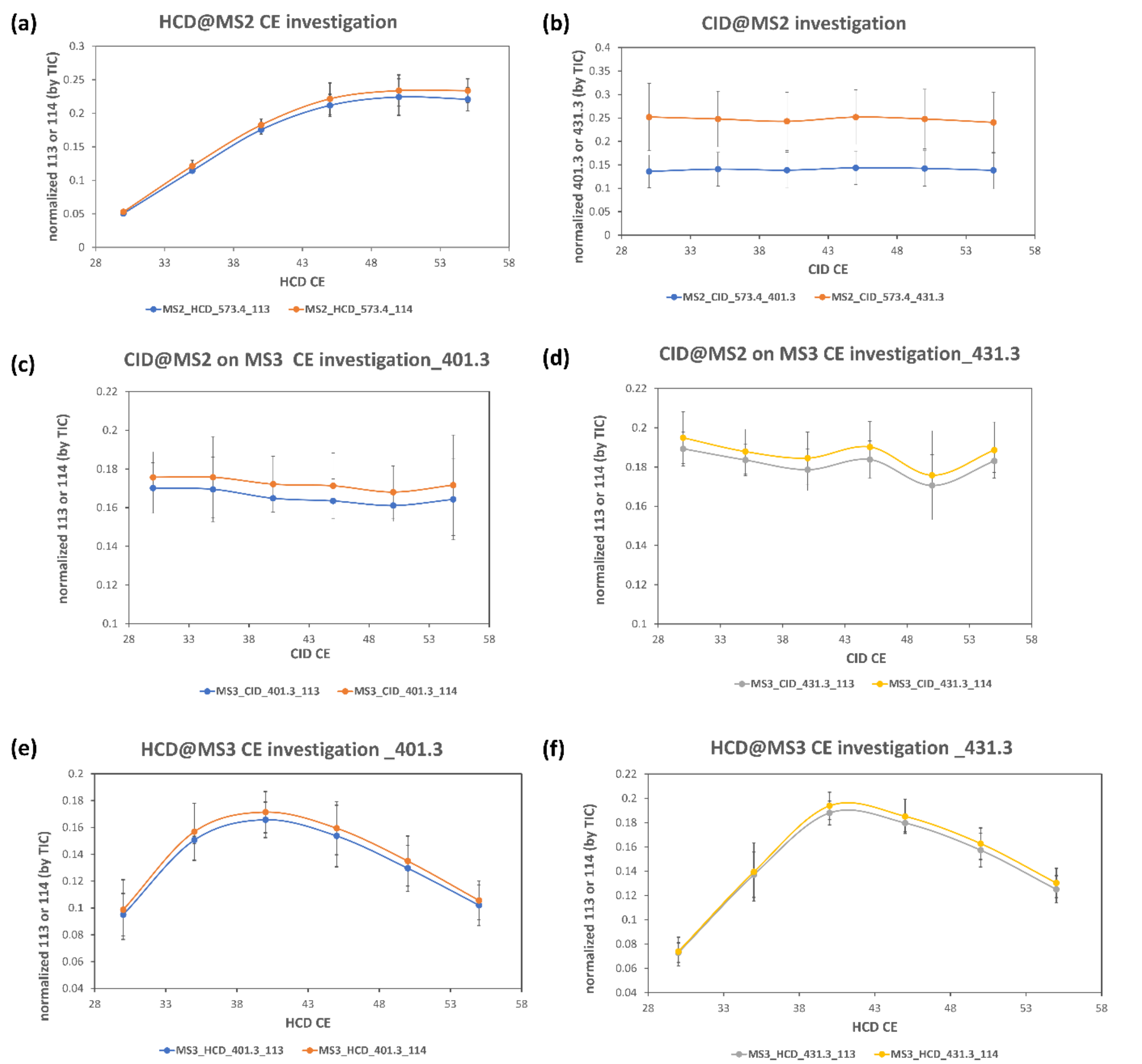

Figure S19. CE investigation with FA 18:1(9) and AMPE-tag i113/114. (a) HCD for MS^2 to release mass reporter ions for FA quantification at the total level. (b) CID for MS^2 to release diagnostic ions for FA C=C bond positional isomer identification. CID for MS^2 may also affect releasing mass reporter ions from diagnostic ions at (c) *m/z* 401.3 and (d) *m/z* 431.3. HCD for MS^3 was also checked from diagnostic ion (e) *m/z* 401.3 and (f) *m/z* 431.3.

S6.2 IWW investigation of tagged FAs dissociation upon HCD and CID

Isolation window width can play an important role as well. Too narrow an isolation window may not trap the ions completely, while too wide an isolation window can include irrelevant ions and cause distortion of quantification.[14] Therefore, IWW was investigated for both HCD and CID for MS^2 and MS^3. For HCD for MS^2 and MS^3, it showed sufficient trapping of ions when larger than 0.5 (Figure S20a, e, f). While for CID for MS^2, a larger IWW at 1 to 2 was required (Figure S20b-d). For mass reporter ions at MS^2, a smaller IWW was preferred to exclude contamination peaks; while for mass reporter ions at MS^3, IWW can be larger as the interference effect was partly resolved by additional isolation, and the ion intensities should be increased for better signals. Thus, IWW for HCD for MS^3 was selected at 1.0 to generate mass reporter ions at MS^2; and IWW were selected at 2.0 for CID for MS^2 and HCD for MS3 to generate mass reporter ions at MS^3.

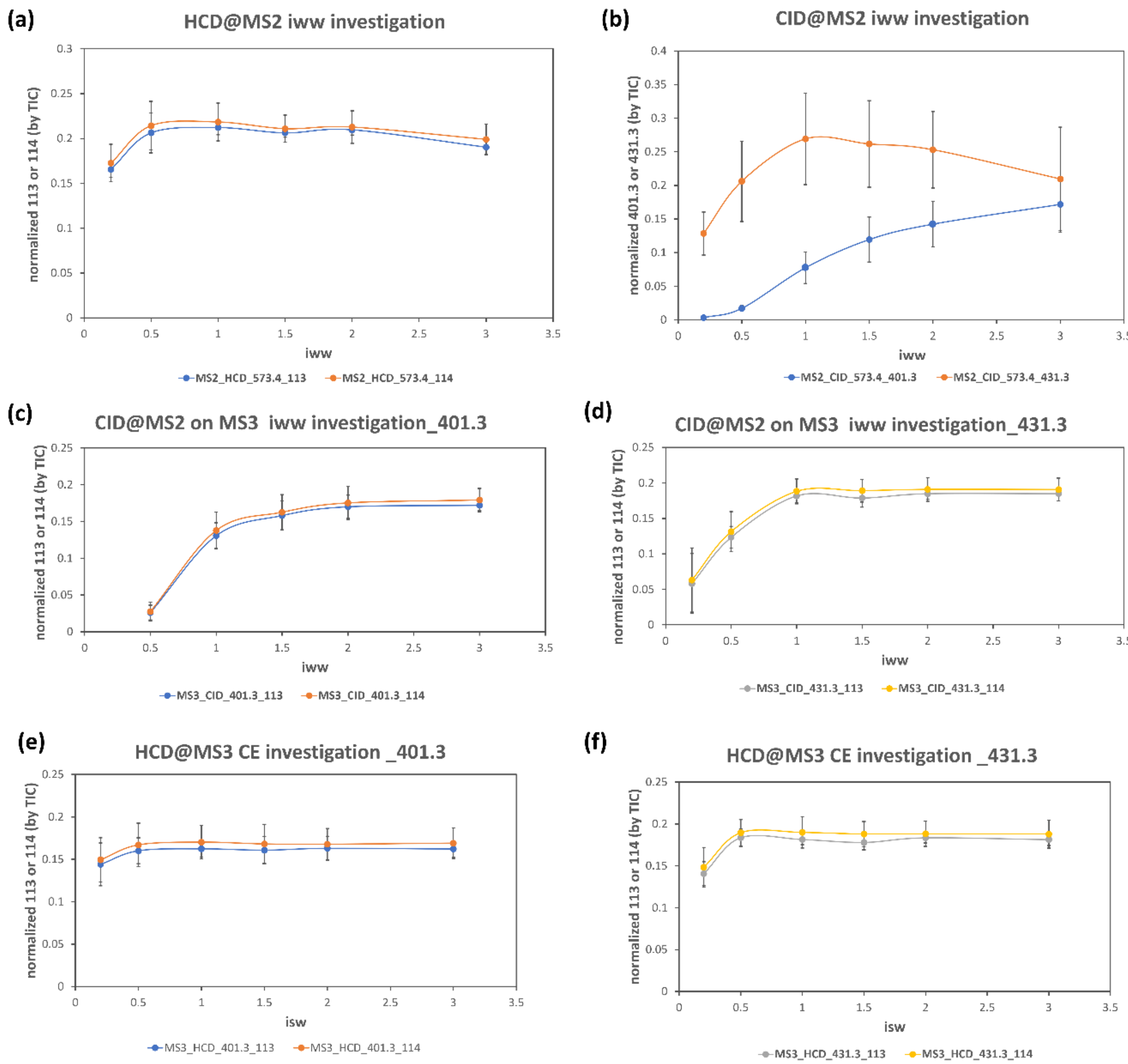

Figure S20. IWW investigation with FA 18:1(9) and AMPE-tag i113/114. (a) HCD for MS^2 to release mass reporter ions for FA quantification at the total level. (b) CID for MS^2 to release diagnostic ions for FA C=C bond positional isomer identification. CID for MS^2 may also affect releasing mass reporter ions from diagnostic ions at (c) *m/z* 401.3 and (d) *m/z* 431.3. HCD for MS^3 was also checked from diagnostic ions (e) *m/z* 401.3 and (f) *m/z* 431.3.

S7. Tag reactivity investigation for accurate quantification

S7.1 Unequivalent tag reactivity is the main factor of distorted quantification results

Equivalent tag reactivity is important for quantification. When a difference in reactivity exists, the concentration of lipids in different samples cannot be read directly from the intensity ratio of mass reporter ions. In Figure S21, the reactivity of AMPE-tag i113 was lower than that of AMPE-tag i114, and therefore, the 113/114 ratio was not 1, even though FA 18:1(9) was kept at 50 µM in two samples. Increasing the tag-to-lipid ratio could partly resolve the issue, but distortion in the quantification result was still observed. When building calibrations with isobaric tags with unequal reactivity, the slope of calibration curves was the same as that of unequal tag reactivity: when the reactivity of tag i113 was only 80% of that of i114, the slope was reduced to 0.8 as well (Figure S22). Therefore, maintaining the same reactivity for the two isobaric tags was the key to accurate quantification.

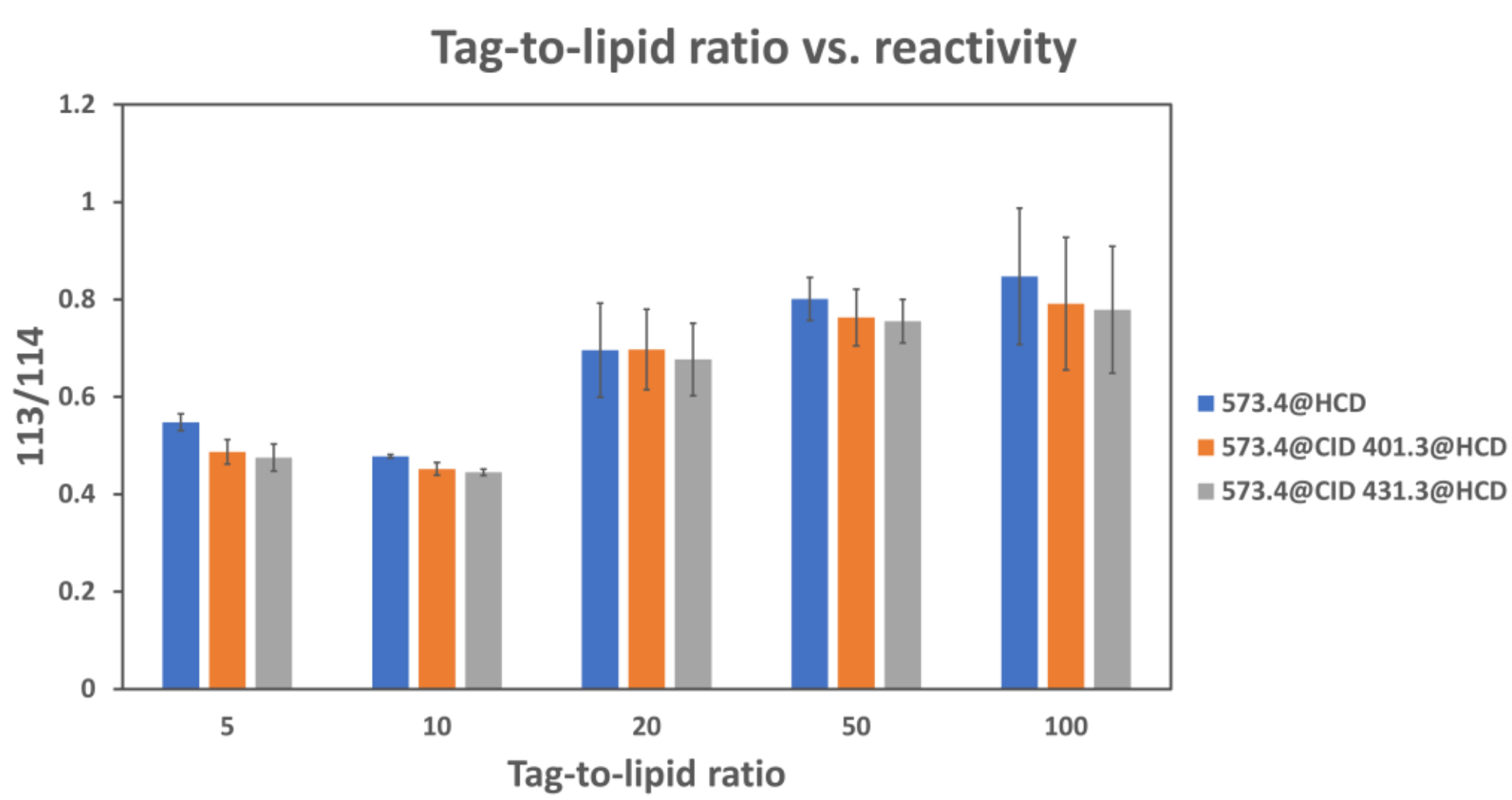

Figure S21. Unequivalent reactivity was found with tag i113 and tag i114. Tag i113 showed less reactivity than tag i114. A higher tag-to-lipid ratio reduced the difference, but not completely solved the issue. Here, the tag-to-lipid ratio was determined by the concentration of tag added and the concentration of lipids at 50 µM.

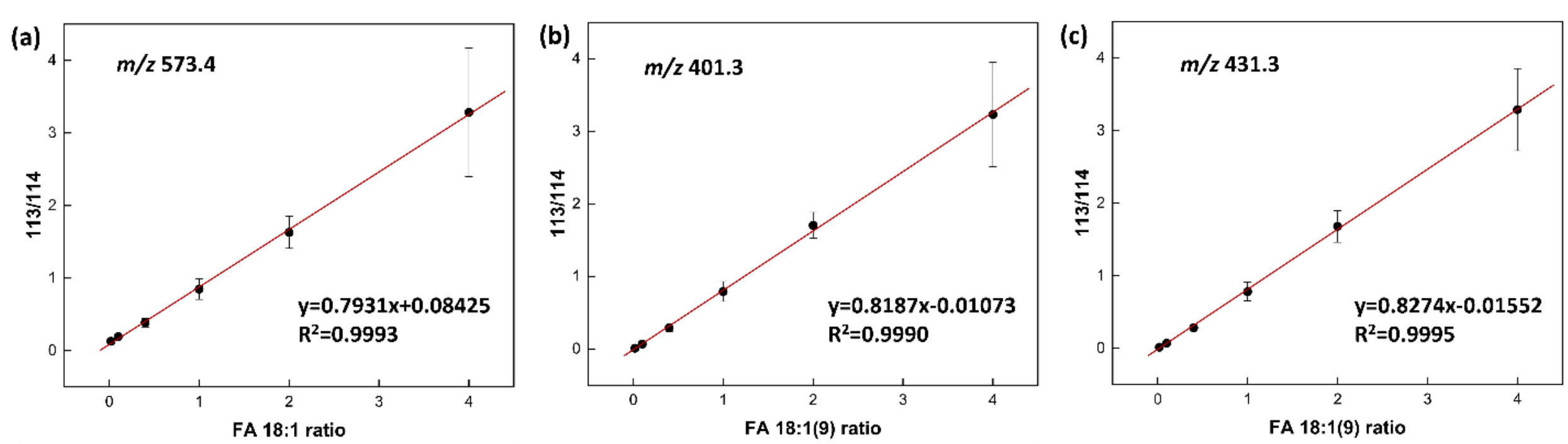

Figure S22. Quantification results of FA 18:1(9) and AMPE-tag with distinct reactivity. Tags at 100 eq. to FAs (reference at 50 µM) were added, under which condition, tag i113 only showed about 80% reactivity of tag i114. The reactivity was determined by reacting with FAs with the same concentration and then checking the intensities of mass reporter ions. With this method, FA 18:1(9) was tested, and the results were as follows: 113/114 was 0.84 at MS^2 (*m/z* 573.4), 0.79 at MS^3 (*m/z* 401.3), and 0.78 at MS^3 (*m/z* 431.3), respectively. The calibration curves built showed slopes of 0.8, showing deviation from 1, which may have resulted from the difference in tag reactivity.

S7.2 Factors affecting tag reactivity

Experiments were done to figure out factors affecting tag reactivities. The tag i113/i114 concentration was checked to ensure tags were tested at the same concentration. Tags i113 and i114 were mixed with the same volume from the stock solution and dissociated to release mass reporter ions. The ratio of mass reporter ion intensities was determined, and a 1:1 ratio was found (113/114=0.975, sd=0.18 by triplicate experiment), showing the equal concentration of tags in the stock solution. Different tag i113/i114 ratios, addition of formic acid and triethylamine (TEA) were tested with FA 18:1(9) and distinctly reactive tags. The tag-to-lipid ratio was chosen at 50, and for 50 µM FAs, 2.5 mM tags were added.

As excessive tags were added to solutions for reaction, adding tag i113/i114 a little more or less didn't make the result different (Figure S23a). However, adding TEA caused the rapid decrease in tag reactivity (Figure S23b). When 1.3% of TEA (v/v) was added to a 500 mM tag i114 stock solution, its reactivity was reduced to the same level as tag i113; and with the addition of 2.5% of TEA, the reactivity of tag i114 was only half that of tag i113. Too much addition of TEA may also cause too many side products and matrix effect, which causes the signals to drop, and the intensity ratio of the mass reporter ion from MS^2 and MS^3 is not equivalent. By contrast, adding formic acid didn't show much difference, with an increase in reactivity of i113 detected a little bit (Figure S23c). Therefore, one factor that may cause unequivalent tag reactivity is the TEA left in the tag, as some amount of TEA can result in decreased reactivity of tags. As TEA is used in the synthesis of AMPE-tag, purification is important. Tags with the same reactivity could be achieved with care in the synthesis, purification, and preparation of tag stock solutions.

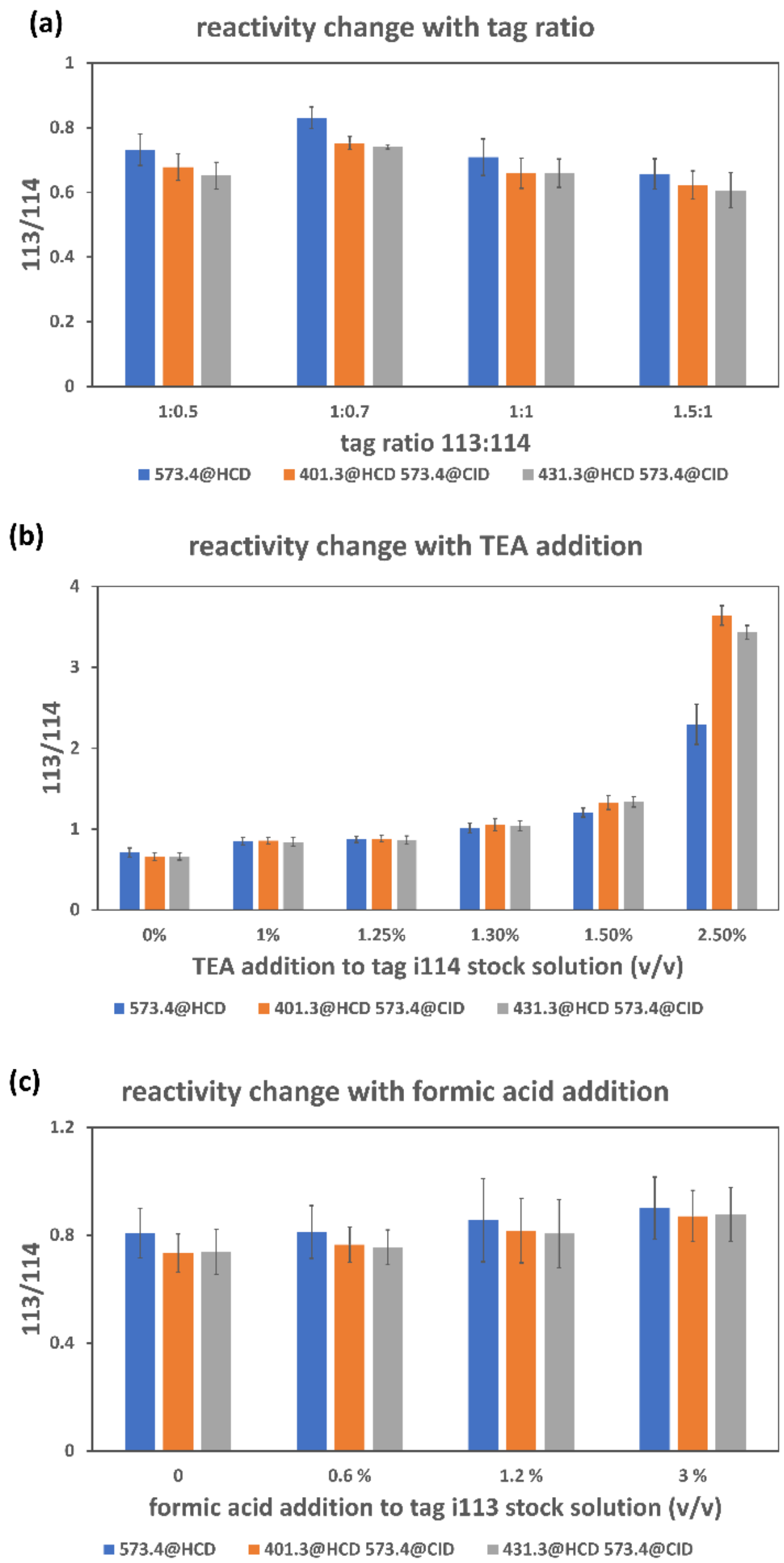

Figure S23. Investigation of factors possibly affecting tag reactivity: (a) tag i113/i114 ratio; (b) addition of TEA; and (c) addition of formic acid.

S7.3 Quantification results with tags adjusted by TEA

Although efforts were still put into obtaining tags with the same reactivity, here we also provided a method to do quantification with unequivalent reactivity after TEA adjustment. After adding 1.3% TEA (v/v) to the tag i114 500 mM stock solution, the reactivity between tag i113 and i114 was close to each other (Figure S24a). Calibration curves were accomplished with FA 18:1(9)/FA 18:1(6) 1:1 mixture from 1-200 µM as described in the main text. All the samples were tested with nanoESI. The slope of calibration curves was back to 1, indicating that the TEA-adjusted tag may also be feasible for quantification purposes.

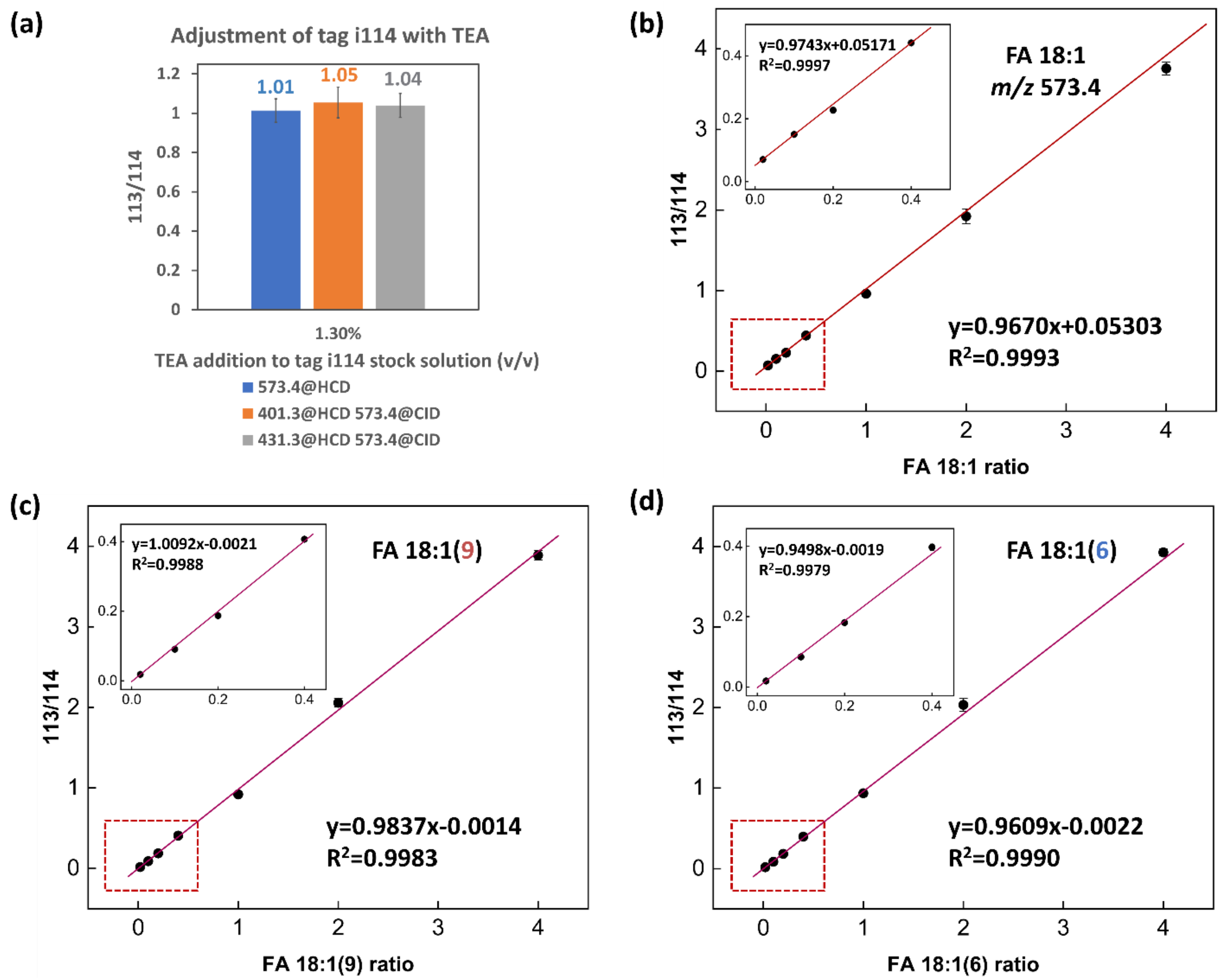

Figure S24. Quantification results of FA 18:1(9)/FA 18:1(6) 1:1 mixture and AMPE-tags, with tag i114 adjusted with the addition of 1.3% TEA. Calibration curves with good linearity and a slope close to 1 could still be achieved.

S8. Further validation of AMPE-tag quantification feasibility

S8.1 Quantification of FA 18:1(9)/FA 18:1(6) 1:1 mixture 1-200 µM vs50 µM

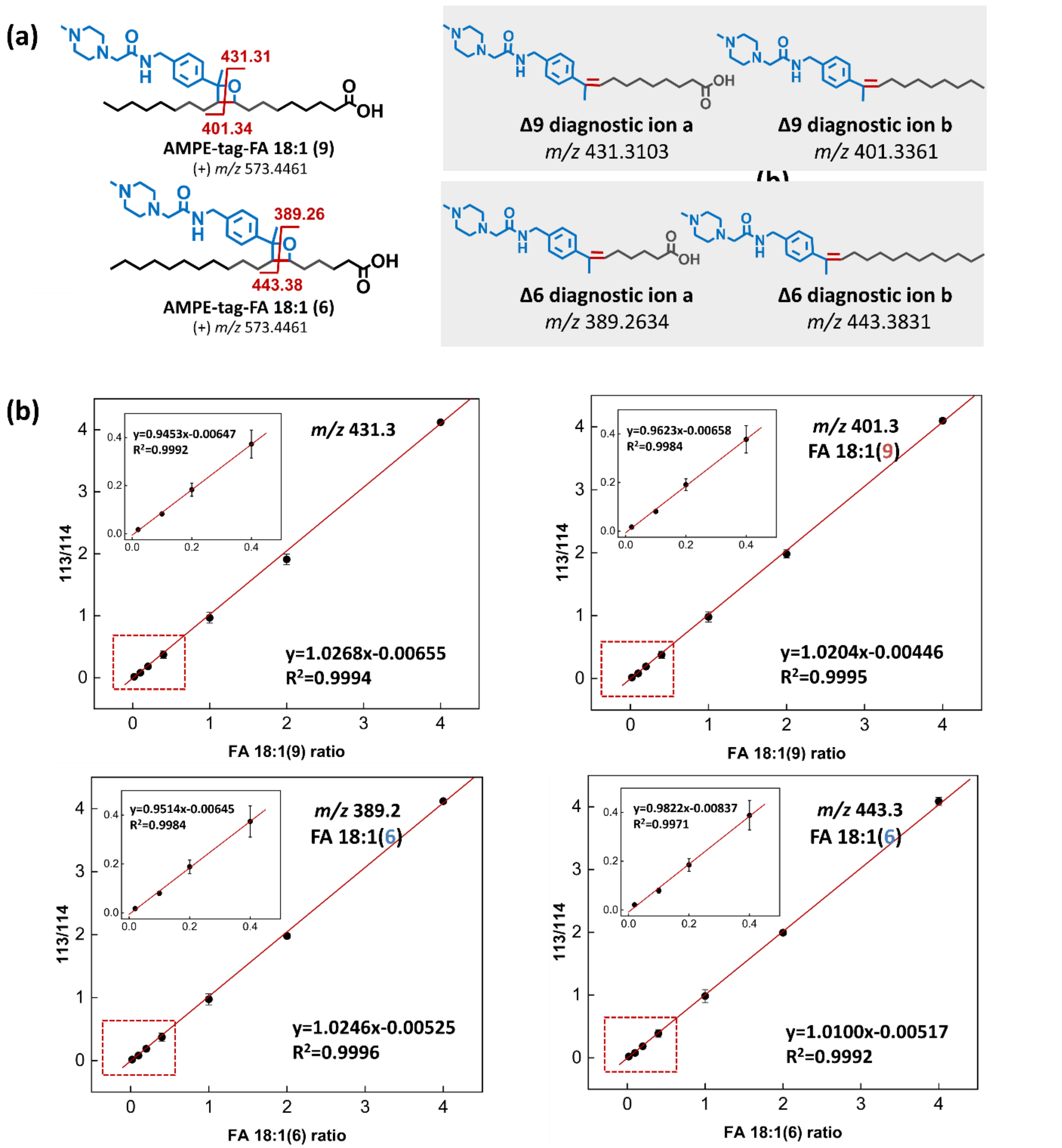

Figure S25. Quantification results of FA 18:1(9)/FA 18:1(6) 1:1 mixture at the isomer level using HPLC injection. The diagnostic ion structures and the calibration curves are shown in (a) and (b).

Table S1. Quantification results of FA 18:1(9)/FA 18:1(6) 1:1 mixture at the isomer level using nanoESI direct infusion. Accurate quantification could still be achieved even without the separation of the matrix.

Quantification Species	Diagnostic Ion	Calibration Curve	R^2
FA 18:1(9)	*m/z* 401.3	y=0.9602x-0.0010	0.9988

	m/z 431.3	y=0.9374x-0.0126	0.9996
FA 18:1(6)	*m/z* 389.3	y=0.9479x-0.0013	0.9986
	m/z 443.3	y=0.9694x-0.0019	0.9993

S8.2 Quantification of FA 18:1(9)/FA 18:1(6) 3:1 mixture vs. FA 18:1(9)/FA 18:1(6) 1:1 mixture

Quantification was also conducted with a FA 18:1(9)/FA 18:1(6) 3:1 mixture with concentrations ranging from 1-200 µM as samples and a FA 18:1(9)/FA 18:1(6) 1:1 mixture of 50 µM as reference using direct infusion. The ratios of FA 18:1 at the total level and at the isomeric level were different, with that of the total FA 18:1 varying between 0.02-4, FA 18:1(9) between 0.03-6, and FA 18:1(6) between 0.01-2 (Fig.S26a). Calibration curves were built, and different relative abundances of FA 18:1 at the total level and the isomeric level were successfully indicated.

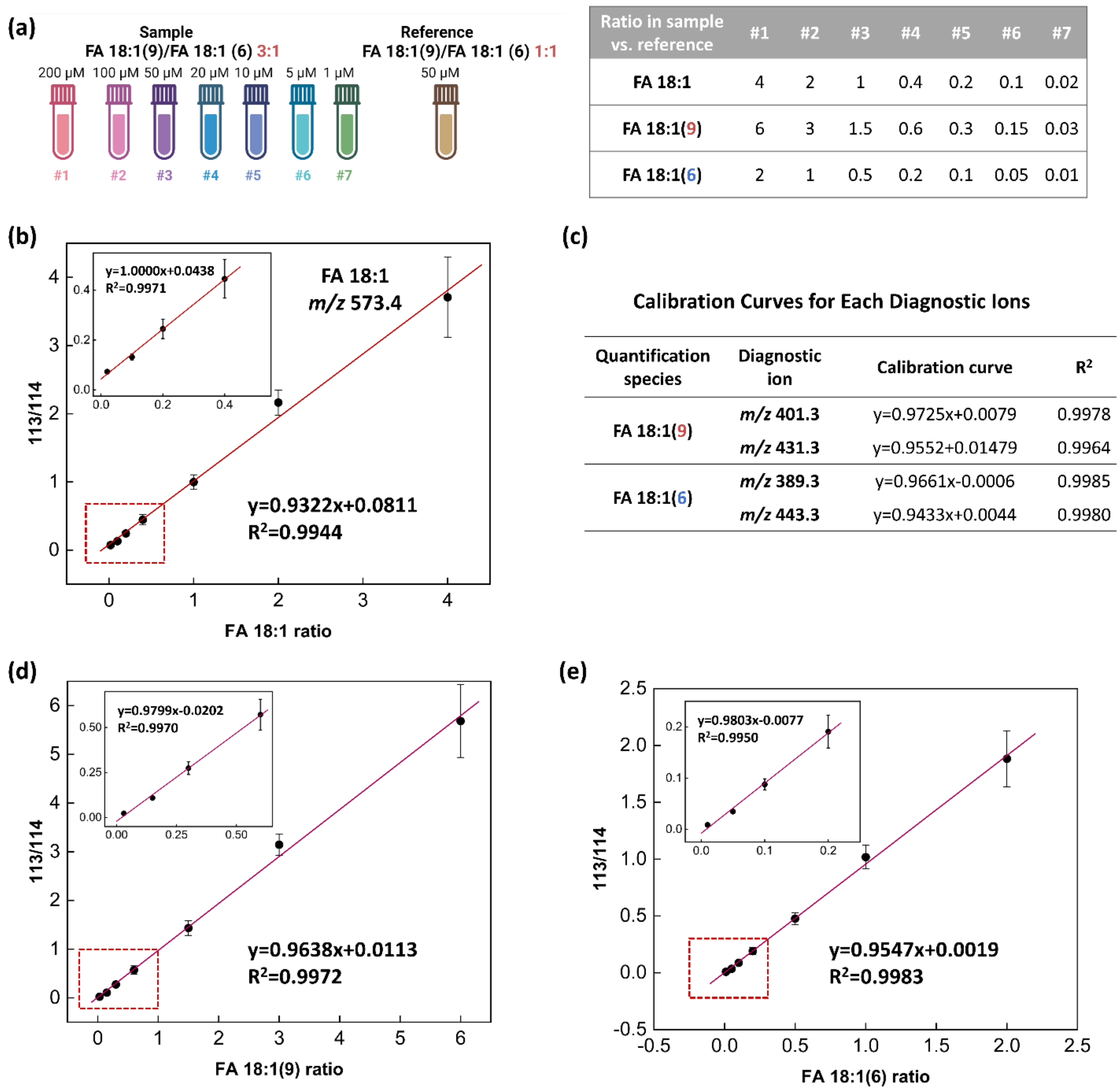

Ratio in sample vs. reference	#1	#2	#3	#4	#5	#6	#7
FA 18:1	4	2	1	0.4	0.2	0.1	0.02
FA 18:1(9)	6	3	1.5	0.6	0.3	0.15	0.03
FA 18:1(6)	2	1	0.5	0.2	0.1	0.05	0.01

Calibration Curves for Each Diagnostic Ions

Quantification species	Diagnostic ion	Calibration curve	R^2
FA 18:1(9)	*m/z* 401.3	y=0.9725x+0.0079	0.9978
	m/z 431.3	y=0.9552+0.01479	0.9964
FA 18:1(6)	*m/z* 389.3	y=0.9661x-0.0006	0.9985
	m/z 443.3	y=0.9433x+0.0044	0.9980

Figure S26. Quantification results of FA 18:1(9)/FA 18:1(6) 3:1 mixture as samples and 1:1 mixture as references. Different ratios of FA 18:1 at the total level and the isomeric level were indicated, and calibration curves were established.

S8.3 Quantification of FA 18:1(9)/FA 18:1(6) mixture with consistent concentration of FA 18:1(6)

Quantification was done with a FA 18:1(9)/FA 18:1(6) mixture in which FA 18:1(9) ranged from 1-200 µM, with FA 18:1(6) kept at 5 µM using direct infusion. While FA 18:1 and FA 18:1(9) relative abundance varied, that of FA 18:1(6) stayed at 1, which was consistent with experiment results, and further validated the quantification feasibility of our duplex AMPE-tags.

(a)

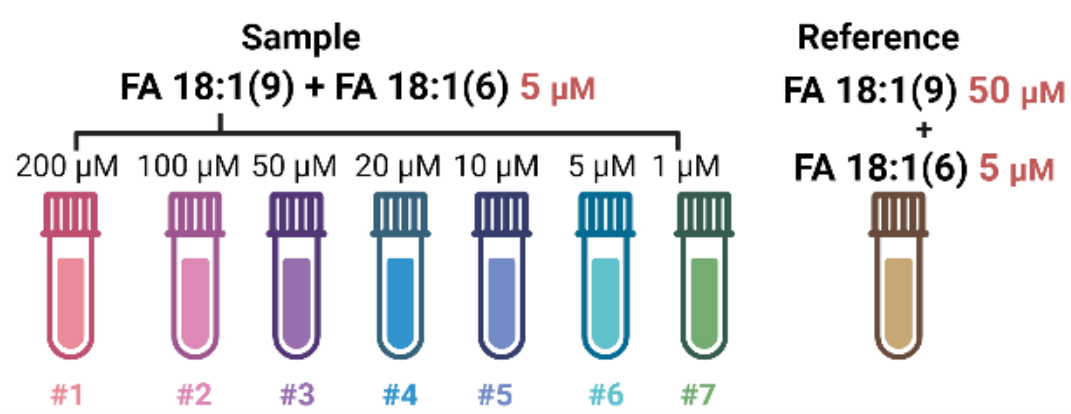

Ratio in sample vs. reference	#1	#2	#3	#4	#5	#6	#7
FA 18:1	3.73	1.91	1	0.45	0.27	0.18	0.11
FA 18:1(9)	4	2	1	0.4	0.2	0.1	0.02
FA 18:1(6)	1	1	1	1	1	1	1

(b)

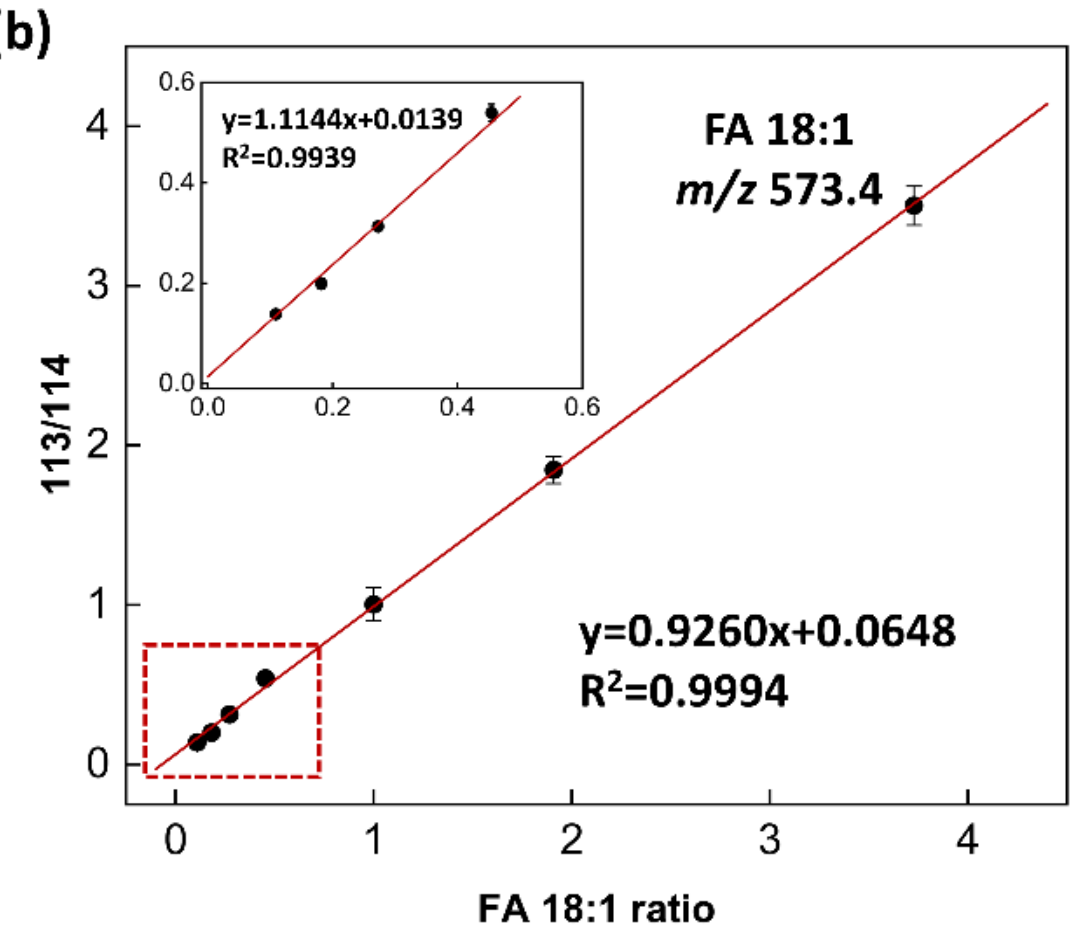

(c)

Calibration Curves for Each Diagnostic Ions

Quantification species	Diagnostic ion	Calibration curve	R^2
FA 18:1(9)	*m/z* 401.3	y=0.9514x-0.0032	0.9981
	m/z 431.3	y=0.9245x-0.0038	0.9989
FA 18:1(6)	*m/z* 389.3	avg. 113/114 = 0.9567	-
	m/z 443.3	avg. 113/114 = 0.9582	-

(d) (e)

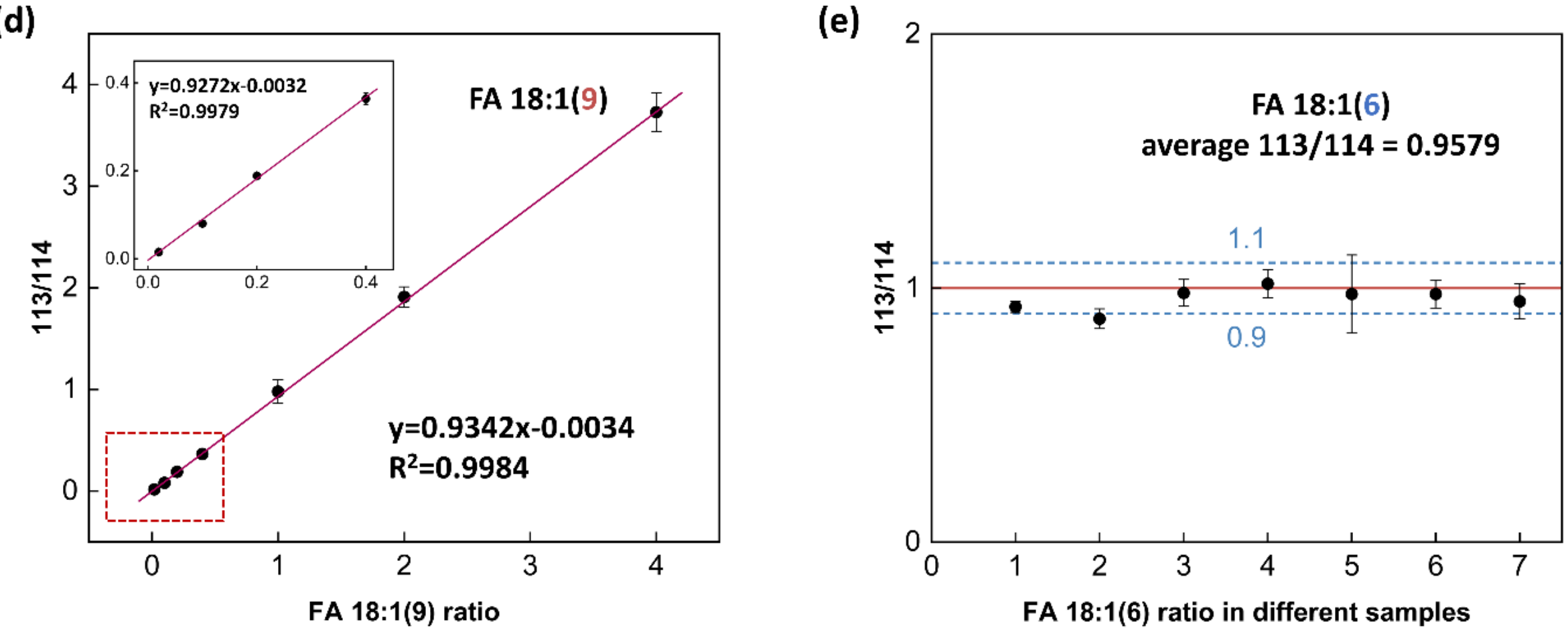

Figure S27. Quantification results of FA 18:1(9)/FA 18:1(6) mixure with FA 18:1(6) concentration keeping consistent. While calibration curves were still done for FA 18:1 and FA 18:1(9) with different ratios, the results showed that the concentration of FA 18:1(6) was the same between samples and references.

S8.4 Quantification of FA 18:2(9,12)

A polyunsaturated FA was chosen for validation as well. FA 18:2(9,12) solutions were made from 1-200 µM with serial dilution, and references of 50 µM were separately reacted and mixed. Two pairs of diagnostic ions were found, with *m/z* 339 and *m/z* 431 for the C=C bond at Δ9 and *m/z* 359 and *m/z* 471 for Δ12. Calibration curves could be achieved

for the PB product and all four diagnostic ions, for quantification at the total level and the isomeric level, respectively. The calibration curve of *m/z* 471 was slightly deviated from linear, as this diagnostic ion was surrounded by a lot of other peaks, and isolation of this one could be distracted.

(a)

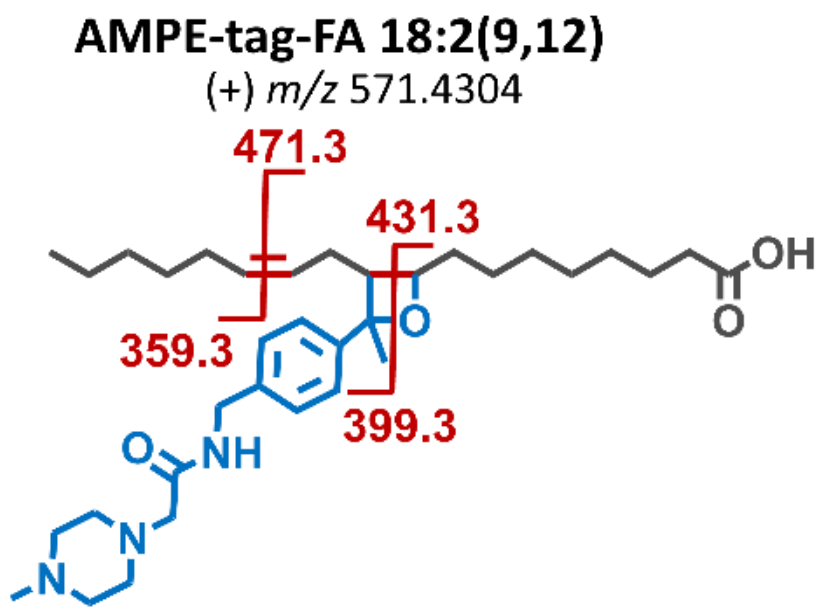

Sample: 1-200 µM, FA 18:2(9,12)
Reference: 50 µM, FA 18:2(9,12)

Ratio in sample vs. reference	#1	#2	#3	#4	#5	#6	#7
**FA 18:2(9,12)**	4	2	1	0.4	0.2	0.1	0.02

(b)

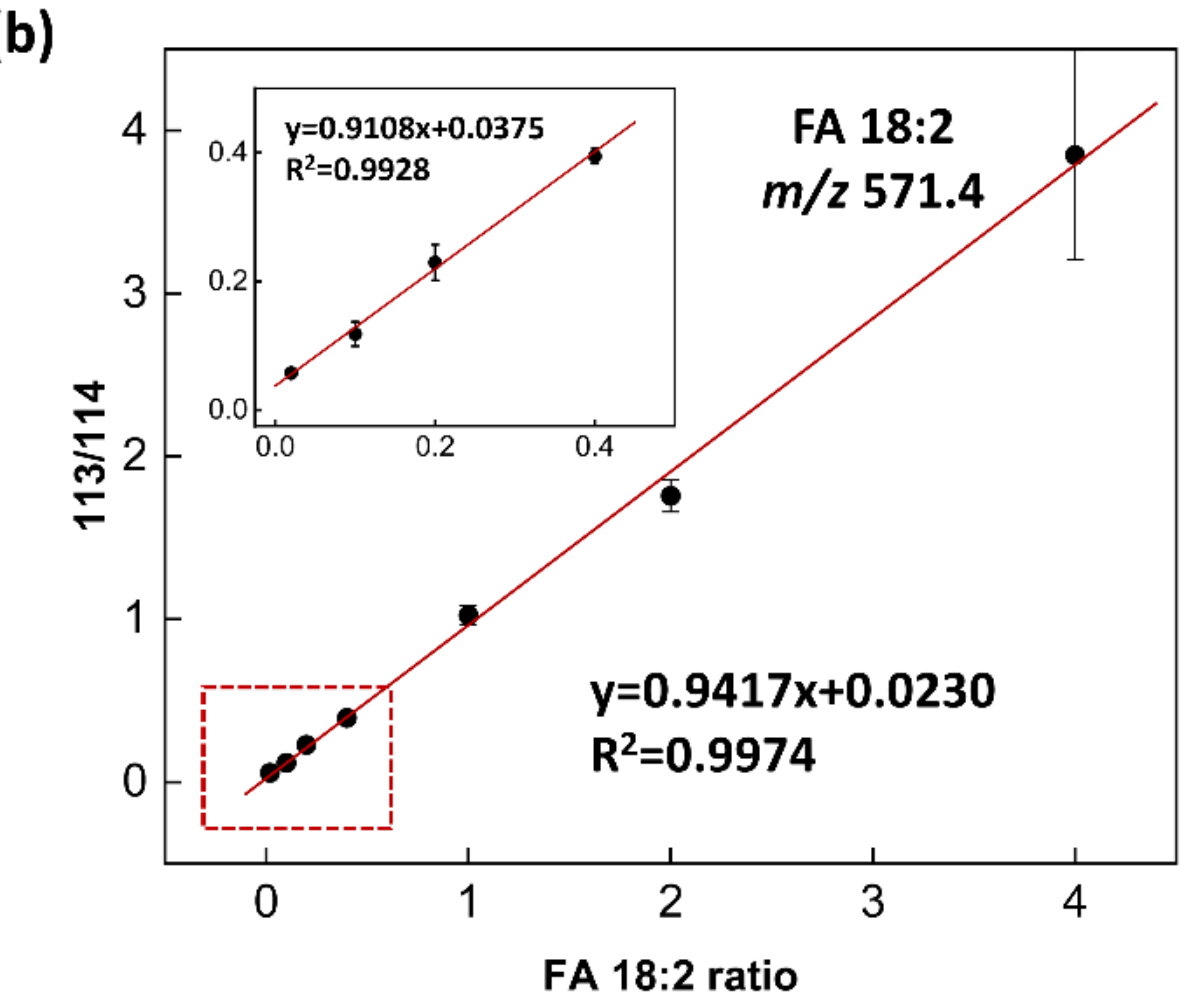

(c)

Calibration Curves for Each Diagnostic Ions

Quantification species	Diagnostic ion	Calibration curve	R^2
FA 18:2(9,12)	***m/z* 399.3**	y=0.9084x-0.0007	0.9982
	***m/z* 431.3**	y=0.9261x+0.0005	0.9981
FA 18:2(9,12)	***m/z* 359.3**	y=0.9296x+0.0017	0.9965
	***m/z* 471.3**	y=0.9465x-0.0017	0.9929

(d)

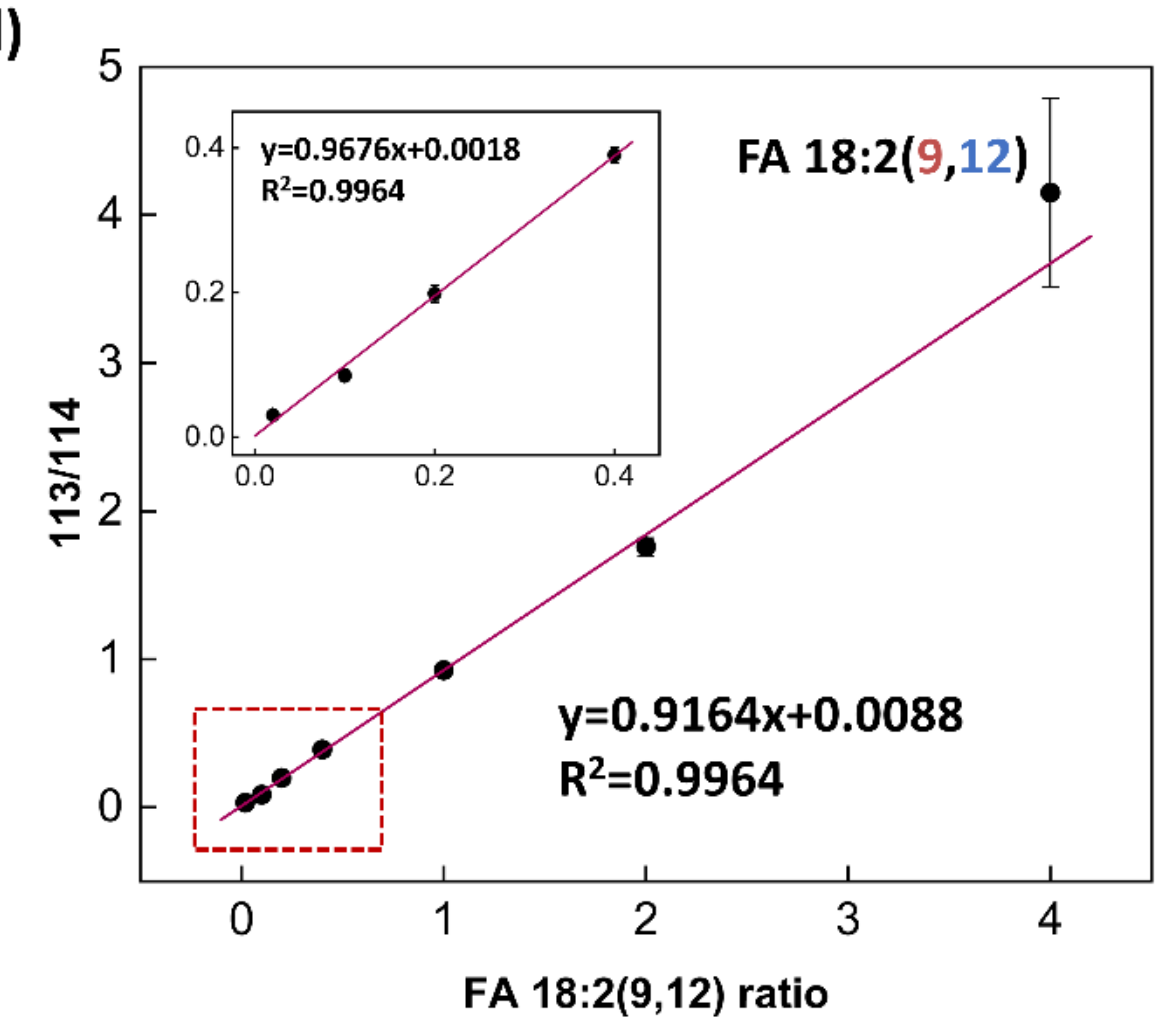

Figure S28. Quantification results of FA 18:2(9,12). Calibration curves were accomplished for the PB product and all diagnostic ions, showing the quantification feasibility of the AMPE-tag for polyunsaturated FAs.

S8.5 Quantification of FAEE 18:1(9)/FAEE 18:1(11)

Two FAEE isomers, FAEE 18:1(9) and FAEE18:1(11), were investigated using direct infusion. The *m/z* of the PB products and diagnostic ions were labeled in Fig.S29a. Calibration curves were done in a similar way to FAs, and quantification was successfully achieved for this kind of FA derivatives.

(a)

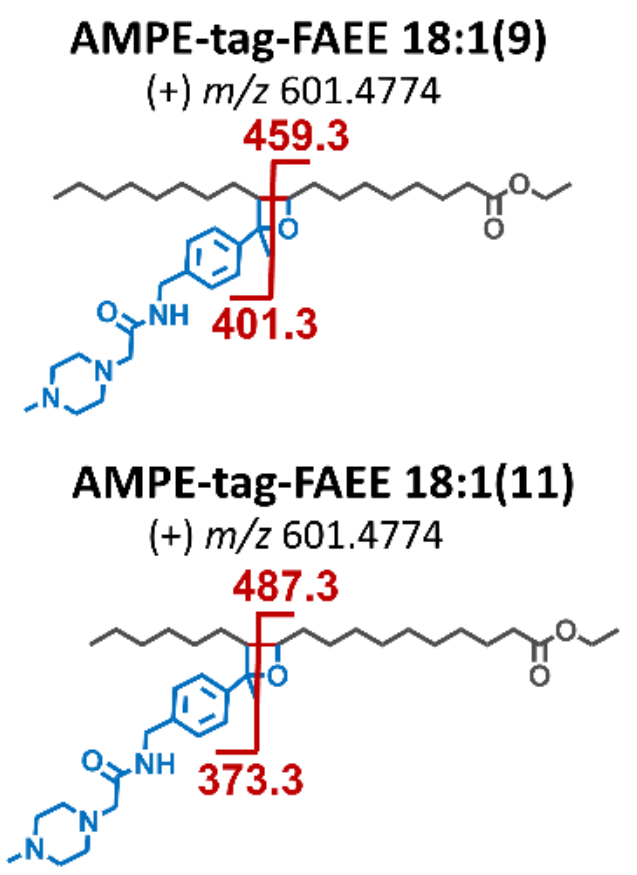

(b)

Sample: 1-200 µM, FAEE 18:1(9)/CE 18:1(11) 1:1
Reference: 50 µM, FAEE 18:1(9)/CE 18:1(11) 1:1

Ratio in sample vs. reference	#1	#2	#3	#4	#5	#6	#7
**FAEE 18:1**	4	2	1	0.4	0.2	0.1	0.02
**FAEE 18:1(9)**	4	2	1	0.4	0.2	0.1	0.02
**FAEE 18:1(11)**	4	2	1	0.4	0.2	0.1	0.02

(c)

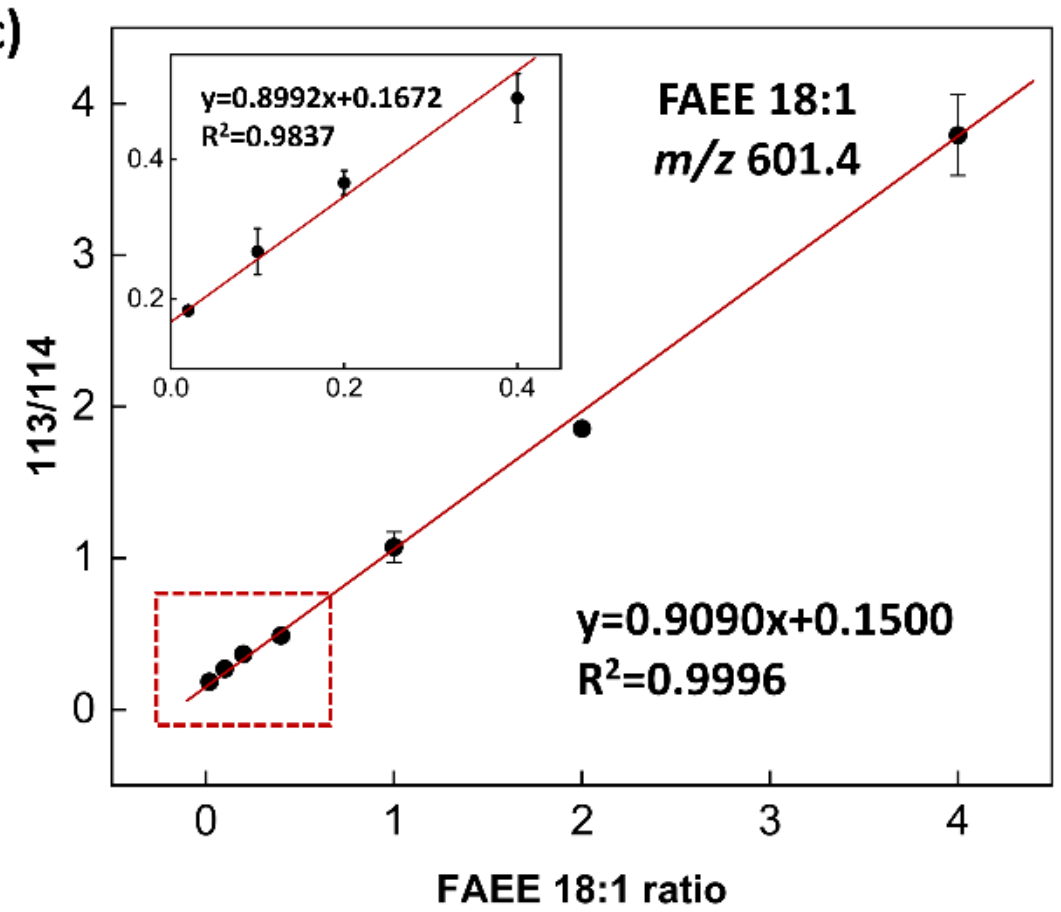

(d)

Calibration Curves for Each Diagnostic Ions

Quantification species	Diagnostic ion	Calibration curve	R^2
FAEE 18:1(9)	***m/z*** **401.3**	y=0.9362x-0.0003	0.9964
	m/z **459.3**	y=0.9244x+0.0021	0.9991
FAEE 18:1(11)	***m/z*** **373.3**	y=0.9103x-0.0011	0.9986
	m/z **487.3**	y=0.9112x+0.0012	0.9992

(e) (f)

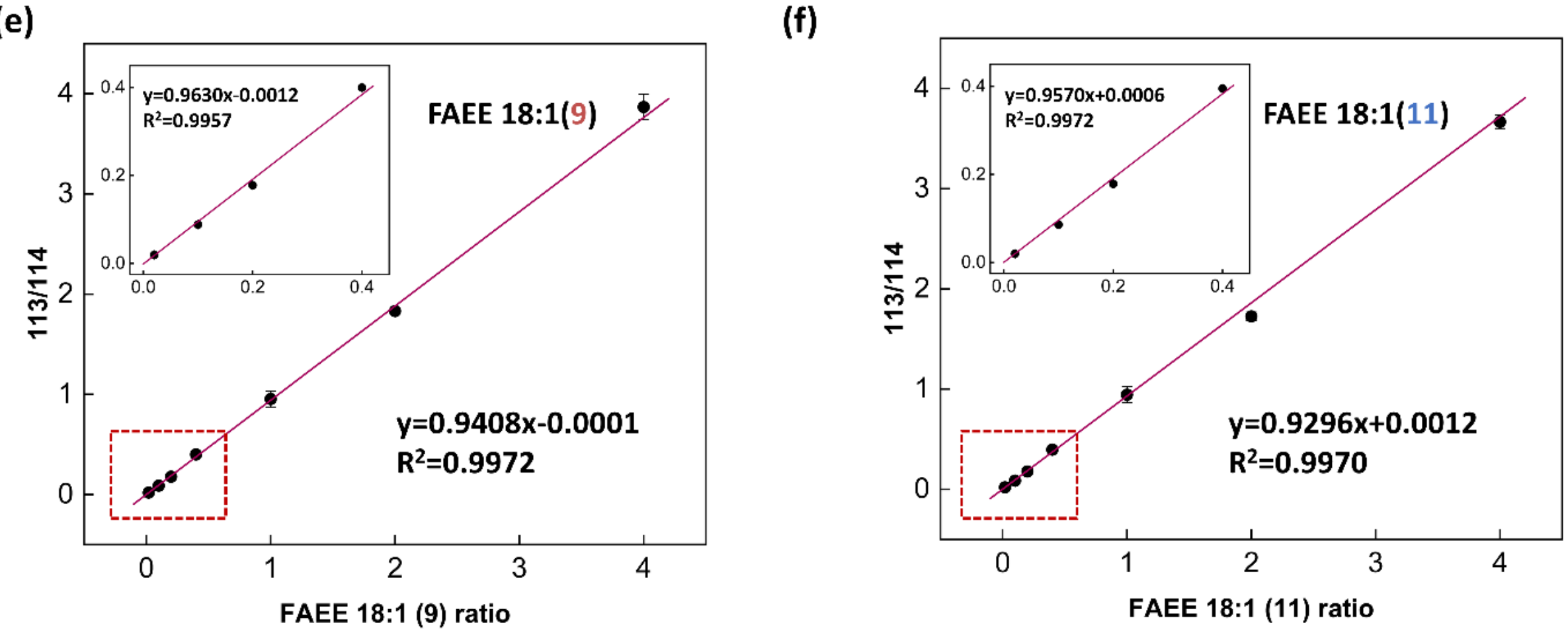

Figure S29. Quantification results of FAEE 18:1(9)/FAEE 18:1(11) 1:1 mixture. Calibration curves were accomplished for the PB product and all diagnostic ions, showing the quantification feasibility of the AMPE-tag for FAEEs.

S8.6 Quantification of CE 18:1(9)/CE 18:1(11)

Two CE isomers, CE 18:1(9) and CE18:1(11), were investigated using direct infusion. The *m/z* of the PB products and diagnostic ions were labeled in Fig.S30a. CE has three diagnostic ions, two from the cleavage of the oxetane ring with fatty acyl ring attached to the cholestereol and one from the fatty acyl ring released from the cholestereol. Calibration curves were done in a similar way to FAs, and quantification was successfully achieved for CE lipids.

(a)

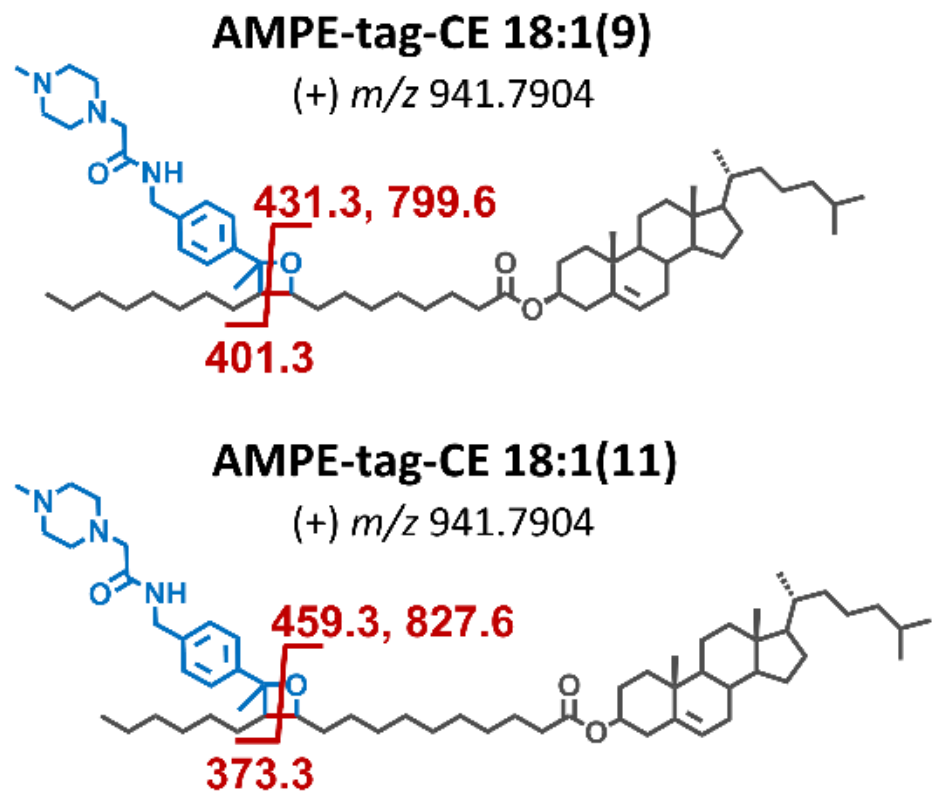

(b)

Sample: 1-200 μM, CE 18:1(9)/CE 18:1(11) 1:1
Reference: 50 μM, CE 18:1(9)/CE 18:1(11) 1:1

Ratio in sample vs. reference	#1	#2	#3	#4	#5	#6	#7
CE 18:1	4	2	1	0.4	0.2	0.1	0.02
CE 18:1(9)	4	2	1	0.4	0.2	0.1	0.02
CE 18:1(11)	4	2	1	0.4	0.2	0.1	0.02

(c)

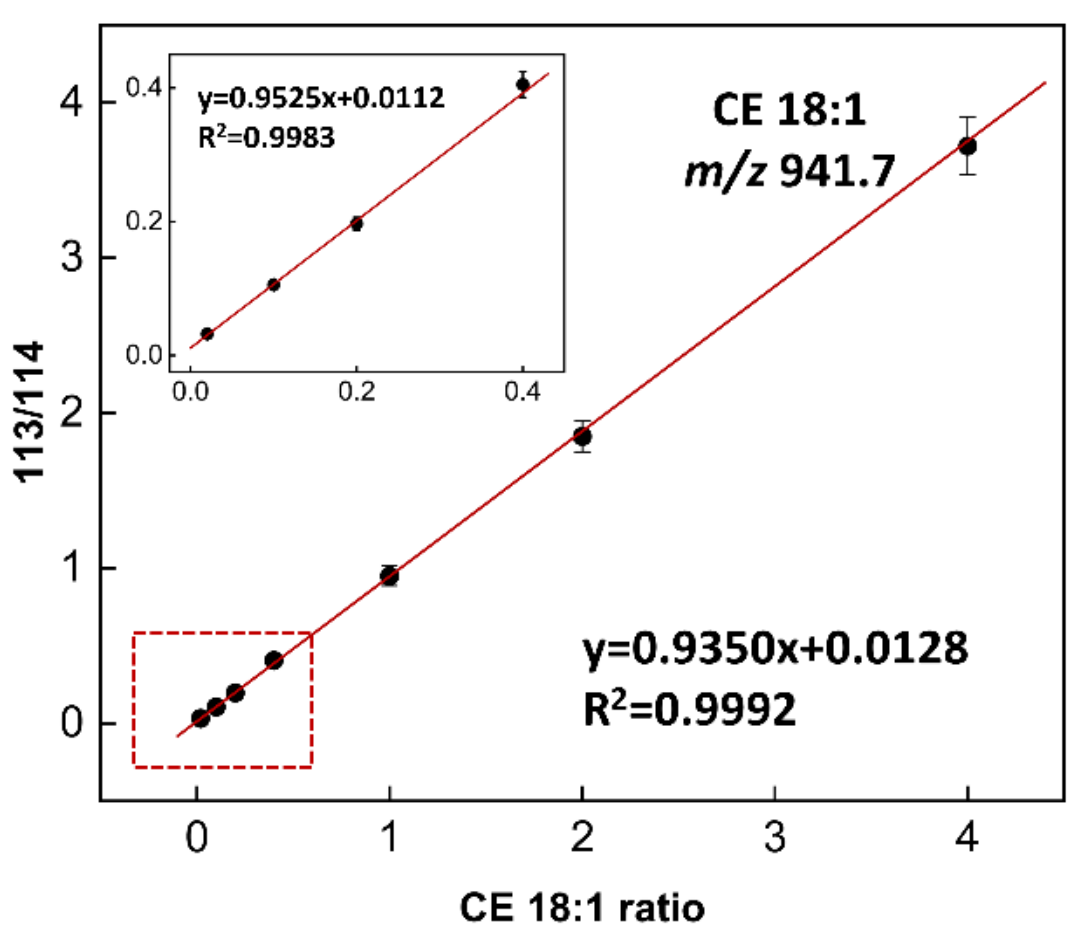

(d)

Calibration Curves for Each Diagnostic Ions

Quantification species	Diagnostic ion	Calibration curve	R^2
CE 18:1(9)	*m/z* 401.3	y=0.9964x-0.0025	0.9995
	m/z 431.3	y=1.0123x-0.0108	0.9999
	m/z 799.6	y=0.9757x+0.0057	0.9993
CE 18:1(11)	*m/z* 373.3	y=0.9809x+0.0060	0.9989
	m/z 459.3	y=0.9550x-0.0017	0.9986
	m/z 827.6	y=1.0089x-0.0086	0.9992

(e)

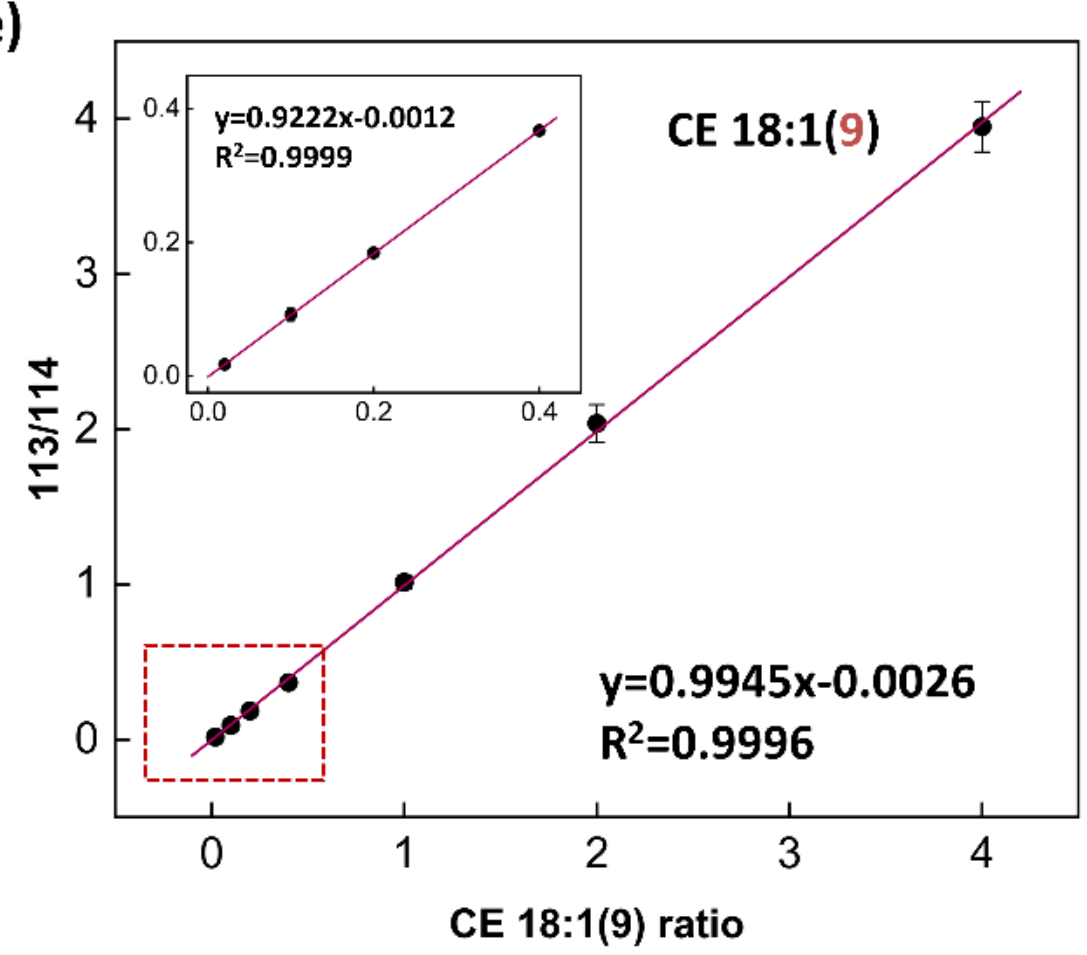

(f)

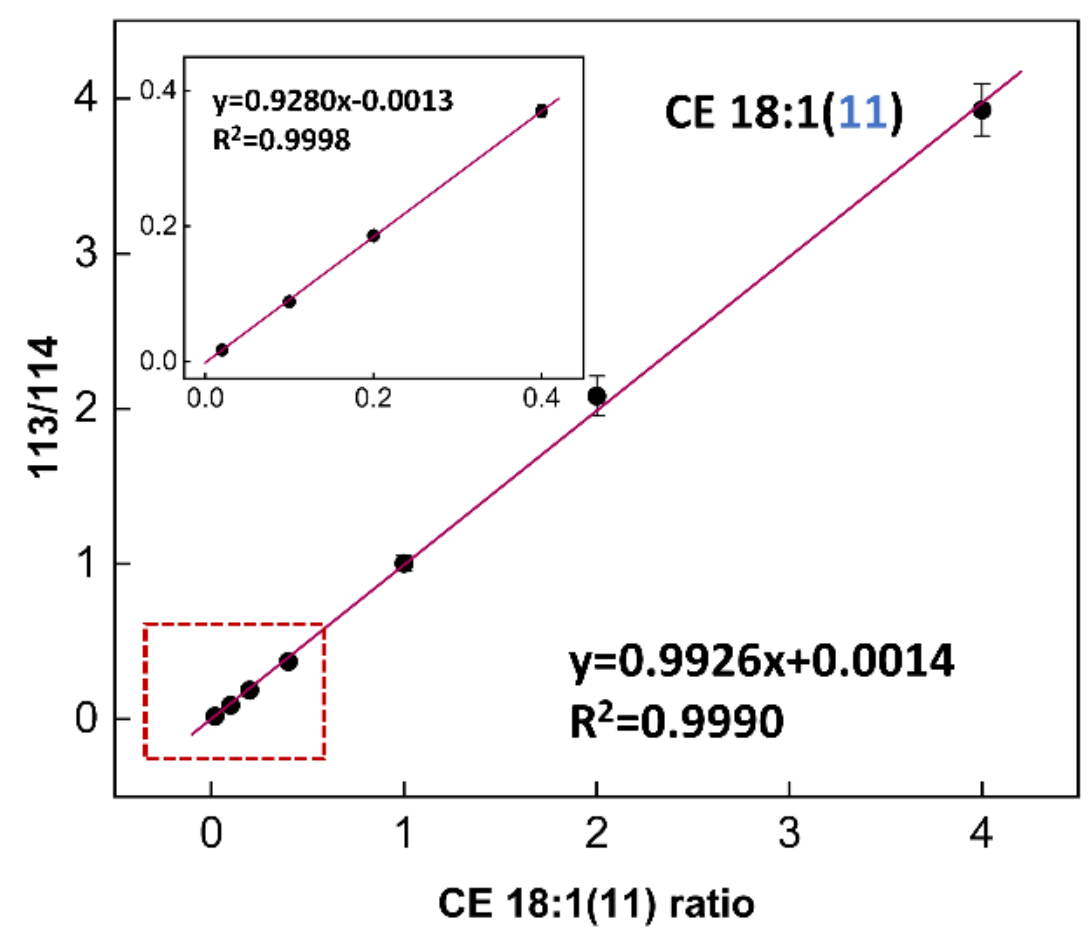

Figure S30. Quantification results of CE 18:1(9)/CE 18:1(11) 1:1 mixture. Calibration curves were accomplished for the PB product and all diagnostic ions, showing the quantification feasibility of the AMPE-tag for CEs.

S9. Limit of detection

The limit of detection of this method was evaluated with FA 18:1(9) and AMPE-tag i114 using direct infusion. The PB product at *m/z* 573.4 was found, although not obvious due to the very high peak from excess AMPE-tag. Diagnostic ions at *m/z* 401.3 and *m/z* 431.3 upon CID, and the mass reporter ions at *m/z* 114.1 at both MS^2 and MS^3 upon HCD were found with a good signal-to-noise ratio with FA 18:1(9) concentration at 50 nM.

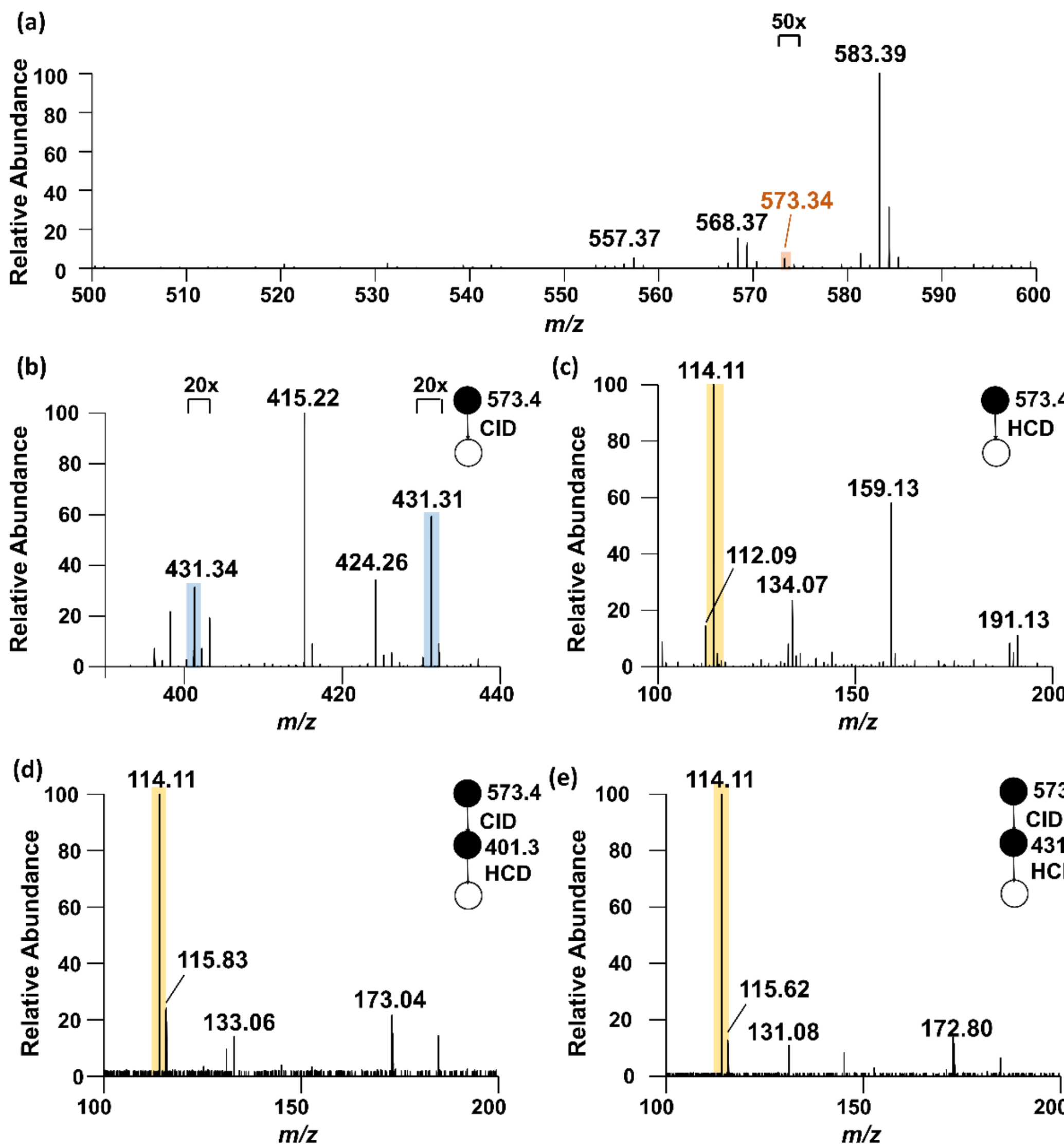

Figure S31. The limit of detection was evaluated by FA 18:1(9) and AMPE-tag i114. (a) Full MS after reaction, the PB product was labeled in organge; (b) diagnostic ions at *m/z* 401.3 and *m/z* 431.3 after CID, which were labeled in blue; (c) mass reporter ions at *m/z* 114.1 from the PB product at MS^2, indicating the relative abundance of FAs at the toatal level; (d-e) mass reporter ions at *m/z* 114.1 from the two diagnostic ions at MS^3, indicating the relative abundance of FA 18:1(9) at the isomeric level.

S10. Artificial FA mixture analysis by direct infusion

A mixture of six FAs was used to resemble the FA complex, and it was analyzed both by direct infusion and HPLC-MS. The direct infusion analysis result is shown below.

As a large quantity of isobaric mass tag was added, its peak dominated the spectra. The product peaks were observed, though at low intensity (Fig. S32a). The quantification result was not accurate at MS^2 due to the interference from adjacent peaks (Fig. S32c). At MS^3, the quantification result was acceptable (0.9~1.1 of the theoretical value), but a few data points were still out of range. The results corresponded to the observation above, revealed the accuracy of quantification at MS^3, and further emphasized the importance of separation and purification prior to MS analysis.

(a)

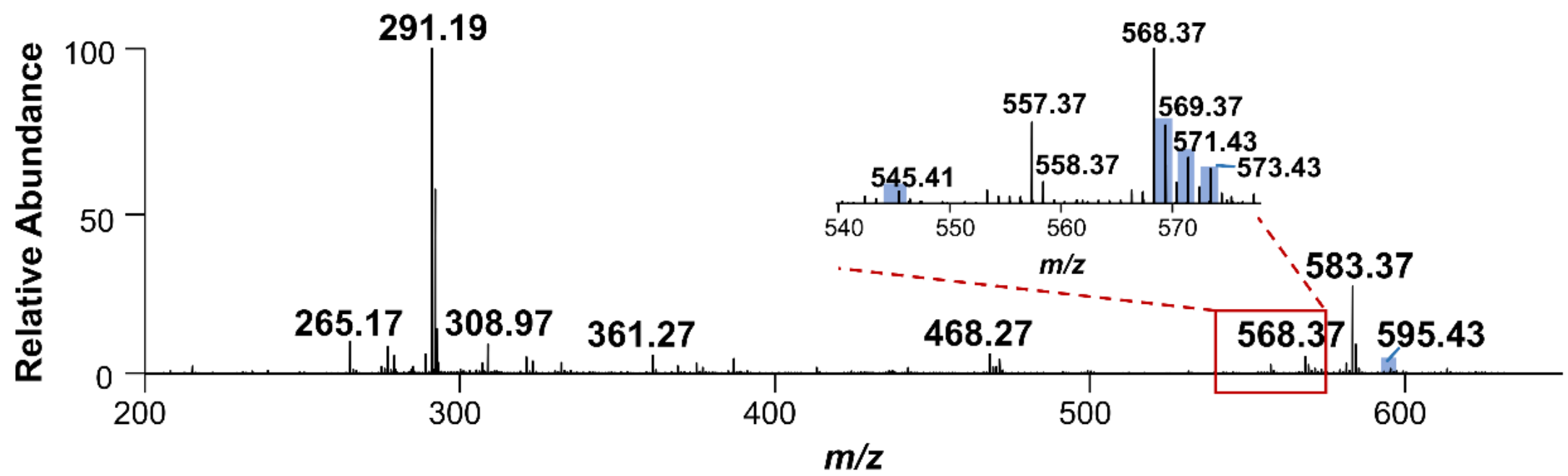

(b)

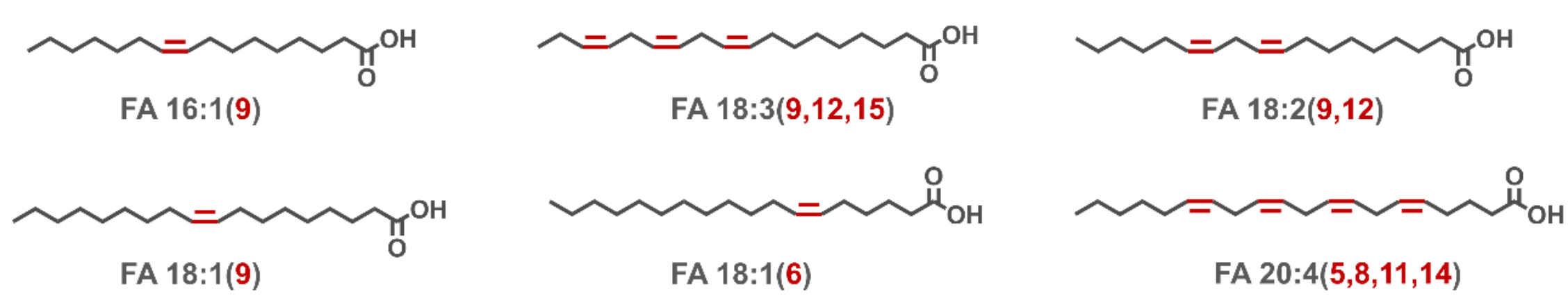

(c)

Fatty acid	*m/z* of PB product	113/114 from PB product	*m/z* of diagnostic ion	113/114 from diagnostic ion	Avg. of 113/114 from diagnostic ions
FA 16:1(9)	545.4	1.62	373.3(Δ9)<br>431.3(Δ9)	1.84<br>1.84	1.84
FA 18:1(9)	573.4	1.68	401.3(Δ9)<br>431.3(Δ9)	1.80<br>1.81	1.80
FA 18:1(6)	573.4	1.68	389.2(Δ6)<br>443.3(Δ6)	1.84<br>1.81	1.82
FA 18:2(9,12)	571.4	1.60	359.2(Δ12)<br>471.3(Δ12)<br>399.3(Δ9)<br>431.3(Δ9)	1.83<br>1.81<br>1.82<br>1.86	1.83
FA 18:3(9,12,15)	569.4	1.46	511.3(Δ15)<br>317.2(Δ15)<br>357.2(Δ12)<br>471.3(Δ12)<br>397.3(Δ9)<br>431.3(Δ9)	1.84<br>1.97<br>1.93<br>1.75<br>1.89<br>1.85	1.87
FA 20:4(5,8,11,14)	595.4	1.63	359.2(Δ14)<br>495.3(Δ14)<br>399.3(Δ11)<br>455.3(Δ11)<br>439.3(Δ8)<br>415.2(Δ8)<br>479.4(Δ5)<br>375.2(Δ5)	1.87<br>1.70<br>1.83<br>1.81<br>1.87<br>1.84<br>1.75<br>1.84	1.81

Figure S32. The result of artificial FA mixture analysis by direct infusion. (a) Full MS spectra of the reaction solution after UV irradiation; (b) structures of the six FAs consisting of the artificial mixture; (c) summary of quantification results at MS^2 (FAs at the total level) and MS^3 (FAs at the isomeric level) by direct infusion.

S11. Structural and quantitative analysis of FAs and FA isomers in murine VAT

S11.1 TAGs detected from lipid extract of murine VAT

The lipid extract from mouse VAT was analyzed by HPLC-MS/MS (ACN/H_2O/IPA) using DDA. Annotation was done manually. The majority of lipids from VAT were TAGs (or TGs). Altogether, 26 TAGs and 1 DAG (or DG) were observed. Details are summarized in the following table.

Table S2. TAGs observed from the lipid extract of mouse VAT. *m/z* observed, RT, theoretical *m/z*, TAG identification, format of ions detected, and examples of TAGs with possible fatty acyl chain combinations were provided. The fatty acyl chain combination was provided by fragments in tandem MS, and up to three examples were shown.

m/z	RT (min)	theoretical *m/z*	identification	format	note		
866.71174	14.649	866.7232	TG 52:7	$[M+NH_4]^+$	TG 14:0_18:2_20:5	TG 16:1_18:3_18:3	TG 16:1_16:1_20:5
816.69686	14.773	816.7076	TG 48:4	$[M+NH_4]^+$	TG 18:2_16:1_14:1	TG 12:0_18:2_18:2	TG 14:0_16:2_18:2
892.72498	14.773	892.7389	TG 54:8	$[M+NH_4]^+$	TG 16:2_18:1_20:5	TG 18:2_18:3_18:3	TG 18:2_18:2_18:4
842.71194	14.834	842.7232	TG 50:5	$[M+NH_4]^+$	TG 14:0_18:2_18:3	TG 14:1_18:2_18:2	TG 16:1_16:1_18:3
868.72719	14.834	868.7389	TG 52:6	$[M+NH_4]^+$	TG 16:1_18:2_18:3	TG 16:2_18:2_18:2	TG 16:1_18:1_18:4
894.74179	14.895	894.7545	TG 54:7	$[M+NH_4]^+$	TG 18:2_18:2_18:3	TG 18:1_18:2_18:4	
944.75671	14.895	944.7702	TG 58:10	$[M+NH_4]^+$	TG 18:2_18:2_22:6		
818.71233	15.018	818.7232	TG 48:3	$[M+NH_4]^+$	TG 16:1_16:1_16:1	TG 14:0_16:1_18:2	TG 12:0_18:2_18:2
844.72739	15.079	844.7389	TG 50:4	$[M+NH_4]^+$	TG 16:1_16:1_18:2	TG 14:0_18:2_18:2	
870.74264	15.139	870.7545	TG 52:5	$[M+NH_4]^+$	TG 16:1_18:2_18:2	TG 16:0_18:2_18:3	TG 16:0_16:0_20:5
896.75714	15.139	896.7702	TG 54:6	$[M+NH_4]^+$	TG 18:0_18:2_18:4	TG 18:2_18:2_18:2	
946.77163	15.139	946.7858	TG 58:9	$[M+NH_4]^+$	TG_18:1_18:2_22:6	TG 18:1_18:1_22:7	

858.74331	15.262	858.7545	TG 51:4	$[M+NH_4]^+$	TG 15:1_18:1_18:2	TG 16:1_17:1_18:2	TG 15:0_16:1_18:3
820.72858	15.383	820.7389	TG 48:2	$[M+NH_4]^+$	TG 16:0_16:1_16:1	TG 14:0_16:0_18:2	TG 14:0_16:1_18:1
822.73528	15.383	822.7545	TG 48:1	$[M+NH_4]^+$	TG 16:0_16:0_16:1	TG 14:0_16:0_18:1	
846.74338	15.383	846.7545	TG 50:3	$[M+NH_4]^+$	TG 16:1_16:1_18:1	TG 14:0_18:1_18:2	TG 16:0_16:1_18:2
575.49596	15.414	575.467	DG 33:4	$[M+H]^+$	DG 16:0_17:4		
848.75089	15.414	848.7702	TG 50:2	$[M+NH_4]^+$	TG 16:0_16:1_18:1	TG 14:0_18:1_18:1	TG 16:0_16:0_18:2
872.75827	15.414	872.7702	TG 52:4	$[M+NH_4]^+$	TG 16:0_18:2_18:2	TG 16:1_18:1_18:2	
898.77231	15.414	898.7858	TG 54:5	$[M+NH_4]^+$	TG 18:1_18:2_18:2	TG 18:1_18:2_18:3	TG 18:0_18:2_18:3
948.78655	15.414	948.8015	TG 58:8	$[M+NH_4]^+$	TG 18:1_18:1_22:6	TG 18:1:18:2_22:5	
862.76771	15.536	862.7858	TG 51:2	$[M+NH_4]^+$	TG 15:0_18:1_18:1	TG 16:0_17:0_18:2	TG 16:1_17:1_18:0
860.7592	15.566	860.7702	TG 51:3	$[M+NH_4]^+$	TG 15:0_18:1_18:2	TG 16:0_17:1_18:2	TG 16:1_17:1_18:1
886.7737	15.566	886.7858	TG 53:4	$[M+NH_4]^+$	TG 17:1_18:1_18:2	TG 17:0_18:2_18:2	TG 16:1_18:2_19:1
888.78796	15.566	888.8015	TG 53:3	$[M+NH_4]^+$	TG 17:0_18:1_18:2	TG 16:0_18:2_19:1	TG 15:0_18:2_20:1
892.81928	15.657	892.8328	TG 53:1	$[M+NH_4]^+$	TG 17:0_18:0_18:1	TG 16:0_18:0_19:1	TG 15:0_18:0_20:1
900.78719	15.657	900.8015	TG 54:4	$[M+NH_4]^+$	TG 18:1_18:1_18:2	TG 18:0_18:1_18:3	

S11.2 FAs detected from hydrolysis of TAGs in murine VAT

TAGs extracted from VAT then underwent saponification to yield free FAs, as described in the Method and Materials section. Hydrolyzed FAs were analyzed by HPLC-MS (MeOH/H_2O), and the result was summarized in Table S3.

Table S3. FAs observed after hydrolysis of TAGs extracted from murine VAT. FA annotation, *m/z* observed, theoretical *m/z*, RT, and format of ions were provided.

FA	*m/z*	theoretical *m/z*	RT (min)	format
FA 14:1	225.187	225.186	21.07	$[M\text{-}H]^-$
FA 14:0	227.2026	227.2017	24.32	$[M\text{-}H]^-$
FA 15:0	241.2184	241.2173	26.52	$[M\text{-}H]^-$
FA 16:2	251.203	251.2017	22.79	$[M\text{-}H]^-$
FA 16:1	253.2186	253.2173	25.25	$[M\text{-}H]^-$
FA 16:0	255.2341	255.233	28.61	$[M\text{-}H]^-$
FA 17:2	265.2185	265.2173	24.81	$[M\text{-}H]^-$
FA 17:1	267.2343	267.233	27.29	$[M\text{-}H]^-$
FA 17:0	269.2501	269.2486	30.61	$[M\text{-}H]^-$
FA 18:3	277.2192	277.2173	24.62	$[M\text{-}H]^-$
FA 18:2	279.2342	279.233	26.76	$[M\text{-}H]^-$
FA 18:1	281.2488	281.2486	29.29	$[M\text{-}H]^-$
FA 18:0	283.2641	283.2643	32.39	[M-H]-
FA 19:2	293.2492	293.2486	28.69	[M-H]-
FA 19:1	295.2649	295.2643	31.14	[M-H]-
FA 20:5	301.2181	301.2173	24.46	$[M\text{-}H]^-$
FA 20:4	303.2338	303.233	26.52	$[M\text{-}H]^-$
FA 20:3	305.2493	305.2486	28.25	$[M\text{-}H]^-$
FA 20:2	307.265	307.2643	30.47	$[M\text{-}H]^-$
FA 20:1	309.2807	309.2799	32.68	$[M\text{-}H]^-$
FA 22:6	327.2337	327.233	26.47	$[M\text{-}H]^-$
FA 22:5	329.2493	329.2486	26.47	$[M\text{-}H]^-$
FA 22:4	331.2649	331.2643	29.64	$[M\text{-}H]^-$
FA 21:1	323.2934	323.2956	31.03	$[M\text{-}H]^-$

FA 24:6	355.265	355.2643	29.27	$[M-H]^-$
FA 24:5	357.2802	357.2799	31.01	$[M-H]^-$

S11.3 FA C=C bond positional isomers identified within FAs in TAGs of murine VAT

Isobaric AMPE-tag i113 and i114 were separately attached to FAs. The reaction complex was analyzed, and FA-tag derivatives were found. Further analysis was conducted to determine FA C=C bond positional isomers and quantify FAs at both the total level and the isomeric level.

FA-tag products observed and FA isomers identified, were listed in Table S4.

Table S4. FA-tag products observed after the PB reaction with AMPE-tag, and FA C=C bond positional isomers detected. FA-tag *m/z*, RT; diagnostic ion *m/z*, RT, and isomer identification were provided. Some diagnostic ions were of too low intensity to generate mass reporter ions at MS^3 for quantification. FAs selected for quantification are shown in bold, indicating strong diagnostic ion intensities suitable for accurate quantification.

**FA**	**FA_tag *m/z* theoretical**	**FA_tag *m/z* experimental**	**FA_tag RT (min)**	**diagnostic ion *m/z* theoretical**	**diagnostic ions *m/z* experimental**	**diagnostic ion RT (min)**	**identification result**
FA 14:1	517.3829	517.3822	13.4	431.3098	431.3084 (Δ9)	13.52	**FA 14:1 (7)** **FA 14:1 (9)**
				345.272	345.2730 (Δ9)	13.52	
				403.2785	403.2772 (Δ7)	13.52	
				373.3043	373.3034 (Δ7)	13.64	
FA 16:2	543.3986	543.3977	13.75	359.2886	359.2876 (Δ7, **Δ10**)	14	**FA 16:2 (7, 10)** **FA 16:2 (9, 12)** FA 16:2 (10, 13)
				443.3098	443.3082 (Δ7, **Δ10**)	14	
				399.3199	399.3186 (**Δ7**, Δ10)	14.22	
				403.2785	403.2773 (**Δ7**, Δ10)	13.89	
				331.2573	331.2665 (Δ9, **Δ12**)	14	
				471.3411	471.3399 (Δ9, **Δ12**)	14	
				371.2886	371.2876 (**Δ9**, Δ12)	14	
				431.3098	431.3084 (**Δ9**, Δ12)	13.89	
				485.3567	485.3552 (Δ10, **Δ13**)	14	
				317.2417	317.2407 (Δ10, **Δ13**)	13.66	
				445.3254	445.3244 (**Δ10**, Δ13)	14	
				357.273	-	-	
FA 16:1	545.4142	545.4132	14.09	373.3043	373.3032 (Δ9)	14.6	**FA 16:1 (7)** **FA 16:1 (9)** FA 16:1 (10) FA 16:1 (11)
				431.3098	431.3085 (Δ9)	14.36	
				403.2785	403.2774 (Δ7)	14.48	
				401.3356	401.3343 (Δ7)	14.71	
				445.3254	445.3242 (Δ10)	14.48	
				359.2886	359.2879 (Δ10)	14.48	
				459.3411	459.3393 (Δ11)	14.36	

				345.273	345.2717 (Δ11)	14.48	
FA 17:2	557.4142	557.4135	14.79	457.3254	457.3244 (Δ8, **Δ11**)	15.08	FA 17:2 (8, 11)
				359.2886	-	-	
				417.2941	417.2927 (**Δ8**, Δ11)	14.84	
				399.3199	-	-	
FA 17:1	559.4299	559.4293	15.54	403.2785	403.2776 (Δ7)	15.24	**FA 17:1 (7)**<br>**FA 17:1 (8)**<br>**FA 17:1 (9)**<br>FA 17:1 (10)<br>**FA 17:1 (11)**
				415.3512	415.3501 (Δ7)	15.62	
				417.2941	417.293 (Δ8)	16.3	
				401.3356	401.3343 (Δ8)	15.36	
				431.3098	431.3087 (Δ9)	15.11	
				387.3199	387.3190 (Δ9)	15.24	
				445.3254	445.3240 (Δ10)	14.4	
				373.3043	373.3037 (Δ10)	15.24	
				459.3411	459.3402 (Δ11)	14.98	
				359.2886	359.2878 (Δ11)	15.11	
FA 18:3	569.4142	569.4123	14.09	431.3098	431.3088 (**Δ9**, Δ12, Δ15)	14.3	**FA 18:3 (6, 9, 12)**<br>**FA 18:3 (9, 12, 15)**
				397.3043	397.3031 (**Δ9**, Δ12, Δ15)	14.65	
				471.3411	471.3398 (Δ9, **Δ12**, Δ15)	14.65	
				357.273	357.2721 (Δ9, **Δ12**, Δ15)	14.53	
				511.3724	511.3708 (Δ9, Δ12, **Δ15**)	14.65	
				317.2417	317.2407 (Δ9, Δ12, **Δ15**)	14.65	
				389.2628	389.2620 (**Δ6**, Δ9, Δ12)	14.53	
				439.3512	439.3500 (**Δ6**, Δ9, Δ12)	15.13	
				429.2941	429.2931 (Δ6, **Δ9**, Δ12)	15	
				399.3199	399.3186 (Δ6, **Δ9**, Δ12)	14.88	
				469.3254	469.3243 (Δ6, Δ9, **Δ12**)	15.25	
				359.2886	359.2880 (Δ6, Δ9, **Δ12**)	14.53	
FA 18:2	571.4299	571.4279	14.67	359.2886	359.2879 (Δ9, **Δ12**)	15.14	**FA 18:2 (9, 12)**
				471.3411	471.3399 (Δ9, **Δ12**)	15.26	
				399.3199	399.3189 (**Δ9**, Δ12)	15.52	
				431.3098	431.3087 (**Δ9**, Δ12)	15.01	
FA 18:1	573.4455	573.4457	15.41	429.3669	429.3654 (Δ7)	16.92	FA 18:1 (7)<br>FA 18:1 (8)<br>**FA 18:1 (9)**<br>**FA 18:1 (11)**
				403.2785	403.2769 (Δ7)	16.35	
				415.3512	415.3499 (Δ8)	16.92	
				417.2941	417.2936 (Δ8)	16.21	

				431.3098	431.3087 (Δ9)	16.07	**FA 18:1 (12)**
				401.3356	401.3346 (Δ9)	16.63	
				387.3199	-	-	
				445.3254	445.3246 (Δ10)	17.06	
				373.3043	373.3034 (Δ11)	16.21	
				459.3411	459. 3403 (Δ11)	15.93	
				359.2886	359.2879 (Δ12)	15.27	
				473.3567	-	-	
FA 19:2	585.4455	585.443	14.79	445.3254	445.3244 (**Δ10**, Δ13)	15.1	**FA 19:2 (9, 12)<br>FA 19:2 (10, 13)**
				399.3199	399.3187 (**Δ10**, Δ13)	17.15	
				485.3567	485.3554 (Δ10, **Δ13**)	15.35	
				359.2886	359.2880 (Δ10, **Δ13**)	16.71	
				431.3098	431.3083 (**Δ9**, Δ12)	15.47	
				413.3356	413.3343 (**Δ9**, Δ12)	15.6	
				471.3411	471.3398 (Δ9, **Δ12**)	16.15	
				373.3043	373.3033 (Δ9, **Δ12**)	15.22	
FA 19:1	587.4612	587.4591	16.55	431.3098	431.3083 (Δ9)	17.47	**FA 19:1 (9)<br>FA 19:1 (10)<br>FA 19:1 (11)<br>FA 19:1 (12)<br>FA 19:1 (13)**
				415.3512	415.3497 (Δ9)	16.86	
				445.3254	445.3244 (Δ10)	18.07	
				401.3356	401.3345 (Δ10)	18.53	
				459.3411	459.3401 (Δ11)	17.17	
				387.3199	387.3190 (Δ11)	17.47	
				473.3567	473.3557 (Δ12)	18.07	
				373.3043	373.3042 (Δ12)	18.22	
				487.3724	487.3711 (Δ13)	17.32	
				359.2886	359.2879 (Δ13)	17.32	
FA 20:5	593.4142	593.4121	14.56	375.2472	375.2464 (**Δ5**, Δ8, Δ11, Δ14, Δ17)	14.83	**FA 20:5 (5, 8, 11, 14, 17)**
				477.3669	477.3661 (**Δ5**, Δ8, Δ11, Δ14, Δ17)	15.45	
				415.2785	415.2777 (Δ5, **Δ8**, Δ11, Δ14, Δ17)	14.83	
				437.3356	437.3347 (Δ5, **Δ8**, Δ11, Δ14, Δ17)	15.08	
				455.3098	455.3089 (Δ5, Δ8, **Δ11**, Δ14, Δ17)	14.71	
				397.3043	397.3033 (Δ5, Δ8, **Δ11**, Δ14, Δ17)	14.71	
				357.273	357.2720 (Δ5, Δ8, Δ11, **Δ14**, Δ17)	14.71	
				495.3411	495.3397 (Δ5, Δ8, Δ11, **Δ14**, Δ17)	14.71	
				535.3724	535.3714 (Δ5, Δ8, Δ11, Δ14, **Δ17**)	14.83	

				317.2417	317.2409 (Δ5, Δ8, Δ11, Δ14, **Δ17**)	14.83	
FA 20:4	595.4299	595.4276	15.43	375.2472	375.2462 (**Δ5**, Δ8, Δ11, Δ14)	15.72	**FA 20:4 (5, 8, 11, 14) FA 20:4 (8, 11, 14, 17)**
				479.3825	479.3813 (**Δ5**, Δ8, Δ11, Δ14)	16.59	
				415.2785	415.2775 (Δ5, **Δ8**, Δ11, Δ14)	15.72	
				439.3512	439.3503 (Δ5, **Δ8**, Δ11, Δ14)	16	
				455.3098	455.3087 (Δ5, Δ8, **Δ11**, Δ14)	15.72	
				399.3199	399.3189 (Δ5, Δ8, **Δ11**, Δ14)	15.59	
				495.3411	495.3403 (Δ5, Δ8, Δ11, **Δ14**)	15.34	
				359.2886	359.2881 (Δ5, Δ8, Δ11, **Δ14**)	15.46	
				437.3356	437.3346 (**Δ8**, Δ11, Δ14, Δ17)	15.59	
				417.2941	417.2937 (**Δ8**, Δ11, Δ14, Δ17)	15.09	
				397.3043	397.3026 (Δ8, **Δ11**, Δ14, Δ17)	15.34	
				457.3254	457.3248 (Δ8, **Δ11**, Δ14, Δ17)	15.46	
				357.273	357.2724 (Δ8, Δ11, **Δ14**, Δ17)	15.21	
				497.3567	497.3561 (Δ8, Δ11, **Δ14**, Δ17)	15.34	
				317.2417	317.2410 (Δ8, Δ11, Δ14, **Δ17**)	15.46	
				537.388	537.3868 (Δ8, Δ11, Δ14, **Δ17**)	15.46	
FA 20:3	597.4455	597.4441	15.69	417.2941	417.2931 (**Δ8**, Δ11, Δ14)	16.01	**FA 20:3 (8, 11, 14) FA 20:3 (11, 14, 17)**
				439.3512	439.3499 (**Δ8**, Δ11, Δ14)	16.75	
				457.3254	457.3241 (Δ8, **Δ11**, Δ14)	15.87	
				399.3199	399.3187 (Δ8, **Δ11**, Δ14)	16.3	
				497.3567	497.3555 (Δ8, Δ11, **Δ14**)	16.15	
				359.2886	359.2877 (Δ8, Δ11, **Δ14**)	16.3	
				459.3411	459.3398 (**Δ11**, Δ14, Δ17)	15.87	
				397.3043	397.3027 (**Δ11**, Δ14, Δ17)	16.45	
				499.3724	499.3710 (Δ11, **Δ14**, Δ17)	16.45	

				357.273	357.2719 (Δ11, **Δ14**, Δ17)	16.3	
				539.4037	539.4022 (Δ11, Δ14, **Δ17**)	16.6	
				317.2417	317.2414 (Δ11, Δ14, **Δ17**)	16.6	
FA 20:2	599.4612	599.4601	16.4	459.3411	459.3398 (**Δ11**, Δ14)	17.08	**FA 20:2 (8, 11) FA 20:2 (11, 14)**
				399.3199	399.3188 (**Δ11**, Δ14)	17.68	
				499.3724	499.3709 (Δ11, **Δ14**)	17.38	
				359.2886	359.2877 (Δ11, **Δ14**)	16.92	
				417.2941	417.2929 (**Δ8**, Δ11)	17.38	
				441.3669	441.3661 (**Δ8**, Δ11)	17.53	
				457.3254	457.3241 (Δ8, **Δ11**)	17.68	
				401.3356	401.3347 (Δ8, **Δ11**)	17.53	
FA 20:1	601.4768	601.4762	17.46	459.3411	459.3398 (Δ11)	18.61	**FA 20:1 (9) FA 20:1 (11) FA 20:1 (13)**
				401.3356	401.3345 (Δ11)	19.05	
				487.3724	487.3712 (Δ13)	18.61	
				373.3043	373.3033 (Δ13)	18.76	
				431.3098	431.3082 (Δ9)	18.9	
				429.3669	429.3650 (Δ9)	19.51	
FA 22:6	619.4299	619.429	15.18	361.2315	361.2307 (**Δ4**, Δ7, Δ10, Δ13, Δ16, Δ19)	16.2	**FA 22:6 (4, 7, 10, 13, 16, 19)**
				517.3982	517.3970 (**Δ4**, Δ7, Δ10, Δ13, Δ16, Δ19)	17.11	
				401.2628	401.2618 (Δ4, **Δ7**, Δ10, Δ13, Δ16, Δ19)	15.92	
				477.3669	477.3656 (Δ4, **Δ7**, Δ10, Δ13, Δ16, Δ19)	16.35	
				441.2941	441.2930 (Δ4, Δ7, **Δ10**, Δ13, Δ16, Δ19)	15.52	
				437.3356	437.3345 (Δ4, Δ7, **Δ10**, Δ13, Δ16, Δ19)	15.92	
				481.3254	481.3243 (Δ4, Δ7, Δ10, **Δ13**, Δ16, Δ19)	15.78	
				397.3043	397.3033 (Δ4, Δ7, Δ10, **Δ13**, Δ16, Δ19)	15.65	
				521.3567	521.3555 (Δ4, Δ7, Δ10, Δ13, **Δ16**, Δ19)	15.65	
				357.273	357.2723 (Δ4, Δ7, Δ10, Δ13, **Δ16**, Δ19)	15.78	
				561.388	561.3866 (Δ4, Δ7, Δ10, Δ13, Δ16, **Δ19**)	15.78	
				317.2417	317.2410 (Δ4, Δ7, Δ10, Δ13, Δ16, **Δ19**)	15.78	
FA 22:5	621.4455	621.4438	15.96	403.2785	403.2776 (**Δ7**, Δ10, Δ13, Δ16, Δ19)	16.07	**FA 22:5 (7, 10, 13, 16, 19)**

				477.3669	477.3653 (**Δ7**, Δ10, Δ13, Δ16, Δ19)	16.97	
				443.3098	443.3090 (Δ7, **Δ10**, Δ13, Δ16, Δ19)	16.51	
				437.3356	437.3347 (Δ7, **Δ10**, Δ13, Δ16, Δ19)	16.51	
				483.3411	483.3401 (Δ7, Δ10, **Δ13**, Δ16, Δ19)	16.51	
				397.3043	397.3029 (Δ7, Δ10, **Δ13**, Δ16, Δ19)	16.07	
				523.3724	523.3715 (Δ7, Δ10, Δ13, **Δ16**, Δ19)	16.51	
				357.273	357.2722 (Δ7, Δ10, Δ13, **Δ16**, Δ19)	16.22	
				563.4037	563.4018 (Δ7, Δ10, Δ13, Δ16, **Δ19**)	16.51	
				317.2417	317.2409 (Δ7, Δ10, Δ13, Δ16, **Δ19**)	16.51	
FA 22:4	623.4612	623.4608	17	403.2785	403.2774 (**Δ7**, Δ10, Δ13, Δ16)	17.44	**FA 22:4 (7, 10, 13, 16)**
				479.3825	479.3810 (**Δ7**, Δ10, Δ13, Δ16)	18.35	
				443.3098	443.3088 (Δ7, **Δ10**, Δ13, Δ16)	17.59	
				439.3512	439.3498 (Δ7, **Δ10**, Δ13, Δ16)	17.9	
				483.3411	483.3397 (Δ7, Δ10, **Δ13**, Δ16)	17.14	
				399.3299	399.3185 (Δ7, Δ10, **Δ13**, Δ16)	17.44	
				523.3724	523.3712 (Δ7, Δ10, Δ13, **Δ16**)	17.59	
				359.2886	359.2878 (Δ7, Δ10, Δ13, **Δ16**)	17.44	
FA 22:1	629.5081	629.5066	20.13	487.3724	487.3714 (Δ13)	21.44	**FA 22:1 (13)**
				401.3356	401.3346 (Δ13)	19.32	

S11.4 PB reaction quality check with CD1

FAs in CD1 were labeled with both AMPE-tag i113 and i114. Therefore, for the CD1 sample, the 114/113 ratio should be 1. Examination of the 114/113 ratio of FAs from CD1 could offer further validation of this quantification method and work as a quality check of the reaction.

The quantitative analysis result of the FAs/FA isomers from the CD1 sample is plotted and demonstrated in Figure S33. 114/113 ratios of most data points are within the range of 0.8-1.2, as indicated by orange dash lines, which indicates uniformity of AMPE-tag i113 and i114 reacted with FAs in the same sample and testified to the reliability of this quantification method.

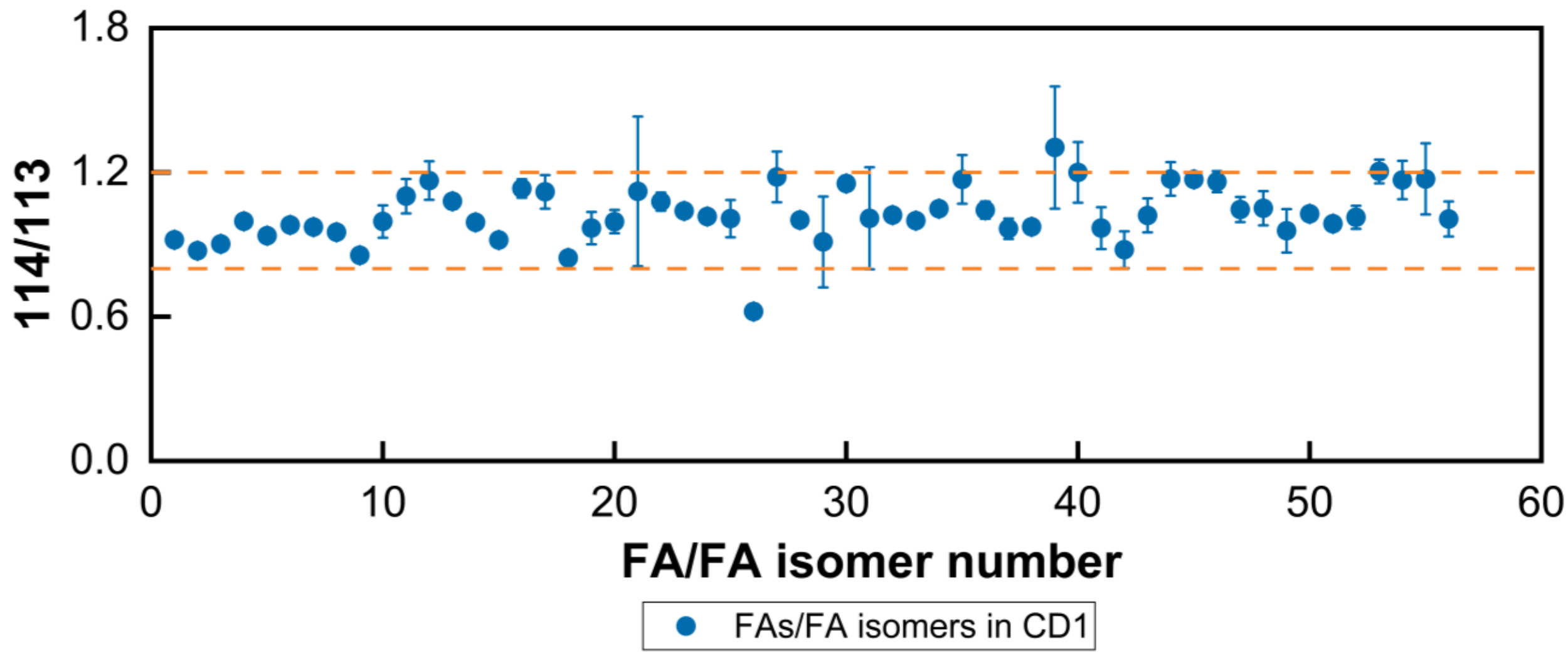

Figure S33. The collection of a 114/113 ratio of FAs and FA isomers from CD1. Most data points fall in the range of 114/113 ratio 0.8-1.2.

S11.5 Clarification of FA 18:1 positional isomers detected in VAT

FA 18:1(6) was included as an analytical standard in the method-validation experiments but was not detected as an assigned FA 18:1 positional isomer in the VAT samples. The FA 18:1 positional isomers identified in the VAT dataset were FA 18:1(9), FA 18:1(11), and FA 18:1(12). FA 18:1(6), also known as petroselinic acid, is more commonly found in plant-derived samples, particularly oils from species of the Apiaceae family (A. Hajib, S. E. Harkaoui, H. Choukri, *et al.*, *Biomolecules* **2023**, *13*, 1675). Therefore, its absence or low abundance in the animal samples analyzed here is not unexpected. These results distinguish the use of FA 18:1(6) in the standard-mixture validation experiments from the positional isomers detected in the VAT biological analysis.

S12. Correspondence between Δ-Position and n-Position Nomenclature

Table S5. Correspondence between Δ-position and n-position nomenclature of lipids in the study

Δ-Position Nomemclature	n-Position Nomemclature
**PC 18:1(9)/18:1(9)**	PC 18:1(n-9)/18:1(n-9)
PC 18:1(6)/18:1(6)	PC 18:1(n-12)/18:1(n-12)
PE 16:0/18:1(9)	PE 16:0/18:1(n-9)
PG 16:0/18:1(9)	PG 16:0/18:1(n-9)
PA 16:0/18:1(9)	PA 16:0/18:1(n-9)
FA 14:1(7)	FA 14:1(n-7)
FA 14:1(9)	FA 14:1(n-5)
FA 16:1(7)	FA 16:1(n-9)
FA 16:1(9)	FA 16:1(n-7)
FA 16:2(7,10)	FA 16:2(n-6, n-9)
FA 17:1(7)	FA 17:1(n-10)
FA 17:1(8)	FA 17:1(n-9)
FA 17:1(9)	FA 17:1(n-8)
FA 17:1(11)	FA 17:1(n-6)
FA 18:1(9)	FA 18:1(n-9)
FA 18:1(6)	FA 18:1(n-12)
FA 18:1(11)	FA 18:1(n-7)
FA 18:1(12)	FA 18:1(n-6)
FA 18:2(9,12)	FA 18:2(n-6, n-9)
FA 18:3(6,9,12)	FA 18:3(n-6, n-9, n-12)
FA 18:3(9,12,15)	FA 18:3(n-3, n-6, n-9)
FA 19:2 (9,12)	FA 19:2 (n-7, n-10)
FA 19:2 (10,13)	FA 19:2 (n-6, n-9)
FA 19:1(9)	FA 19:1(n-10)
FA 19:1(10)	FA 19:1(n-9)
FA 19:1(11)	FA 19:1(n-8)
FA 19:1(12)	FA 19:1(n-7)
FA 19:1(13)	FA 19:1(n-6)
FA 20:5(5,8,11,14,17)	FA 20:5(n-3, n-6,n-9,n-12,n-15)

FA 20:4(5,8,11,14)	FA 20:4(n-6, n-9, n-12, n-15)
FA 20:4(8,11,14,17)	FA 20:4(n-3, n-6, n-9, n-12)
FA 20:3(8,11,14)	FA 20:3(n-6, n-9, n-12)
FA 20:3(11,14,17)	FA 20:3(n-3, n-6, n-9)
FA 20:2(8,11)	FA 20:2(n-9, n-12)
FA 20:2(11,14)	FA 20:2(n-6, n-9)
FA 20:1(9)	FA 20:1(n-11)
FA 20:1(11)	FA 20:1(n-9)
FA 20:1(13)	FA 20:1(n-7)
FA 22:6(4,7,10,13,16,19)	FA 22:6(n-3, n-6, n-9, n-12,n-15, n-18)
FA 22:5(7,10,13,16,19)	FA 22:5(n-3, n-6, n-9, n-12,n-15)
FA 22:4(7,10,13,16)	FA 22:4(n-6, n-9, n-12,n-15)
FA 22:1(13)	FA 22:1(n-9)
FAEE 18:1(9)	FAEE 18:1(n-9)
FAEE 18:1(11)	FAEE 18:1(n-7)
CE 18:1(9)	CE 18:1(n-9)
CE 18:1(11)	CE 18:1(n-77)
CE 18:1(9,12)	CE 18:1(n-6, n-9)
TAG 18:1(9)/18:1(9)/18:1(9)	TAG 18:1(n-9)/18:1(n-9)/18:1(n-9)

Reference

(1) Liebisch, G.; Vizcaíno, J. A.; Köfeler, H.; Trötzmüller, M.; Griffiths, W. J.; Schmitz, G.; Spener, F.; Wakelam, M. J. O. Shorthand notation for lipid structures derived from mass spectrometry. *Journal of Lipid Research* **2013**, *54* (6), 1523-1530.

(2) Yang, T.; Tang, S.; Kuo, S.-T.; Freitas, D.; Edwards, M.; Wang, H.; Sun, Y.; Yan, X. Lipid Mass Tags via Aziridination for Probing Unsaturated Lipid Isomers and Accurate Relative Quantification**. *Angewandte Chemie International Edition* **2022**, *61* (39), e202207098.

(3) Zhigaltsev, I. V.; Winters, G.; Srinivasulu, M.; Crawford, J.; Wong, M.; Amankwa, L.; Waterhouse, D.; Masin, D.; Webb, M.; Harasym, N.; et al. Development of a weak-base docetaxel derivative that can be loaded into lipid nanoparticles. *Journal of Controlled Release* **2010**, *144* (3), 332-340.

(4) Angell, Y.; Chen, D.; Brahimi, F.; Saragovi, H. U.; Burgess, K. A Combinatorial Method for Solution-Phase Synthesis of Labeled Bivalent β-Turn Mimics. *Journal of the American Chemical Society* **2008**, *130* (2), 556-565.

(5) Fahlman, R. P.; Chen, W.; Overall, C. M. Absolute proteomic quantification of the activity state of proteases and proteolytic cleavages using proteolytic signature peptides and isobaric tags. *Journal of Proteomics* **2014**, *100*, 79-91.

(6) Chan, C.; Yin, H.; McKie, J. H.; Fairlamb, A. H.; Douglas, K. T. Peptoid inhibition of trypanothione reductase as a potential antitrypanosomal and antileishmanial drug lead. *Amino Acids* **2002**, *22* (4), 297-308.

(7) Zhang, W.; Zhang, D.; Chen, Q.; Wu, J.; Ouyang, Z.; Xia, Y. Online photochemical derivatization enables comprehensive mass spectrometric analysis of unsaturated phospholipid isomers. *Nature Communications* **2019**, *10* (1), 79.
(8) Lin, Q.; Li, P.; Fang, M.; Zhang, D.; Xia, Y. Deep Profiling of Aminophospholipids Reveals a Dysregulated Desaturation Pattern in Breast Cancer Cell Lines. *Analytical Chemistry* **2022**, *94* (2), 820-828.
(9) Zacharia, A.; Saidemberg, D.; Mannully, C. T.; Kogan, N. M.; Shehadeh, A.; Sinai, R.; Zucker, A.; Bruck-Haimson, R.; Goldstein, N.; Haim, Y.; et al. Distinct infrastructure of lipid networks in visceral and subcutaneous adipose tissues in overweight humans. *The American Journal of Clinical Nutrition* **2020**, *112* (4), 979-990.
(10) Sun, C.; Zhao, Y.-Y.; Curtis, J. M. The direct determination of double bond positions in lipid mixtures by liquid chromatography/in-line ozonolysis/mass spectrometry. *Analytica Chimica Acta* **2013**, *762*, 68-75.
(11) Young, R. S. E.; Flakelar, C. L.; Narreddula, V. R.; Jekimovs, L. J.; Menzel, J. P.; Poad, B. L. J.; Blanksby, S. J. Identification of Carbon-Carbon Double Bond Stereochemistry in Unsaturated Fatty Acids by Charge-Remote Fragmentation of Fixed-Charge Derivatives. *Analytical Chemistry* **2022**, *94* (46), 16180-16188.
(12) Xia, T.; Jin, X.; Zhang, D.; Wang, J.; Jian, R.; Yin, H.; Xia, Y. Alternative fatty acid desaturation pathways revealed by deep profiling of total fatty acids in RAW 264.7 cell line. *Journal of Lipid Research* **2023**, *64* (8), 100410.
(13) Stinson, C. A.; Xia, Y. A method of coupling the Paternò–Büchi reaction with direct infusion ESI-MS/MS for locating the C=C bond in glycerophospholipids. *Analyst* **2016**, *141* (12), 3696-3704, 10.1039/C6AN00015K.
(14) Virreira Winter, S.; Meier, F.; Wichmann, C.; Cox, J.; Mann, M.; Meissner, F. EASI-tag enables accurate multiplexed and interference-free MS2-based proteome quantification. *Nature Methods* **2018**, *15* (7), 527-530.
